# Peptide inhibitors of the anaphase promoting-complex that cause sensitivity to microtubule poison

**DOI:** 10.1371/journal.pone.0198930

**Published:** 2018-06-08

**Authors:** Scott C. Schuyler, Yueh-Fu Olivia Wu, Hsin-Yu Chen, Yi-Shan Ding, Chia-Jung Lin, Yu-Ting Chu, Ting-Chun Chen, Louis Liao, Wei-Wei Tsai, Anna Huang, Lin-Ing Wang, Ting-Wei Liao, Jia-Hua Jhuo, Vivien Cheng

**Affiliations:** 1 Department of Biomedical Sciences, College of Medicine, Chang Gung University, Kwei-Shan, Tao-Yuan, Taiwan; 2 Division of Colorectal Surgery, Department of Surgery, Chang Gung Memorial Hospital, Kwei-Shan, Tao-Yuan, Taiwan; Nanyang Technological University, SINGAPORE

## Abstract

There is an interest in identifying Anaphase Promoting-Complex/Cyclosome (APC/C) inhibitors that lead to sensitivity to microtubule poisons as a strategy for targeting cancer cells. Using budding yeast *Saccharomyces cerevisiae*, peptides derived from the Mitotic Arrest Deficient 2 (Mad2)-binding motif of Cell Division Cycle 20 (Cdc20) were observed to inhibit both Cdc20- and CDC20 Homology 1 (Cdh1)-dependent APC/C activity. Over expression of peptides *in vivo* led to sensitivity to a microtubule poison and, in a recovery from a microtubule poison arrest, delayed degradation of yeast Securin protein Precocious Dissociation of Sisters 1 (Pds1). Peptides with mutations in the Cdc20 activating KILR-motif still bound APC/C, but lost the ability to inhibit APC/C *in vitro* and lost the ability to induce sensitivity to a microtubule poison *in vivo*. Thus, an APC/C binding and activation motif that promotes mitotic progression, namely the Cdc20 KILR-motif, can also function as an APC/C inhibitor when present in excess. Another activator for mitotic progression after recovery from microtubule poison is p31^comet^, where a yeast predicted open-reading frame *YBR296C-A* encoding a 39 amino acid predicted protein was identified by homology to p31^comet^, and named Tiny Yeast Comet 1 (*TYC1*). Tyc1 over expression resulted in sensitivity to microtubule poison. Tyc1 inhibited both APC/C^Cdc20^ and APC/C^Cdh1^ activities *in vitro* and bound to APC/C. A homologous peptide derived from human p31^comet^ bound to and inhibited yeast APC/C demonstrating evolutionary retention of these biochemical activities. Cdc20 Mad2-binding motif peptides and Tyc1 disrupted the ability of the co-factors Cdc20 and Cdh1 to bind to APC/C, and co-over expression of both together *in vivo* resulted in an increased sensitivity to microtubule poison. We hypothesize that Cdc20 Mad2-binding motif peptides, Tyc1 and human hp31 peptide can serve as novel molecular tools for investigating APC/C inhibition that leads to sensitivity to microtubule poison *in vivo*.

## Introduction

One of the most successful classes of chemotherapeutic agents has been microtubule poisons [1]. Although the complete cellular mechanism is unclear, microtubule poisons are thought to act by creating a spindle checkpoint dependent delay in mitosis, which in turn leads to cells undergoing apoptosis [2–5]. Cancer cells may not respond to microtubule poison-based treatment, or may develop resistance to the therapy [1, 3, 5]. One of several possible contributing mechanisms hypothesized to explain microtubule poison resistance is that some cancer cells delay cell cycle progression in mitosis in a spindle checkpoint-dependent manner, but then have the ability to under go ‘mitotic slippage’, re-entering the cell cycle and progress through mitotic exit while bypassing cell death [3, 5]. ‘Mitotic slippage’ requires the activity of the cell cycle regulator APC/C and the essential mitotic co-factor Cdc20 to promote mitotic progression. This knowledge has contributed to a hypothesis that inhibiting APC/C^Cdc20^ might be an effective anti-cancer strategy in cells treated with microtubule poisons, which has led to studies targeting APC/C^Cdc20^ using small molecule APC/C inhibitors and by targeting Cdc20 using RNAi [2–12].

During normal cell cycle progression in mitosis, and after recovery from exposure to microtubule poisons, two critical activator motifs within Cdc20 that are required for promoting APC/C activity at metaphase and into anaphase are the C-box and KILR-motif within the N-terminal Domain of Cdc20 [13–17]. The C-box and Cdc20 KILR-motif reside adjacent to and within the Mad2-binding motif respectively. The Mad2-binding motif is a major target of the Mitotic Checkpoint Complex (MCC) when cells are exposed to microtubule poisons in order to achieve a cell cycle arrest. The MCC was first reported as a complex of Cdc20 and three human checkpoint proteins: Mad2, Mad3/Bub1-related (Mad3/BubR1), and Budding Uninhibited by Benzimidazole 3 (Bub3) [18]. Mad2 and Mad3/BubR1 bind directly to Cdc20, and this binding is required for APC/C^Cdc20^ inhibition [18–25]. Mitotic checkpoint activity and the MCC components are essential in mammals, even in cancer cells [26, 27].

Once spindle microtubules have attached properly to the kinetochores, the spindle checkpoint is ‘satisfied’, and Cdc20 is released from the MCC and degraded. Mad2 binding to Cdc20 is through a unique mechanism called the ‘safety-belt’, where Mad2 partially unfolds and then re-folds into what is called closed-Mad2 to form a binding loop around the Mad2-binding motif in Cdc20 [28–32]. This unique protein-protein interaction is thought to be reversed once the spindle checkpoint is ‘satisfied’ by the activity of the human p31^comet^ protein (originally named CMT2), which promotes the removal of Mad2 and disassembly of the MCC [33–42]. Thus, the inactivation and removal of Mad2 from Cdc20 by p31^comet^ is a critical step in promoting mitotic progression allowing cells to re-enter the cell cycle. How the process of mitotic progression is pre-maturely allowed to proceed in cancer cells as they undergo ‘mitotic slippage’ even after exposure to a microtubule poison remains unclear.

We hypothesized that the presence of an excess amount of an APC/C binding peptide, such as a Mad2-binding motif peptide derived from an activator region of Cdc20 such as the C-box or the KILR-motif, or an excess of a factor that promotes mitotic progression upon recovery from a microtubule poison, like p31^comet^, might act to disrupt the proper re-activation or regulation of the APC/C as the cells recovered from a mitotic arrest. Using budding yeast as a model, we have observed that Cdc20-derived peptides, the endogenous yeast Tyc1 protein identified by homology to human p31^comet^, and the human-derived p31^comet^ peptide called hp31can all serve as novel inhibitors of the APC/C that cause sensitivity to a microtubule poison by disrupting the ability of APC/C to interaction with the activating co-factors Cdc20 and Cdh1.

## Materials and methods

### Culture conditions for bacteria and yeast

For the production of high quality DNA for *in vitro* transcription/translation *E*. *coli* were grown in 50 mL LB containing 100 μg/mL ampicillin for 12–16 hours at 37 °C. Purified plasmid DNA, which was dissolved with 50 μL ddH_2_O or DEPC (diethyl pyrocarbonate)-treated water, was measured at OD_260/280_ and stored at -20 °C (Bioman, Inc., Taiwan). All mutant allele expression plasmids were constructed employing site-directed mutagenesis following the manufacturer’s protocol (Stratagene, USA). Yeast were cultured under standard conditions [43]. For large-scale yeast cultures to purify APC/C, yeast were cultured overnight in 10 mL YPD medium (20 g Dextrose, 20 g peptone, and 10 g yeast extract per liter) at 30 °C. 1.5 L of YPD medium in two Fernbach flasks was inoculated with 1 mL overnight culture and supplemented with 100 μg/mL ampicillin and 100 μg/mL streptomycin to prevent bacterial growth. Cell cycle arrest, collection of yeast cells and yeast cell extractions were all performed as previously described [44].

### Preparation of ^35^S-Pds1 and ^35^S-Cdc20 pure protein substrates

Full-length, radio-labeled protein purifications were preformed as previously described [44]. ^35^S-Pds1 was prepared in a 500 μL IVT/T (*In Vitro* Transcription/Translation) reaction (Promega, USA), which contained 400 μL rabbit reticulocyte lysate, 20 μL of L-^35^S-methionine (PerkinElmer, USA), 10 μg of plasmid DNA, and the remaining volume as nuclease-free ddH_2_O as recommended by the manufacturer. The reaction mixture was incubated for 2 hours at room temperature and gently mixed every 30 minutes. The reaction was added to pre-equilibrated IgG Sepharose beads (GE Healthcare, USA) in Bio-spin disposable chromatography columns (Bio-Rad, USA) for binding for 2 hours at room temperature. To ensure efficient binding, the beads were re-suspended several times every 30 minutes. After binding, the beads were washed with 5 mL wash buffer, containing 200 mM 4-(2-hydroxyethyl)-1-piperazineethanesulfonic acid (HEPES) at pH 8.0, 150 mM NaCl, 10% (v/v) glycerol, and 1 mM dithiothreitol (DTT). The ^35^S-Pds1 was cleaved from the beads using TEV (Tobacco Etch Virus) protease in 1x TEV buffer (Invitrogen, USA) for 2 hours at room temperature, and the mixture was re-suspended several times every 30 minutes to promote efficient cleavage. The elution was collected and concentrated by employing an Amicon Ultra filter column (Millipore, Ireland). The concentrated ^35^S-Pds1 was collected in a new tube, and stored at -20 °C.

^35^S-Cdc20 to measure the amount of protein produced in IVT/T reactions to confirm protein stability was prepared in a 20 μL IVT/T reactions, which contained 16 μL rabbit reticulocyte lysate, 0.8 μL of L-^35^S-methionine, 0.4 μg of plasmid DNA, and remaining volume of nuclease-free ddH_2_O, incubated for 2 hours at room temperature as recommended by the manufacturer (Promega, USA).

### APC/C enzyme assays

The production of Cdc20 or Cdh1 was prepared by IVT/T reactions, where 16 μL of rabbit reticulocyte lysate, 20 μM L-methionine, 0.4 μg of plasmid DNA, and a volume of nuclease-free ddH_2_O for a total volume of 20 μL. This freshly synthesized Cdc20 or Cdh1 protein was used directly in all APC/C assays after a 1-hour synthesis reaction at room temperature. The APC/C was purified for enzyme assays from 1 mL of TAP-tagged Cdc16 yeast extract (SCSY51) where extract was mixed with 50 μL 50:50 slurry magnetic beads (Invitrogen, Norway) for 2 hours at 4 °C. After incubation, the beads were washed with 3 mL APC/C wash buffer (200 mM of HEPES, 150 mM of NaCl and 10% (v/v) of glycerol). APC/C bound to IgG magnetic beads was used directly in the enzyme assays.

APC/C assays were performed by adding the reaction contents in following order: 4.3 μL of QAH buffer, 20 μL of rabbit reticulocyte lysate containing Cdc20 or Cdh1 with or without an additional volume of recombinant checkpoint protein(s) or inhibitor peptides, 2.4 μL ubiquitin aldehyde (Boston Biochem, USA), APC/C, 16 μL pre-charged E1/E2/ATP/ubiquitin mix, and 13.3 μL of ^35^S-Pds1 with QAH buffer (20 mM HEPES at pH 8.0, 100 mM NaCl, and 1 mM MgCl2). The E1/E2/ATP/ubiquitin mix were pre-charged by mixing 1 μL of E1 (Boston Biochem, USA), 1 μL of E2 (Boston Biochem, USA), 14.4 μL of ATP, 14.4 μL of ubiquitin aldehyde (Boston Biochem, USA), and 17.2 μL of QAH buffer and incubated for 15 minutes at room temperature. Approximately 15 μL of samples were taken out at each time point, and the reaction was stopped by the addition of 2x protein sample buffer (125 mM Tris-HCl pH 6.8, 4% SDS, 20% glycerol, and 0.002% bromophenol blue, with 10% (final v/v) 2-mercaptoethanol added in fresh directly before use).

For inhibitor titration and time-course experiments, recombinant Bub3, Mad2, Mad3, and the Mad3-Bub3 complex were expressed and purified as previously described [45, 46]. Inhibitory peptides were synthesized by standard procedures and purified to 99% purity by HPLC (Kelowna International Scientific Inc., Taiwan). For inhibition of APC/C the co-factor Cdc20 or Cdh1 was made fresh by IVT/T, and pure recombinant checkpoint proteins or inhibitor peptides were added to the IVT/T mixture and incubated for an additional hour at room temperature. In titrations, a dilution series of the checkpoint protein(s) were made before incubation with the Cdc20 co-factor. SDS-PAGE was performed using stand protocols (BioRad, USA).

### Biotinylated peptide pull-downs of purified APC/C and Western blots

For APC/C purification, the APC/C which contain Cdc16-TAP-tag was isolated from 2 mL arrested yeast lysate by 120 μL IgG sepharose 6 Fast Flow beads (GE Healthcare Life Sciences) and incubate for 2 h at 4 °C on a rotating wheel. Beads were collected washed with 5 mL of APC/C wash buffer (200 mM Hepes pH8.0, 150 mM NaCl, 10% glycerol). Beads were resuspended in 150 μL of APC/C wash buffer contain 17.6 units of AcTEV protease (Invitrogen, Carlsbad, CA) at 4°C for 3 h with pipetting every 20 minutes. The purified APC/C was collected in the flow-through.

For APC/C binding assays, 20 μM biotinylated peptides or 20 μM peptides only as a negative control were incubated with purified APC/C and 100 μL APC/C wash buffer which contains phosphatase inhibitors (200 mM Hepes pH 8.0, 150 mM NaCl, 10% glycerol, 10 mM NaF, 1 mM NaVO_4_, 5 mM Na-pyrophosphate, 10 mM 2-glycerol phosphate) for 1 h at 4 °C. The mixtures were applied to 120 μL streptavidin beads (Dynabeads MyOne Streptavidin T1, Invitrogen) for 30 min at 4 °C with gentle pipetting every 10 min. Beads were washed with 1 mL of PBST buffer (Phosphate buffer saline and 0.1% Tween 20) three times SDS-PAGE protein sample was added directly. For APC/C binding competition experiments, the biotinylated peptide and 10-fold excess non-biotinylated peptides were added to purified APC/C at same time.

Free D-biotin in 10% DMSO was used for the control binding assays. For pre-coating APC/C with biotin, a modified APC/C purification was performed. 2 mL yeast lysate was incubated with 120 μL IgG Sepharose beads and additional 20.4 μM free biotin or non-biotin solvent as a positive control was added and samples were incubated for 2 h at 4 °C on a rotating wheel. Purified pre-coated APC/C was then removed from the IgG Sepharose following the standard purification procedure. 20 μM biotinylated peptides or 20 μM peptides as a negative control were incubated with pre-coated APC/C and 100 μL APC/C wash buffer which contain phosphatase inhibitors for 1 h at 4 °C. In addition, 20 μM biotinylated peptide was added and incubated with pre-coated APC/C in a non-biotin containing solvent as positive control. Pre-coated APC/C binding procedures were same as above. For pre-coating streptavidin beads with biotin, streptavidin beads were washed in the presence of 10.2 nmol/mL D-biotin in PBST buffer or non-biotin solvent as a positive control before use. Pre-coated streptavidin beads were incubated with purified APC/C-peptide complexes with an extra 10.2 nM biotin for 30 minutes with gentle pipetting every 10 min at 4 °C.

Proteins were separated in SDS-PAGE gels and then were transferred to PVDF membrane. Blocking was performed by incubating samples in 20 mL PBST with 5% milk at 4 °C. Rabbit anti-tap (Thermo, CAB1001) was used as primary antibodies at a 1:1000 dilution and incubated for 2 hours at RT. Anti-rabbit HRP (Jackson, USA) was then used as secondary antibody at 1:5000 dilution and incubate for 1 hr at RT. Chemiluminescence signals were detected using an ImageQuant LAS 4000 (GE Healthcare Bio-Sciences, USA). For cell cycle timing Western blots anti-MYC and anti-Actin antibodies were employed (ABCAM, UK).

### Data analyses

For APC/C activity assays dried-gels were exposed on a phosphor screen cassette to detect radio-labeled protein. The phosphor screen was scanned by a Typhoon phosphor-imager (GE Healthcare Bio-Sciences, USA), and radio-labeled protein bands in gels were quantified by employing the ImageQuant software (GE Healthcare Bio-Sciences, USA) according to the manufacturer’s protocols. Data were expressed as mean ± standard deviation, and was analyzed using Student’s t-test. Graphs were created using Kaliedograph 4.0 (Synergy Software, USA) and estimates of best-fit curves of a 4-parameter Hill equation were generated to obtain estimates for the median inhibitory concentrations (IC_50_) and Hill coefficients (Hc) using IgorPro 6.01 (Wavemetrics, USA).

## Results

### A Cdc20 peptide containing the C-box motif and a region adjacent to the Mad2-binding motif can inhibit APC/C

We took initial steps to establish an *in vitro* APC/C assay to study potential inhibitory peptides by reproducing previously observed APC/C inhibition based on pure recombinant MCC components Mad2, Mad3, Bub3 and the Mad3-Bub3 complex to ensure that our assay was valid. Our observed results are in good agreement with previously published results (S1 and S2 Figs; S1, S2 and S3 Tables) [23–25, 47]. Having successfully established an *in vitro* assay to study potential APC/C inhibition, we reasoned that known mitotic APC/C activator amino acid motifs from Cdc20, such as the C-box motif and/or Mad2-binding motif, that are thought to function by binding and activating the APC/C, might also function as APC/C inhibitors *in vitro* when present in excess as peptides. To investigate this hypothesis, we employed chemically synthesized peptides at 99% purity and added them to the APC/C reactions at a high concentration of 220 μM to determine if they could inhibit APC/C activity.

**Fig 1:**
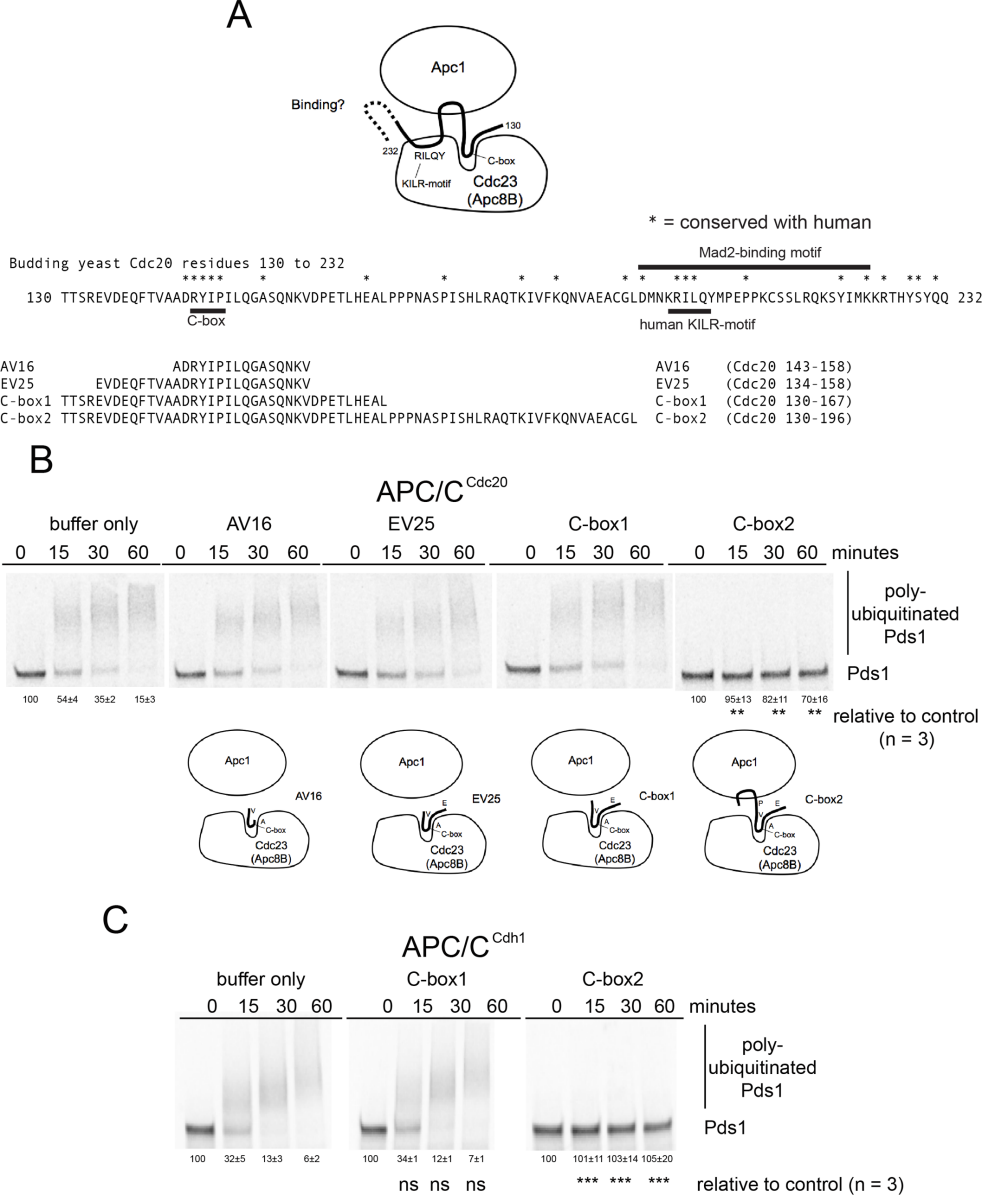
Functional analysis of C-box peptides sufficient to inhibit APC/C activity. A) A schematic diagram of the amino acid residues from the N-terminus of Cdc20 containing the region of the C-box, the KILR motif and the Mad2-binding motif [48]. The C-box and KILR motif regions, as well as the initial part of the Mad2-binding motif, interact with Cdc23 (APC8B in humans) based on structural analysis. The region in between the C-box and KILR-motif forms a loop that interacts with Apc1. The structure and potential binding site on the APC/C for the C-terminal portion of the Mad2-binding motif remains unknown (dashed line). The four C-box containing peptides AV16 (Cdc20 143–158), EV25 (Cdc20 134–158), C-box1 (130–167) or C-box2 (Cdc20 130–196) were designed to test their ability to inhibit APC/C^Cdc20^ activity. B) Phosphor-images of APC/C^Cdc20^ reaction time courses in the presence of buffer only or 220 μM of the C-box containing peptides AV16 (Cdc20 143–158), EV25 (Cdc20 134–158), C-box1 (130–167) or C-box2 (Cdc20 130–196). A significant level of APC/C^Cdc20^ inhibition was observed only in the presence of the C-box2 peptide (n = 3). C) Phosphor-images of APC/C^Cdh1^ reaction time courses in the presence of buffer or 220 μM C-box1 or C-box2 peptides. A significant level of APC/C^Cdh1^ inhibition was observed in the presence of the C-box2 peptide (n = 3).

We first tested the established APC/C binding and activating region of Cdc20 by designing and testing four C-box containing peptides for an ability to disrupt APC/C^Cdc20^ activity. To measure the level of inhibition of APC/C^Cdc20^ over time, the amount of Pds1 remaining was measured as a percentage of the initial value at the 0 time point. Data were analyzed by employing the Student’s *t*-test where the mean values of the percent of Pds1-remianing and the *p*-values relative to control reactions at each time point are indicated. Of the four peptides we designed, only a peptide called C-box2 (Cdc20 130–196) containing the region between the C-box and the Mad2-binding motif was observed to inhibit APC/C^Cdc20^ activity (Fig 1A and 1B). The C-box2 peptide also inhibited APC/C^Cdh1^ activity (Fig 1C). The C-box2 peptide contains the C-box and is extended to a region just to the N-terminus of the Mad2-binding motif adjacent to a highly conserved aspartic acid (D197) where in structural analyses this region interacts with the Apc1 subunit (see Fig 1A) [48]. Based on these observations we next chose to focus on yeast Mad2-binding motif peptides with homology to the previously defined human activator KILR-motif [16] to explore if they might inhibit APC/C activity. However, although there is some amino acid homology between yeast and human Cdc20 in this region, the exact amino acid residues that functionally define the budding yeast KILR-motif have not yet been established.

### The budding yeast KILR-motif is RILQY

By sequence alignment and amino acid homology alone, the budding yeast KILR-motif may be defined as 201-RILQ-204 (Fig 1A) [16]. However, the next residue, Tyrosine Y205 had previously been isolated in a genetic screen for spindle checkpoint by-pass alleles of Cdc20 [21], indicating this Y205 residue may be a target of the spindle checkpoint inhibition, and thus might also be a functional KILR-motif residue that contributes to APC/C activation. To clarify this possibility, we took the initial step to characterize several of the previously isolated spindle checkpoint by-pass mutant alleles of Cdc20 [21], and also Cdc20 mutants in the two highly conserved residues within the Mad2-binding motif, the aspartic acid at D197 of unknown function and the highly conserved Leucine L203 within the KILR-motif based on sequence alignment and homology [16]. All six mutant alleles were constructed within full-length Cdc20 by site-directed mutagenesis for analysis *in vitro* in the APC/C^Cdc20^ assay, along with a control mutant called Cdc20-CB (I147A, P148A), with mutations in two residues within the C-box that should not be able to promote APC/C activity (S4 Table) [13, 14, 15].

Our first goal was to measure the ability of the mutant alleles to promote APC/C activity *in vitro*. To ensure any observed change in APC/C activity reflected a change in function, and not a change in the amount of Cdc20 co-factor protein produced by IVT/T, the amounts of Cdc20 protein produced by IVT/T was measured, and no significant difference was observed (S4 Table). All seven alleles were then tested for their ability to promote APC/C activity. The control mutant Cdc20-CB (I147A, P148A), along with the Cdc20-127 by-pass allele (Y205N), and Cdc20-L203A mutant allele in the conserved KILR-motif all displayed decreased APC/C activity; but the by-pass allele Cdc20-106 (P209Q) was still active (S5 Table; S3A Fig). Excluding Cdc20-CB and Cdc20-L203A that had no APC/C activity, APC/C assays were repeated using 5 mutant alleles in the presence of 5.0 μM Mad2, a level of Mad2 that yields a high level of inhibition (see S1B Fig). Mutant by-pass allele proteins Cdc20-127 (Y205N) and Cdc20-106 (P209Q) were observed to promote APC/C activity at the same level as control reactions even in the presence of 5.0 μM Mad2 (S6 Table). In combination, the Y205 residue has been observed to be necessary to promote full APC/C activity and was also a target of Mad2 inhibition, which thus functionally defines Y205 as part of the yeast KILR-motif. In combination with sequence alignments, we define the budding yeast KILR-motif as 201-RILQY-205.

### Cdc20 peptides derived from the Mad2-binding motif inhibit APC/C

**Fig 2:**
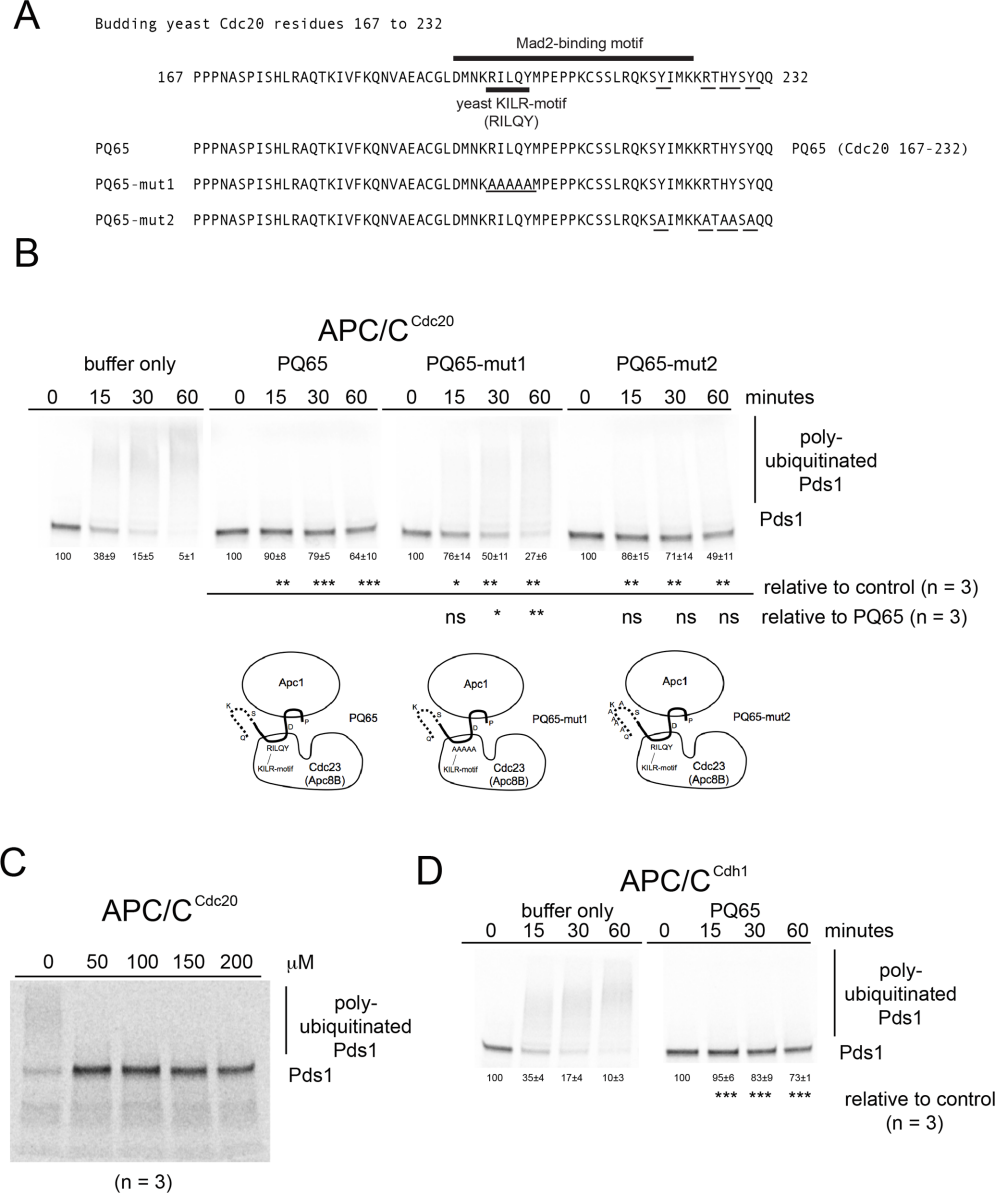
Functional analysis of PQ65 peptides sufficient to inhibit APC/C activity. A) A schematic diagram of the amino acid residues from the N-terminus of Cdc20 containing the region of the KILR motif and Mad2-binding motif. A 65 amino acid peptide PQ65 (Cdc20 169–232) was designed and synthesized around the most highly conserved regions within fungi. B) Phosphor-images of APC/C^Cdc20^ reaction time courses in the presence of buffer only or 220 μM of peptides. A significant level of APC/C^Cdc20^ inhibition was observed in the presence of the PQ65, PQ65-mut1 or PQ65-mut2 peptides (n = 3). Relative to the wild type PQ65 peptide, PQ65-mut1 peptide displayed a loss of inhibitory activity at the 30 and 60 minute time points (n = 3). C) Phosphor-image of a titration dilutions series using PQ65 where an IC_50_ of less than 50 μM was observed. D) Phosphor-images of APC/C^Cdh1^ reaction time courses in the presence of buffer or 220 μM PQ65 peptide, where a significant level of APC/C^Cdh1^ inhibition was observed (n = 3).

To initiate an investigation of Mad2-binding motif peptides centered around the yeast KILR-motif RILQY residues, a 65 amino acid residue peptide called PQ65 (Cdc20 169–232) with conserved regions among fungi was identified and contains an N-terminal region that interacts with Apc1 and a central region that interacts with Cdc23 (Fig 2A) [48]. When PQ65 was added to the APC/C^Cdc20^ reaction in excess the peptide was sufficient to inhibit APC/C^Cdc20^ activity (Fig 2B). Within the context of the PQ65 peptide, the yeast KILR-motif was observed to make a contribution to the inhibition of the APC/C^Cdc20^ activity based on an analysis where all five RILQY residues were changed to alanine AAAAA in PQ65-mut1. PQ65-mut1 displayed a significant loss in the ability to inhibit APC/C^Cdc20^ relative to the wild type PQ65 peptide at the 30 and 60 minute time points (Fig 2A and 2B). The next most highly conserved residues among fungi in the PQ65 peptide were towards the C-terminus where when all 5 residues were changed to alanine in PQ65-mut2, but no significant change was observed relative to wild type PQ65 with regard to APC/C inhibition (Fig 2A and 2B). A serial dilution was performed with the wild type PQ65 peptide and an estimated IC_50_ of less than 50 μM was observed (Fig 2C). The PQ65 peptide was also observed to inhibit APC/C^Cdh1^ (Fig 2D).

**Fig 3:**
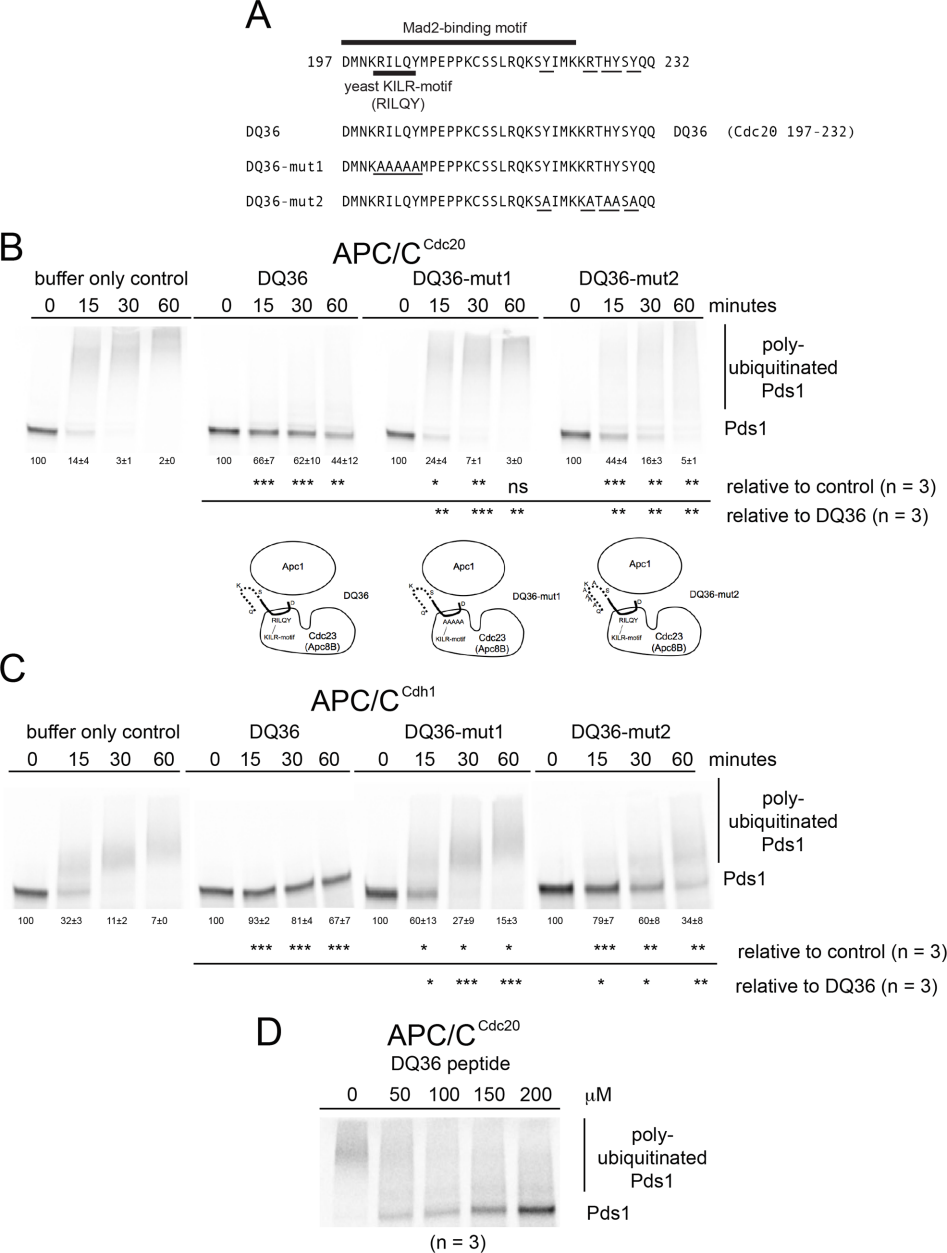
Functional analysis of DQ36 peptides sufficient to inhibit APC/C activity. A) A schematic diagram of the amino acid residues from the N-terminus of Cdc20 containing the region of the KILR motif and Mad2-binding motif. A 36 amino acid peptide DQ36 (Cdc20 197–232) was designed and synthesized around the most highly conserved regions within fungi. B) Phosphor-images of APC/C^Cdc20^ reaction time courses in the presence of buffer only or 220 μM of peptides. C) Phosphor-images of APC/C^Cdh1^ reaction time courses in the presence of buffer only or 220 μM of peptides. D) Phosphor-image of a titration dilutions series using DQ36 where an IC_50_ of about 150 μM was observed.

Within the PQ65 peptide the most conserved region is within the Mad2-binding motif that starts with the conserved aspartic acid residue D197 and extends towards the C-terminal residues. Thus, we chose to analyze the activity of the DQ36 peptide (Cdc20 197–232) derived from this region (Fig 3A). The DQ36 peptide was also sufficient to inhibit APC/C^Cdc20^ activity (Fig 3B). The RILQY residues of the yeast KILR-motif were observed to be essential for full inhibition by analyzing DQ36-mut1 where all 5 RILQY residues were changed to alanine AAAAA (Fig 3A and 3B). The C-terminal residues that are the most conserved among fungi were also observed to make a contribution to APC/C^Cdc20^ inhibition by analyzing DQ36-mut2 (Fig 3A and 3B). The DQ36 peptide was also observed to inhibit APC/C^Cdh1^ (Fig 3C) and, DQ36-mut1 and DQ36-mut2 displayed a similar loss in the ability to inhibit this APC/C^Cdh1^ enzyme activity relative to wild type DQ36 (Fig 3C). A titration was performed with the wild type DQ36 peptide and the IC_50_ for the peptide inhibitor was observed to be approximately 150 μM (Fig 3D), which is weaker than that observed for PQ65 (Fig 2C).

### Mad2-binding motif peptides bind to APC/C

**Fig 4:**
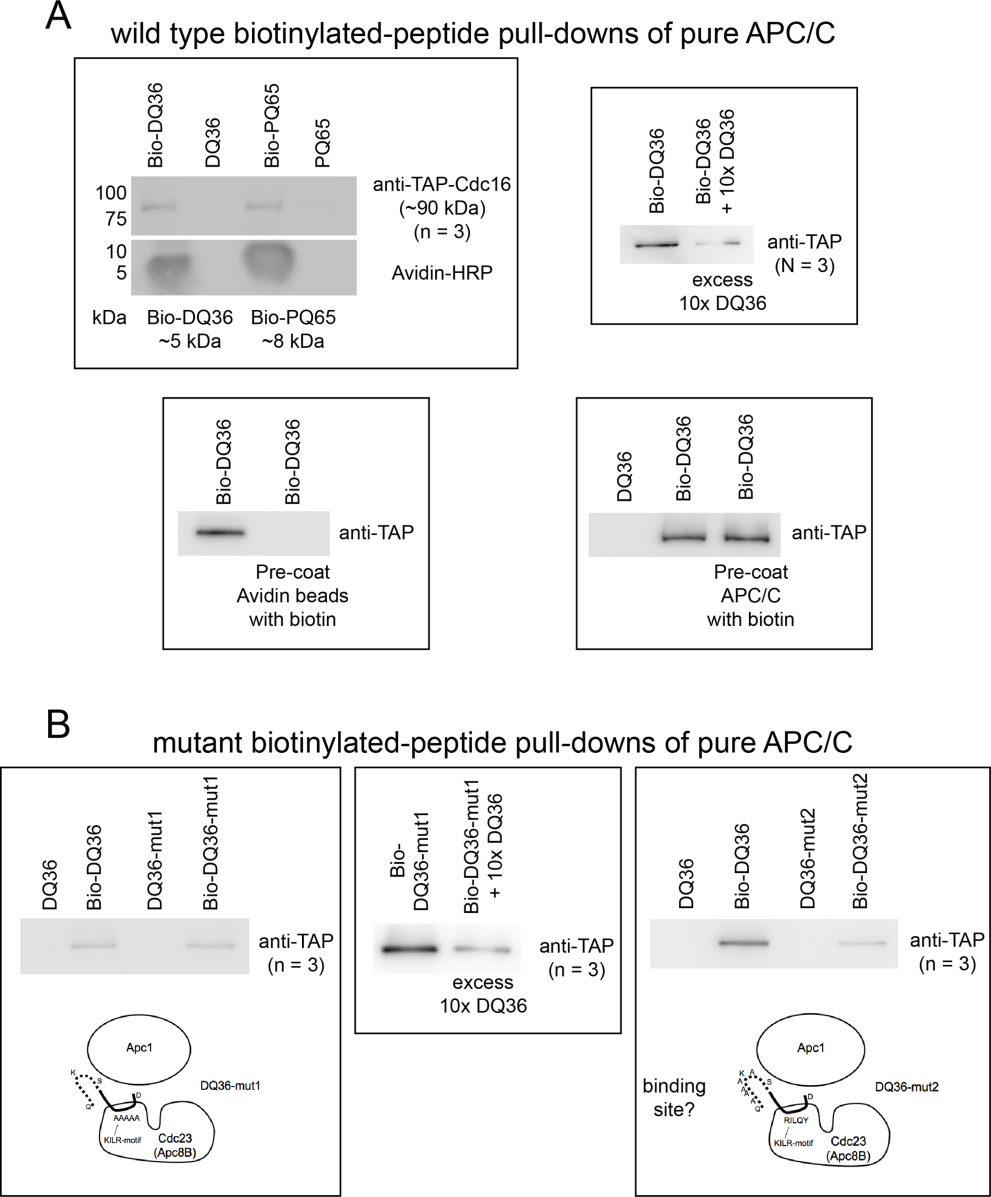
Mad2 binding motif peptides bind to APC/C. A) Wild type PQ65 and DQ36 pull-downs of APC/C at 20 μM peptide. Pre-coating the Avidin beads with biotin blocked the pull-down activity, but pre-coating APC/C with biotin had no effect. The addition of an excess of non-biotinylated DQ36 peptide was able to partially block the pull-down activity. B) Mutant DQ36-mut1 maintained the ability to pull-down APC/C, which was also partially blocked by an addition of excess DQ36 peptide. Mutant DQ36-mut2 was consistently observed to have partially lost APC/C binding activity relative to wild type DQ36 (n = 3).

To determine if the PQ65 and DQ36 peptides might target the APC/C for inhibition by binding directly to the APC/C, we mixed APC/C isolated from mitotic yeast extracts together with 20 μM of peptides containing an N-terminal biotin. These mixtures were then subjected to Avidin bead pull-downs and analyzed by Western blotting to detect APC/C and biotinylated peptides. Both biotinylated-PQ65 and biotinylated-DQ36 were able to pull-down purified APC/C (Fig 4A), where non-biotinylated peptides served as negative controls. Pre-coating the Avidin beads with biotin prevented the pull-downs (Fig 4A), but pre-coating the APC/C with biotin had no effect (Fig 4A). The addition of an excess of non-biotinylated DQ36 peptide was also observed to be able to partially compete against the binding of biotinylated-DQ36 with APC/C (Fig 4A).

We further analyzed the ability of biotinylated-DQ36-mut1 and biotinylated-DQ36-mut2 peptides to bind to APC/C. The biotinylated-DQ36-mut1 peptide bound to APC/C at the same level as biotinylated-DQ36 (Fig 4B), where the presence of excess DQ36 was also able to compete for the binding of the biotinylated-DQ36-mut1 peptide (Fig 4C and 4B). The ability of biotinylated-DQ36-mut1 peptide to still bind with the APC/C was notable because this peptide has lost the ability to inhibit the APC/C (see Fig 3B). Biotinylated-DQ36-mut2 consistently displayed weaker binding (Fig 4B), which correlates with the observation that it is also a weaker inhibitor.

### Conserved N- and C-terminal residues outside of the KILR-motif make contributions to APC/C inhibition and binding

**Fig 5:**
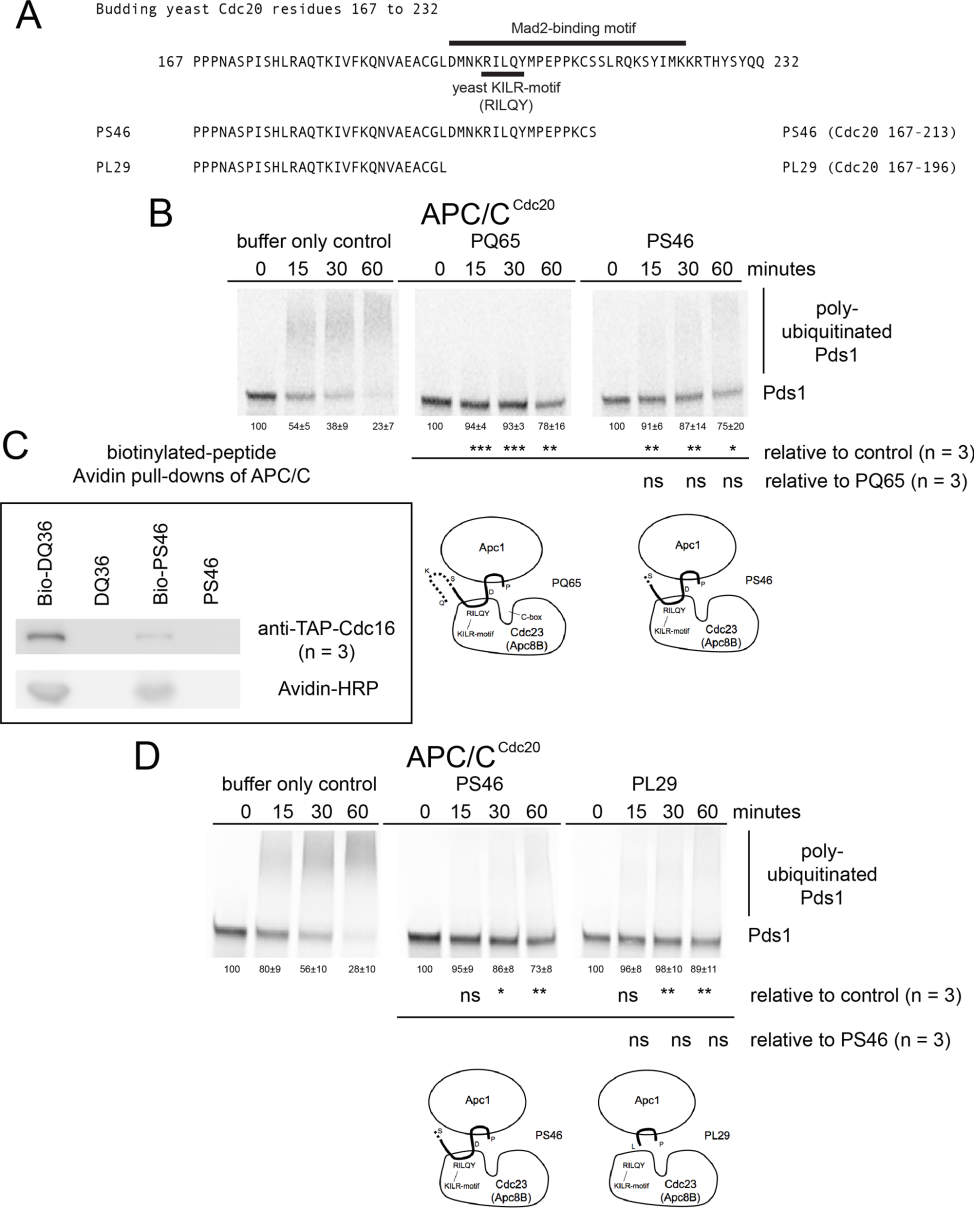
Functional analysis of PS46 and PL29 peptides sufficient to inhibit APC/C activity. A) A schematic diagram of the amino acid residues from the N-terminus of Cdc20 containing the region of the KILR motif and Mad2-binding motif. A 46 amino acid peptide PS46 (Cdc20 167–213) containing the N-terminal region adjacent to the KILR-motif region was designed and tested, along with the 29 amino acid peptide PL29 (Cdc20 167–196) that only contained the N-terminal region in between the C-box and the KILR-motif. B) Phosphor-images of APC/C^Cdc20^ reaction time courses in the presence of buffer only or 220 μM of peptides PQ65 and PS46. C) PS46 was consistently observed to have weaker APC/C binding activity relative to wild type DQ36 (n = 3). D) Phosphor-images of APC/C^Cdc20^ reaction time courses in the presence of buffer only or 220 μM of peptides PS46 and PL29.

To further map which regions within PQ65 were sufficient to disrupt APC/C^Cdc20^ activity, an N-terminal 46 amino acid peptide called PS46 (Cdc20 167–213), which still contained the yeast KILR-motif region was synthesized and was observed to inhibit APC/C^Cdc20^ (Fig 5A and 5B). The PS46 peptide was also able to bind to the APC/C directly, although consistently weaker binding was observed relative to DQ36 (Fig 5C). We also tested a smaller peptide PL29 (Cdc20 167–196) that does not contain the KILR-motif defined as the 29 amino acid residues in the region in between the C-box and the Mad2 Binding motif (Fig 5A). PL29 also displayed the ability to inhibit APC/C^Cdc20^ activity even in the absence of the Mad2 binding motif or yeast KILR motif regions (Fig 5D). We also attempted to analyze the ability of PL29 to bind with the APC/C. However, we observed that the N-terminal biotinylated form the PL29 peptide (Bio-PL29) would not bind to Avidin beads when in the presence of the APC/C, even though the Bio-PL29 peptide would bind to Avidin in the absence of the APC/C (S4 Fig).

**Fig 6:**
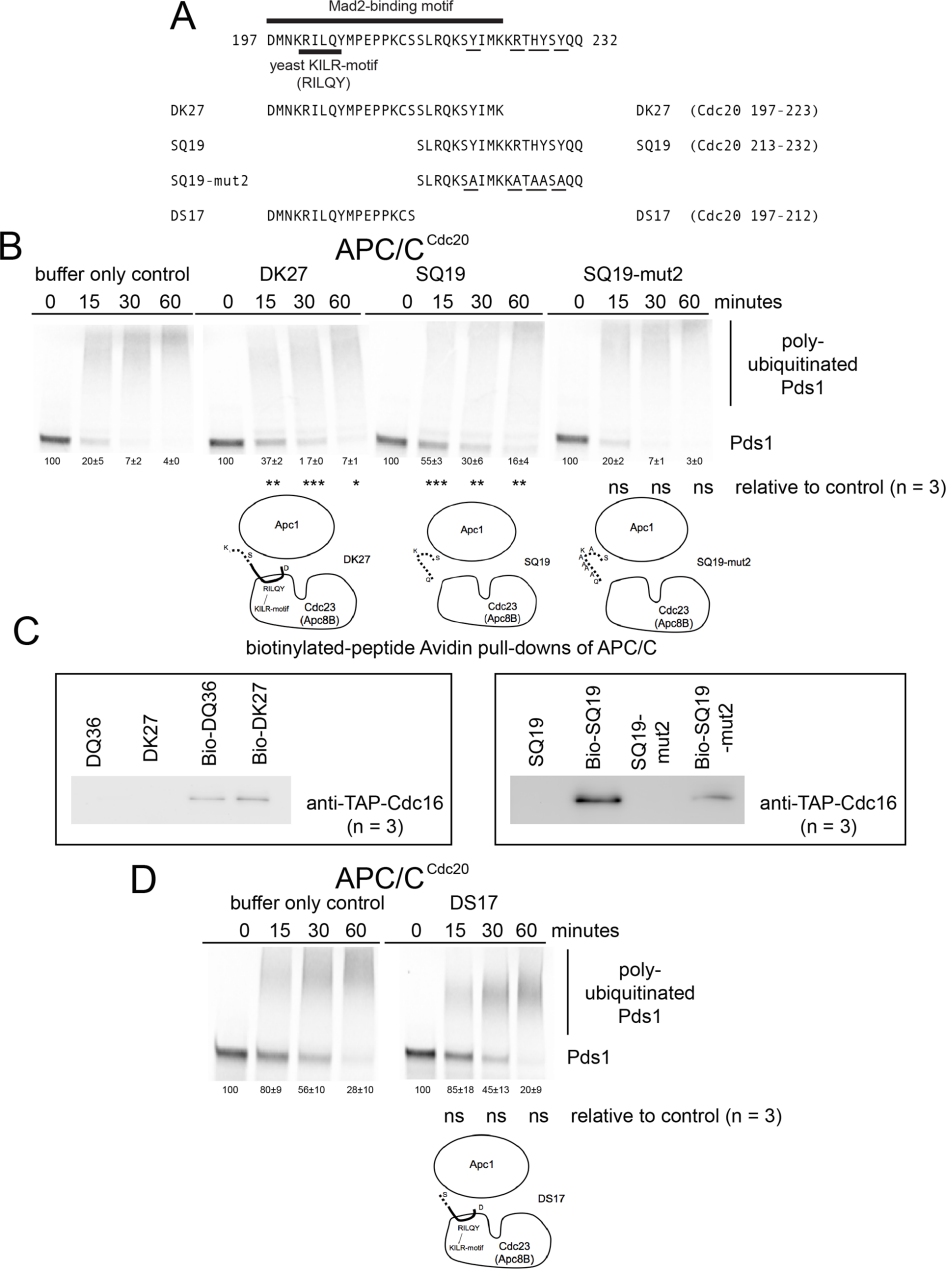
Functional analysis of DK27, SQ19 and DS17 peptides for APC/C inhibition activity. A) A schematic diagram of the amino acid residues from the N-terminus of Cdc20 containing the region of the KILR motif and Mad2-binding motif. A 27 amino acid peptide DK27 (Cdc20 197–223) containing KILR-motif region and only part of the C-terminal conserved region was designed and tested, along with the 19 amino acid peptide SQ19 (Cdc20 213–232) that only contained the C-terminal region, and the smaller DS17 (Cdc20 197–212) centered only on the KILR-motif and Mad2-binding motif. B) Phosphor-images of APC/C^Cdc20^ reaction time courses in the presence of buffer only or 220 μM of peptides DK27, SQ19 and SQ19-mut1. C) DK27 and SQ19 peptides displayed the ability to bind APC/C similar to the control peptide DQ36, but SQ19-mut2 was consistently observed to have much weaker APC/C binding activity relative to wild type SQ19 (n = 3). D) Phosphor-images of APC/C^Cdc20^ reaction time courses in the presence of buffer only or 220 μM of the peptide DS17 (Cdc20 197–212), where no inhibition was observed.

We also tested the defined Mad2-binding motif peptide DK27 (Cdc20 197–223) for the ability to inhibit APC/C (Fig 6A). The DK27 peptide displayed a diminished ability to inhibit APC/C^Cdc20^ although a significant difference with the buffer only control was still observed (Fig 6B). The DK27 peptide also retained the ability to bind with the APC/C (Fig 6C). We also tested a peptide containing the C-terminal 19 amino acids by analyzing the activity of the SQ19 (Cdc20 213–232) peptide and SQ19-mut2 peptide, where the most highly conserved 5 residues among fungi were altered to alanine in the mutant peptide (Fig 6A). The SQ19 peptide displayed a low level of inhibitory activity, and like DK27, was still able to bind to the APC/C (Fig 6C). The SQ19-mut2 peptide no longer displayed any ability to inhibit APC/C and also displayed only a very weaker amount of APC/C binding activity, indicating that the C-terminal conserved residues make a contribution to binding and promoting inhibition of the APC/C (Fig 6C). However, the APC/C subunit binding-site for this region of the Cdc20 remains unknown. We also analyzed the ability of the DS17 (Cdc20 197–212) peptide derived from the N-terminal portion of the DQ36 peptide (Fig 6A) to determine if it retained the ability to inhibit APC/C, but no significant inhibition was observed (Fig 6D). This indicates that the C-terminal SQ19 region within the DQ36 peptide is a contributing factor for the observed ability of the DQ36 peptide to inhibit APC/C (Fig 3B).

### Over expression of Mad2-binding motif peptides *in vivo* leads to sensitivity to microtubule poison and a delay in Pds1 degradation

**Fig 7:**
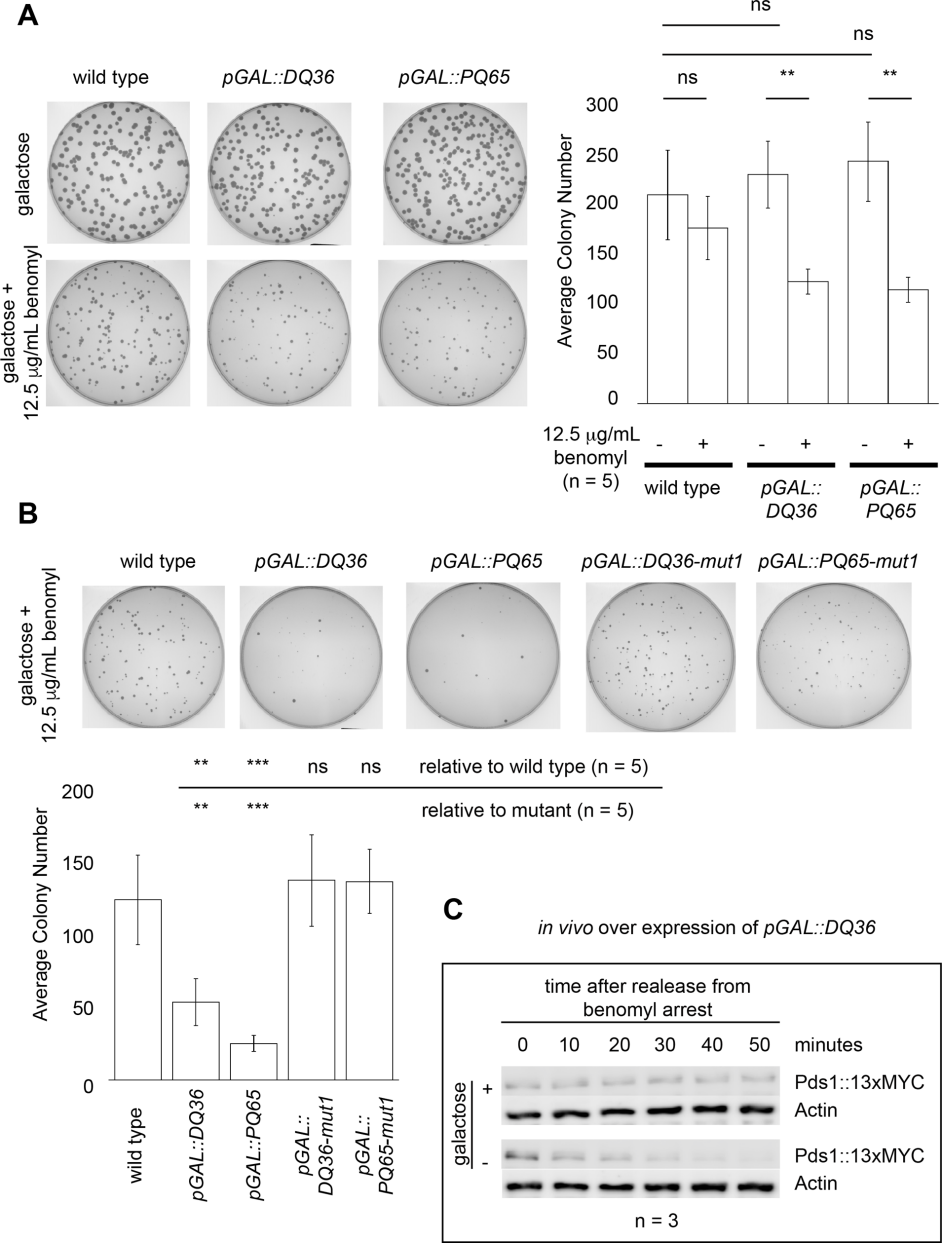
Mad2-binding motif peptides induce sensitivity to the microtubule poison benomyl when over expressed *in vivo*, which requires the KILR-motif, and leads to a delay in Pds1 degradation upon recovery from benomyl. A) Over expression of PQ65 or DQ36 *in vivo* leads to sensitivity to the microtubule poison benomyl. There was no significant difference observed in the absence of benomyl. B) The benomyl sensitivity phenotype was not observed when PQ65-mut1 or DQ36-mut1 peptides were over expressed where all 5 KILR-motif residues RILQY were changed to AAAAA. C) Over expression of DQ36 caused a delay in Pds1 degradation after cell cycle recovery from the microtubule poison benomyl as detected by Western blotting. Pds1 protein was detected using an anti-MYC antibody where the endogenous Pds1 had been epitope-tagged at the C-terminus with 13xMYC. An anti-actin antibody was used to probe for the levels of actin in each sample as a loading control.

Our *in vitro* biochemical observations indicated that in excess Mad2-binding motif peptides inhibit APC/C activity. There is a long-term interest in identifying APC/C inhibitors that give rise to sensitivity to microtubule poisons *in vivo*. To determine if a Cdc20 Mad2-binding motif peptide was sufficient to induce a microtubule poison sensitivity phenotype *in vivo*, the DQ36 and PQ65 peptides were over expressed under the control of a galactose inducible *pGAL1-10* promoter that had been integrated as single copy into the yeast genome together with the peptide open reading frames (ORF). The expressed proteins were engineered to contain an N-terminal nuclear localization signal (MSPKKKRKVAS) along with a short amino acid ‘linker’ in order to target the nuclear pool of the APC/C because yeast undergo a closed mitosis without nuclear envelope breakdown [49, 50]. In the presence of galactose, no change in colony number or colony morphology was observed (Fig 7A and S5 Fig). With the addition of 12.5 μg/mL of the microtubule poison benomyl, over expression of either DQ36 or PQ65 induced a significant sensitivity to benomyl as measured by the average colony numbers per plate (Fig 7A and S5 Fig). This phenotype was suppressed by mutating the yeast KILR-motif residues RILQY to AAAAA (Fig 7B) providing a direct correlation between our observed *in vitro* APC/C inhibition that requires the yeast KILR-motif RILQY residues and an *in vivo* microtubule poison sensitivity phenotype.

**Fig 8:**
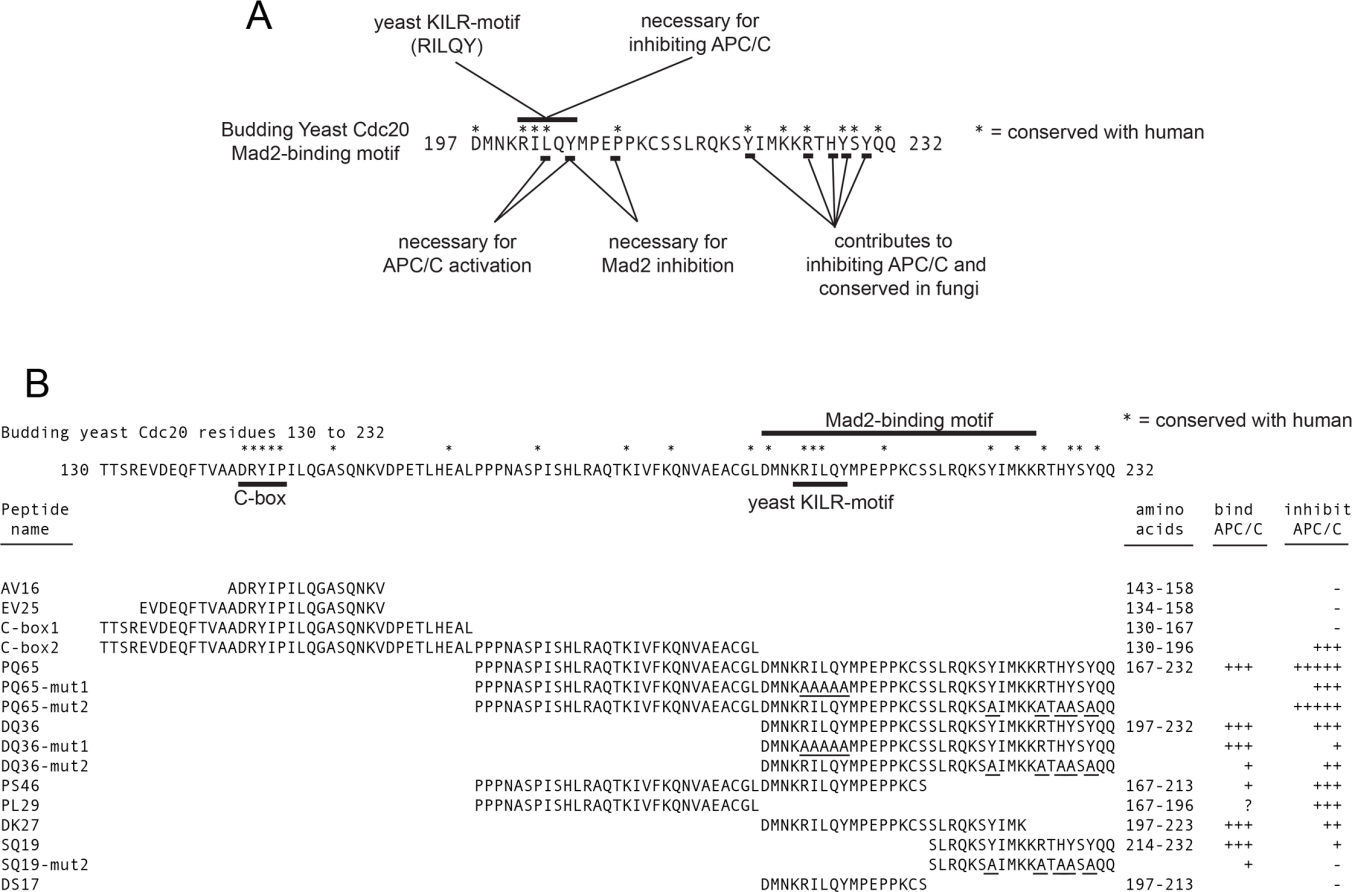
Summary of the functional and APC/C inhibitor analysis on the C-box and Mad2-binding motif regions of Cdc20. A) The budding yeast KILR-motif is defined as RILQY. B) A summary of the APC/C^Cdc20^ inhibition and APC/C binding data. The ability to inhibit the APC/C resides in both the N- and C-terminal regions of the PQ65 peptide, where both PL29 and SQ19 peptides contain an ability to inhibit APC/C even in the absence of the core KILR-motif region. In the DQ36-mut1 peptide, although the peptide does not fully inhibit the APC/C, the peptide retains the full ability to bind APC/C. In the DQ36-mut2 and SQ19-mut2 peptides there is a correlation between the loss of APC/C binding activity and a loss in the ability to inhibit APC/C.

We corroborated the *in vivo* phenotype by arresting cells in liquid culture in benomyl in the presence or absence of galactose to over express DQ36 *in vivo* and then observed the levels of Pds1 over time after washing out the benomyl, which would allow the cells to re-enter the cell cycle and activate APC/C^Cdc20^. Upon achieving a 90%+ level of mitotic arrest, as judged by counting the number of large-budded cells, the benomyl was washed out and samples were collected for Western blot analyses (Fig 7C). We observed that by 30–40 minutes the Pds1 signal was diminished in the absence of DQ36 peptide over expression, but that in cells over expressing the DQ36 peptide the levels of Pds1 remained elevated up to about 50–60 minutes. Thus, an APC/C activating motif that promotes mitotic progression, namely a peptide containing the yeast Cdc20 KILR-motif RILQY, can also function as an APC/C inhibitor that induces sensitivity to a microtubule poison *in vivo* and delays the degradation of Pds1 when present in excess (Fig 8).

### Tyc1 is homologous to p31^comet^ and causes sensitivity to microtubule poison when over expressed *in vivo*

We have observed that the Mad2-binding motif peptide DQ36 inhibits APC/C in a KILR-motif dependent manner, causes a benomyl sensitivity phenotype, and also delays the degradation of Pds1 upon recovery from a benomyl-induced mitotic arrest. In human cells, a known APC/C activator for mitotic progression after microtubule poison induced mitotic arrest is p31^comet^, where, to date, a potential homolog of p31^comet^ has not been identified in budding yeast. Because over expression of DQ36 led to delayed degradation of Pds1, were curious to try and identify a potential homologue of p31^Comet^ in yeast. A human p31^comet^ 274 amino acid sequence was used for a Basic Local Alignment Search Tool (BLAST) search of the budding yeast proteome using standard settings [51]. Several budding yeast proteins, or predicted proteins from putative ORFs, displayed only small regions of very low levels of homology.

**Fig 9:**
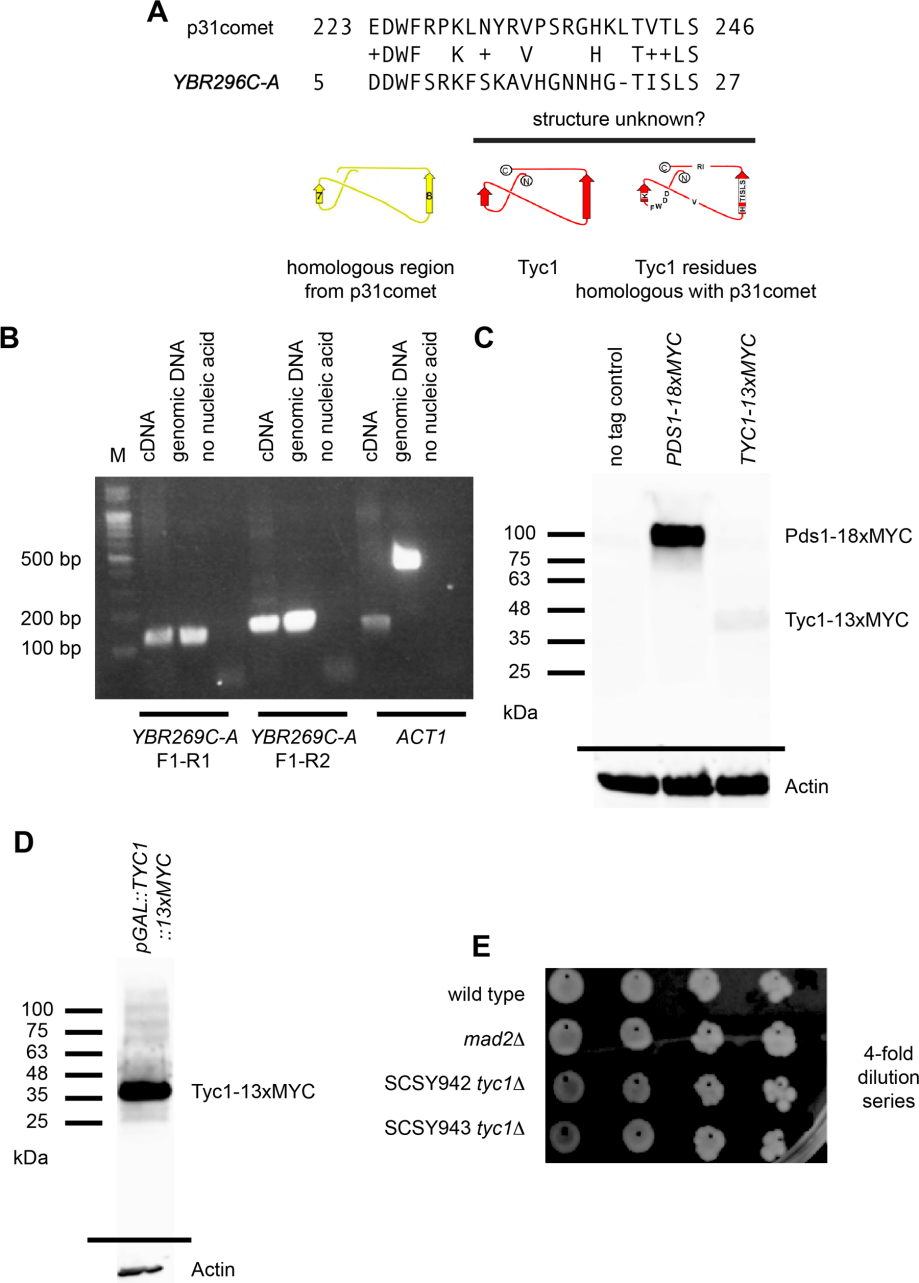
Identification of Tiny Yeast Comet 1 (*TYC1*). A) BLAST search result between a segment of human p31^comet^ and the predicted ORF *YBR296C-A*. The homologous region from p31^comet^ was previously determined to mimic the “safety-belt” wrapping around the “pseudo-Cdc20” tail and contains β-sheet 7 and 8 from the structure. The structure of the predicted protein product from *YBR296C-A* is unknown. B) Detection of an endogenous mRNA product by RT-PCR. C) Detection of an endogenous epitope-tagged protein product produced from the native genomic promoter detected by Western blot. D) Detection of an over expressed epitope tagged protein from the genomic locus. E) A *tyc1Δ* strains are viable, where two independent clones are shown in a dilution series.

We focused on the homologous predicted protein from the predicted open reading frame *YBR296C-A* because in a previous gene-trapping screen *YBR296C-A* was identified as making an mRNA gene product [52], and in a previous proteomics screen the predicted 39 amino acid protein product had been identified as a peptide that binds with Mad2, providing evidence that a real protein product exists in the cell and that it may interact directly or indirectly with Mad2 [53]. Notably, the *YBR296C-A* predicted protein contains two amino-acid motifs that appear to be highly conserved in the region of p31^comet^ that mimics the “safety-belt” wrapping around the “pseudo-Cdc20” tail, which is analogous to the region in closed-Mad2 that targets the Mad2-binding motif of Cdc20 (Fig 9A) [28–32, 34, 35, 36]. To confirm that *YBR296C-A* encodes a true gene product a native mRNA was detected by PCR (Fig 9B) and a low level of protein with an epitope tag at the C-terminus and expressed under the native genomic promoter was detected by Western blot (Fig 9C). The C-terminal tagged protein could also be over expressed when placed under the control of the galactose-inducible *pGAL1-10* promoter in a single copy at the endogenous locus in the chromosome as detected by Western blot (Fig 9D). We named this non-essential gene Tiny Yeast Comet 1 (*TYC1*), where cells deleted for *TYC1* (*tyc1Δ*) remained viable (Fig 9E).

**Fig 10:**
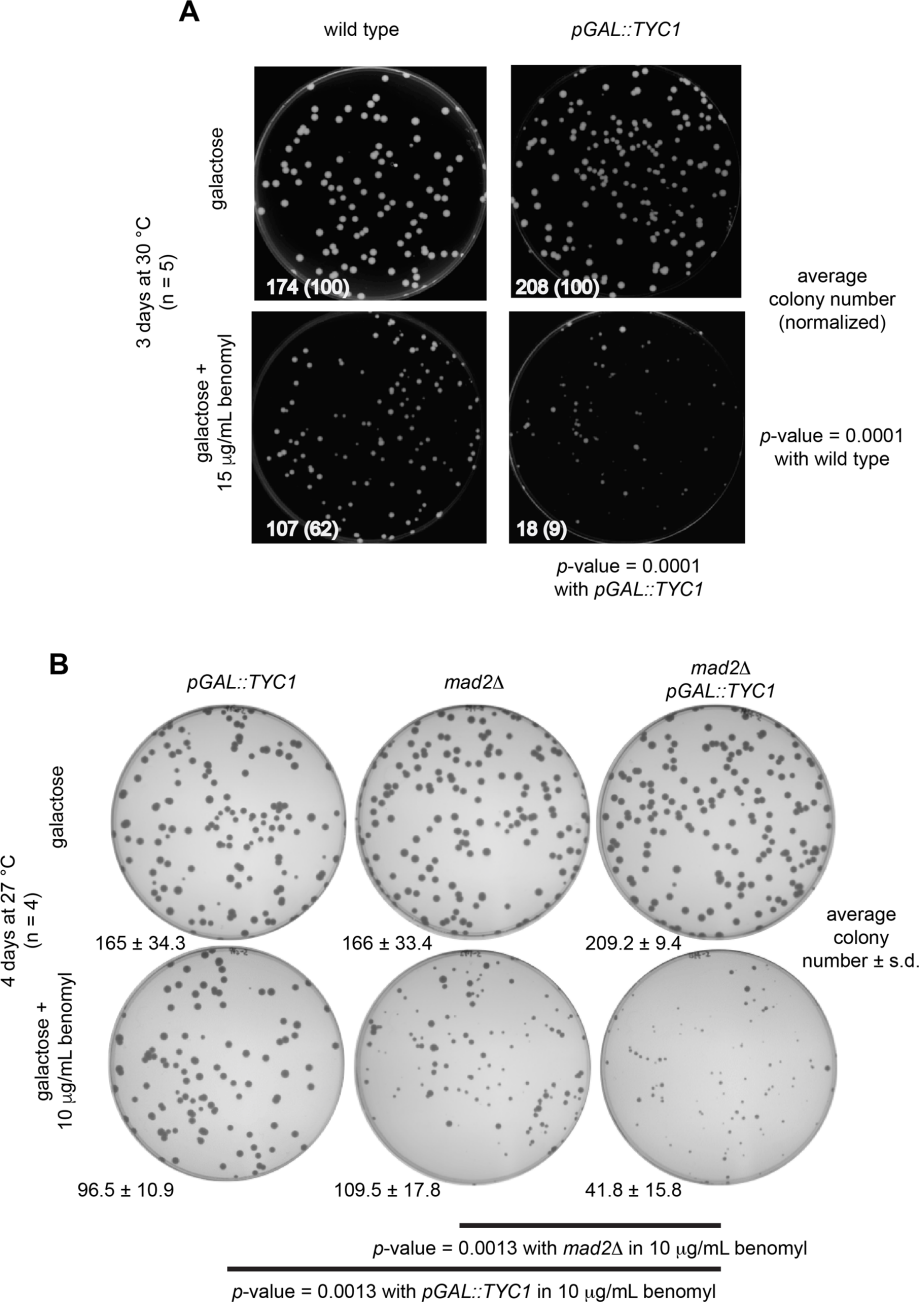
Over expression of Tyc1 induced sensitivity to the microtubule poison benomyl. A) Over expression of Tyc1 gave rise to a benomyl sensitive phenotype. B) Over expression of Tyc1 increased benomyl sensitivity in a *mad2Δ* background.

Over expression of native Tyc1 protein under the *pGAL1-10* promoter gave rise cells that displayed a sensitivity to benomyl, which struck us as similar to the Cdc20-derived Mad2-binding motif peptide over-expression phenotype, where a significant difference in colony number was observed in the presence of 15 μg/mL benomyl at 30 °C for 3 days specifically only when Tyc1 was over expressed (Fig 10A). Cells over expressing Tyc1 in a *mad2Δ* background grown on 15 μg/mL benomyl at 30 °C for 3 days were inviable, but at the lower concentration of 10 μg/mL benomyl at 27 °C for 4 days cells were viable and also displayed a sensitized benomyl phenotype (Fig 10B).

### Tyc1 inhibits APC/C *in vitro*

**Fig 11:**
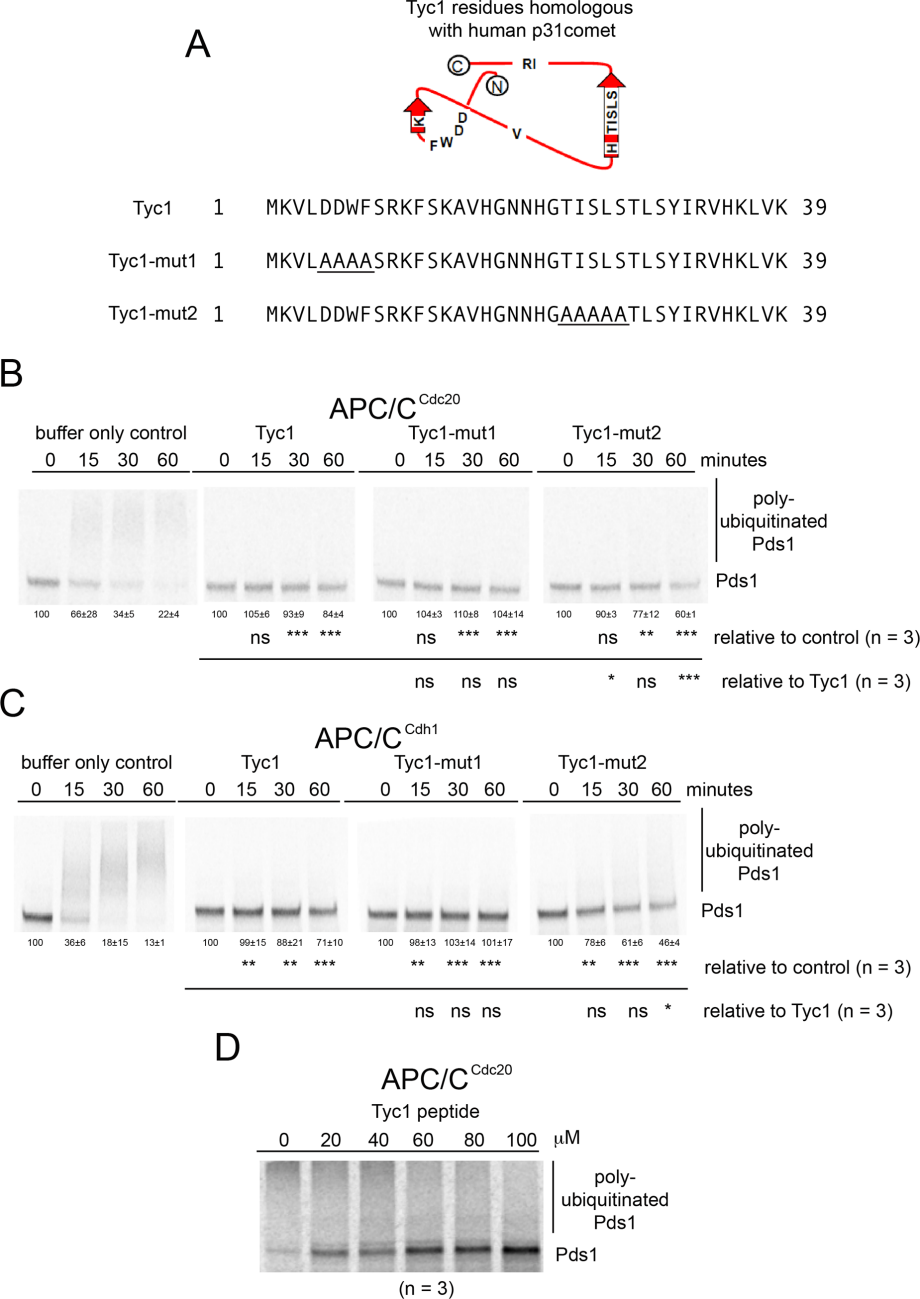
Tyc1 inhibits APC/C activity. A) Tyc1 proteins tested for their ability to inhibit APC/C activity. Tyc1-mut1 has 4 conserved amino acid residues changed to alanine. Tyc1-mut2 has 5 conserved residues changed to alanine. B) Phosphor-images show that 220 μM Tyc1 inhibits APC/C^Cdc20^ activity. Tyc1-mut1 also fully inhibited APC/C^Cdc20^. Tyc1-mut2 partially lost the ability to inhibit APC/C^Cdc20^. C) 220 μM Tyc1 inhibits APC/C^Cdh1^ activity. Tyc1-mut1 also fully inhibited APC/C^Cdh1^. Tyc1-mut2 displayed a slight loss in the ability to inhibit APC/C^Cdh1^. D) Phosphor-image of a titration dilutions series using Tyc1 where an IC_50_ of about 50 μM was observed.

Because we had observed a sensitivity to benomyl when Tyc1 was over expressed that was similar to the phenotype displayed by the over expression of Mad2-binding motif peptides, we decided to test if Tyc1 might also inhibit APC/C *in vitro* when present in excess. Unexpectedly, Tyc1 inhibited both APC/C^Cdc20^ and APC/C^Cdh1^ (Fig 11A and 11B). Tyc1 was identified based on conserved amino acid residues to a small segment of the human p31^comet^ protein. To determine if the observed inhibition was dependent on these conserved residues we tested the inhibitory activity of two different mutant Tyc1 proteins. Tyc1-mut1 with the conserved DDWF residues changed to AAAA did not display any loss in inhibition (Fig 11A and 11B). Tyc1-mut2 containing 5 amino acid residues TISLS changed to AAAAA in the C-terminus displayed a partially significant decrease in inhibition in both APC/C^Cdc20^ and a slight decrease in inhibiting APC/C^Cdh1^ (Fig 11A and 11B). To approximate the strength of inhibition, titrations were performed where wild type Tyc1 was observed to inhibit APC/C ^Cdc20^ with an approximate IC_50_ of 50 μM (Fig 11D).

Since p31^comet^ participates in promoting ubiquitination of Cdc20, we also tested if Tyc1 affected the level of APC/C^Cdc20^ activity when Cdc20 was used as the target substrate. Tyc1 also inhibits APC/C^Cdc20^ and APC/C^Cdh1^ when Cdc20 was used as the target substrate (S6 Fig). Since the region of homology with p31^comet^ is within the Mad2-like “safety-belt” loop that in closed-Mad2 targets the Mad2-binding motif of Cdc20, we also tested if Tyc1 could inhibit the activity of the Cdc20-106 (P209Q) spindle checkpoint by-pass allele of Cdc20 that can still promote APC/C activity but was not inhibited by Mad2 because Mad2 no longer can bind with the Mad2-Binding motif (S5 and S6 Tables) [21]. Tyc1 also inhibited APC/C^Cdc20-106^ when present in excess (S7, Fig 6).

### Tyc1 binds APC/C and blocks the interaction between a Cdc20 Mad2-binding motif peptide with the APC/C

**Fig 12:**
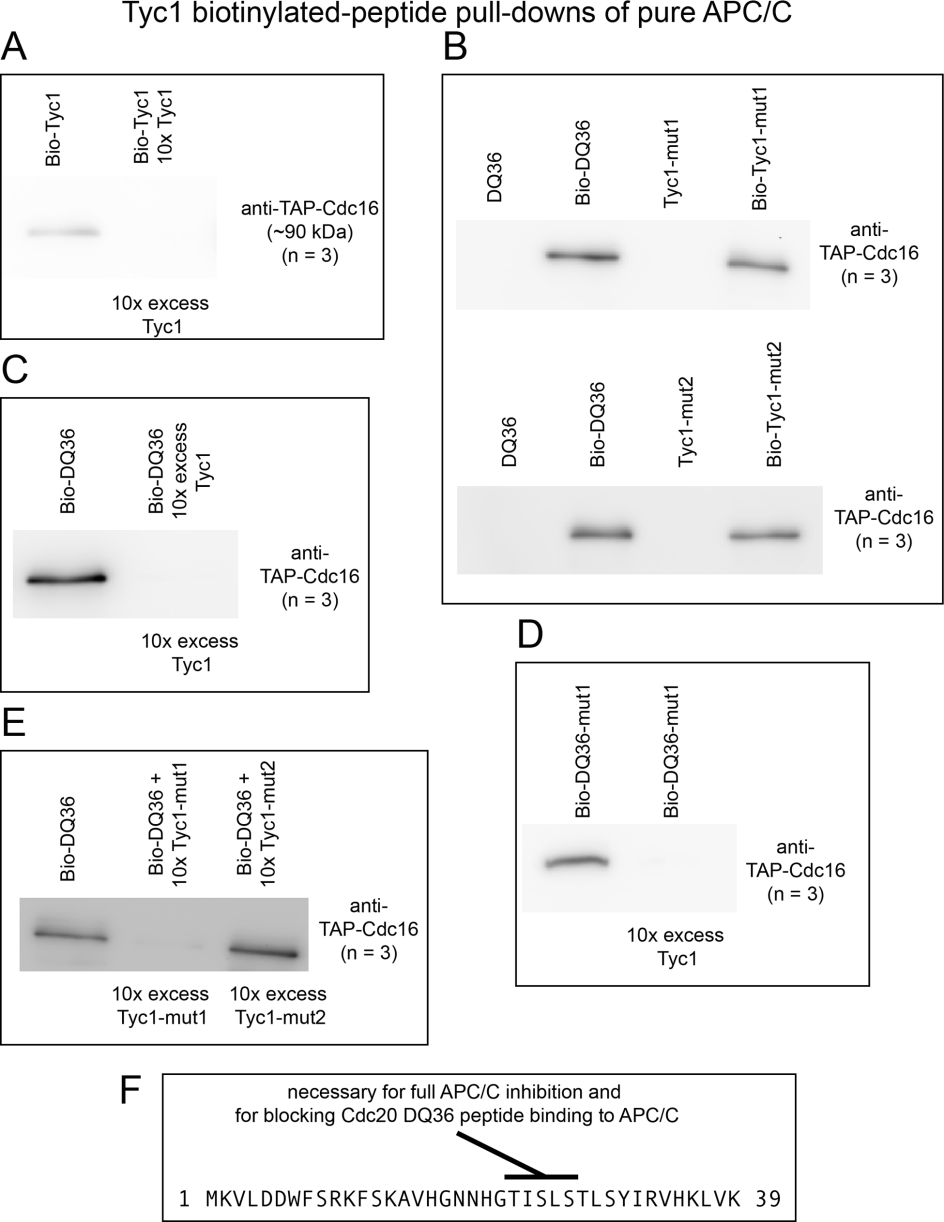
Tyc1 binds APC/C and blocks the ability of the Mad2 binding motif peptide DQ36 to bind to APC/C. A) Biotinylated Tyc1 pull-downs of APC/C. The addition of an excess of non-biotinylated Tyc1 peptide was able to block the pull-down activity. B) Mutant Tyc1-mut1 and Tyc1-mut2 maintained the ability to pull-down APC/C (n = 3). C) An excess of Tyc1 blocked the ability of Mad2 binding motif peptide DQ36 from binding with the APC/C (n = 3). D) An excess of Tyc1 blocked the ability of Mad2 binding motif peptide DQ36-mut1 from binding with the APC/C (n = 3). E) An excess of Tyc1-mut1 retained the ability to block DQ36 peptide binding with APC/C. An excess of Tyc1-mut2 lost the ability to block DQ36 peptide binding with APC/C (n = 3). F) A summary of the activities of Tyc1 highlighting that the conserved TISLS residues are necessary for full APC/C inhibition and for blocking the binding between the Mad2 binding motif peptide DQ36 with the APC/C.

To determine if Tyc1 could interact directly with the APC/C a pure N-terminal biotinylated form of Tyc1 was incubated with purified APC/C. Tyc1 was observed to co-precipitate APC/C as detected by Western blots, where an excess of non-biotinylated Tyc1 was observed to compete and block the interaction (Fig 12A). Thus, like Mad2-binding motif peptides, unexpectedly Tyc1 protein binds to and inhibits APC/C when present in excess leading to a microtubule poison sensitivity phenotype *in vivo*. Both Tyc1-mut1 and Tyc1-mut2 were also observed to co-precipitate APC/C (Fig 12B) revealing that these conserved residues are not necessary for interacting with the APC/C. Tyc1 also pulled-down a full-length form of *in vitro* transcribed/translated Cdc20 from within a rabbit reticulocyte lysate, that may occur via a direct or indirect interaction (S8 Fig). Tyc1-mut2, where the highly conserved TISLS residues were changed to AAAAA, displayed a weaker ability to pull-down Cdc20 from the IVT/T lysate indicating these conserved residues may make a contribution to this interaction (S8 Fig).

Because we observed the ability of Tyc1 to pull-down a full-length form of Cdc20 *in vitro*, we tested if Tyc1 might affect the ability of a Cdc20 Mad2-binding motif peptide to bind with the APC/C. We added in an excess of Tyc1 to the biotinylated-DQ36 APC/C binding reaction and observed that Tyc1 blocks this interaction (Fig 12C). Tyc1 also blocked the ability of DQ36-mut1 to bind with APC/C (Fig 12D), indicating the yeast KILR-motif residues are not required for the observed ability of Tyc1 to block the binding activity of DQ36 with the APC/C. However, the ability of Tyc1 to block the Mad2-binding motif peptide DQ36 binding to APC/C did require the highly conserved TISLS residues, as Tyc1-mut2 lost the ability to block peptide binding (Fig 12E). This result correlates with observed decrease in the ability of Tyc1-mut2 to inhibit APC/C^Cdc20^ (see Fig 11B) and an observed decrease in the ability of Bio-Tyc1-mut2 to pull-down full-length Cdc20 from IVT/T extracts (see S8 Fig) (Fig 12F).

### A peptide derived from human p31^comet^ can bind to and inhibit budding yeast APC/C

**Fig 13:**
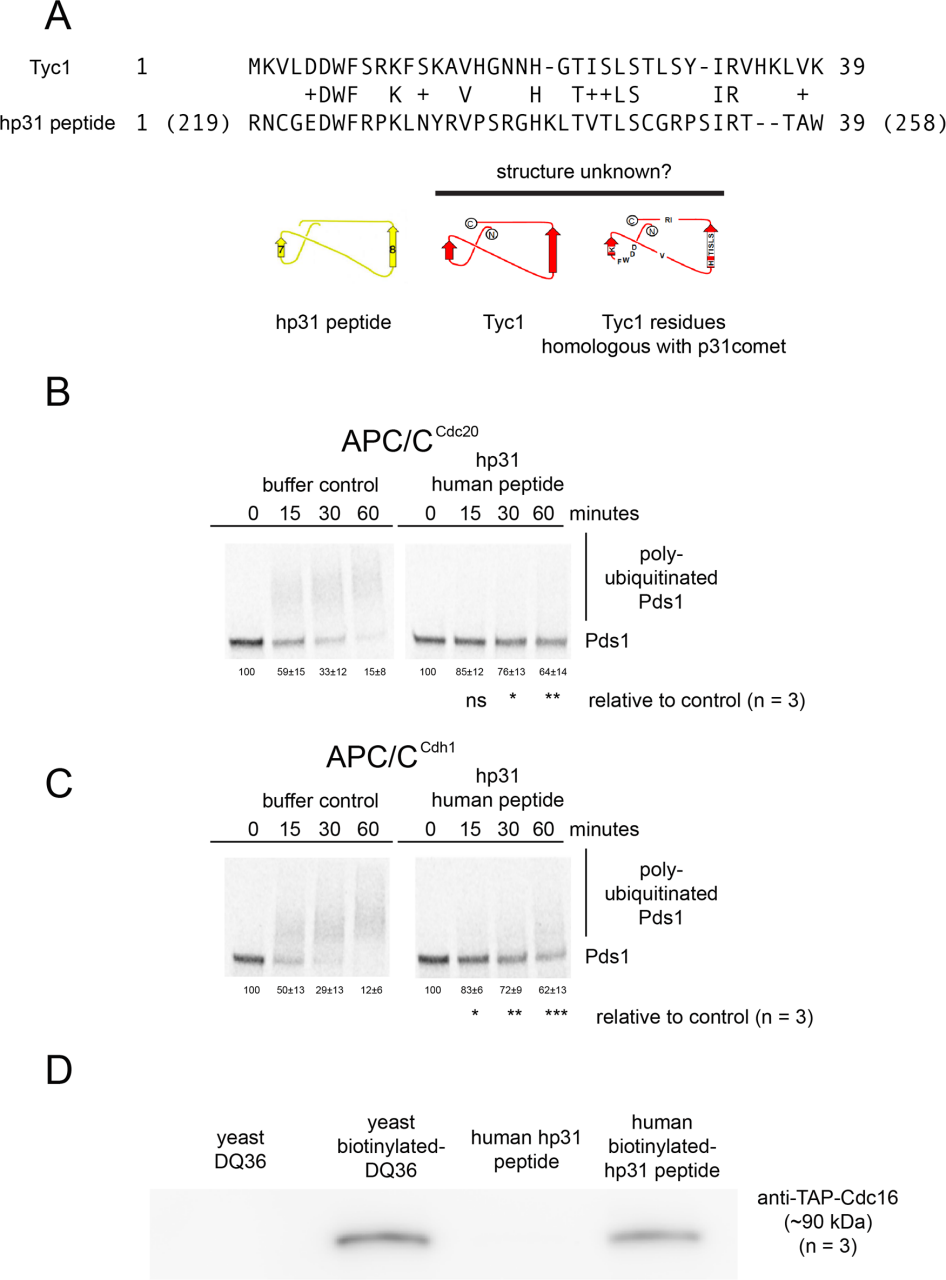
The human peptide hp31 inhibits budding yeast APC/C activity and binds to yeast APC/C. A) An alignment between budding yeast Tyc1 and the synthesized human hp31 peptide derived from human p31^comet^. B) Phosphor-images show that 220 μM human hp31 peptide inhibits yeast APC/C^Cdc20^. C) Phosphor-images show that 220 μM human hp31 peptide inhibits yeast APC/C^Cdh1^. D) The human-derived hp31 can pull-down budding yeast APC/C at 20 μM peptide (n = 3).

Tyc1 was identified based on homology with human p31^comet^, but based on the unexpected binding and inhibition of APC/C by Tyc1 we decided to investigate if the homologous region from human p31^comet^ might also be able to perform these biochemical functions. The region of homology between human p31^comet^ and Tyc1 is small, only 39 amino acids, whereas p31^comet^ is a 274 amino acid residue protein. To determine if the same small homologous region of human p31^comet^ might also contain an ability to bind and inhibit APC/C, we tested if a synthesized 39 amino acid residue human peptide derived from human p31^comet^ amino acid residues 219–258 could inhibit budding yeast APC/C (Fig 13A). The hp31 peptide inhibited both budding yeast APC/C^Cdc20^ (Fig 13B) and APC/C^Cdh1^ (Fig 13C). An N-terminal biotinylated form of the human peptide hp31 also bound to budding yeast APC/C directly (Fig 13D).

### Cdc20 Mad2-binding motif peptides and Tyc1 disrupted the ability of Cdc20 and Cdh1 co-factors to bind to APC/C

**Fig 14:**
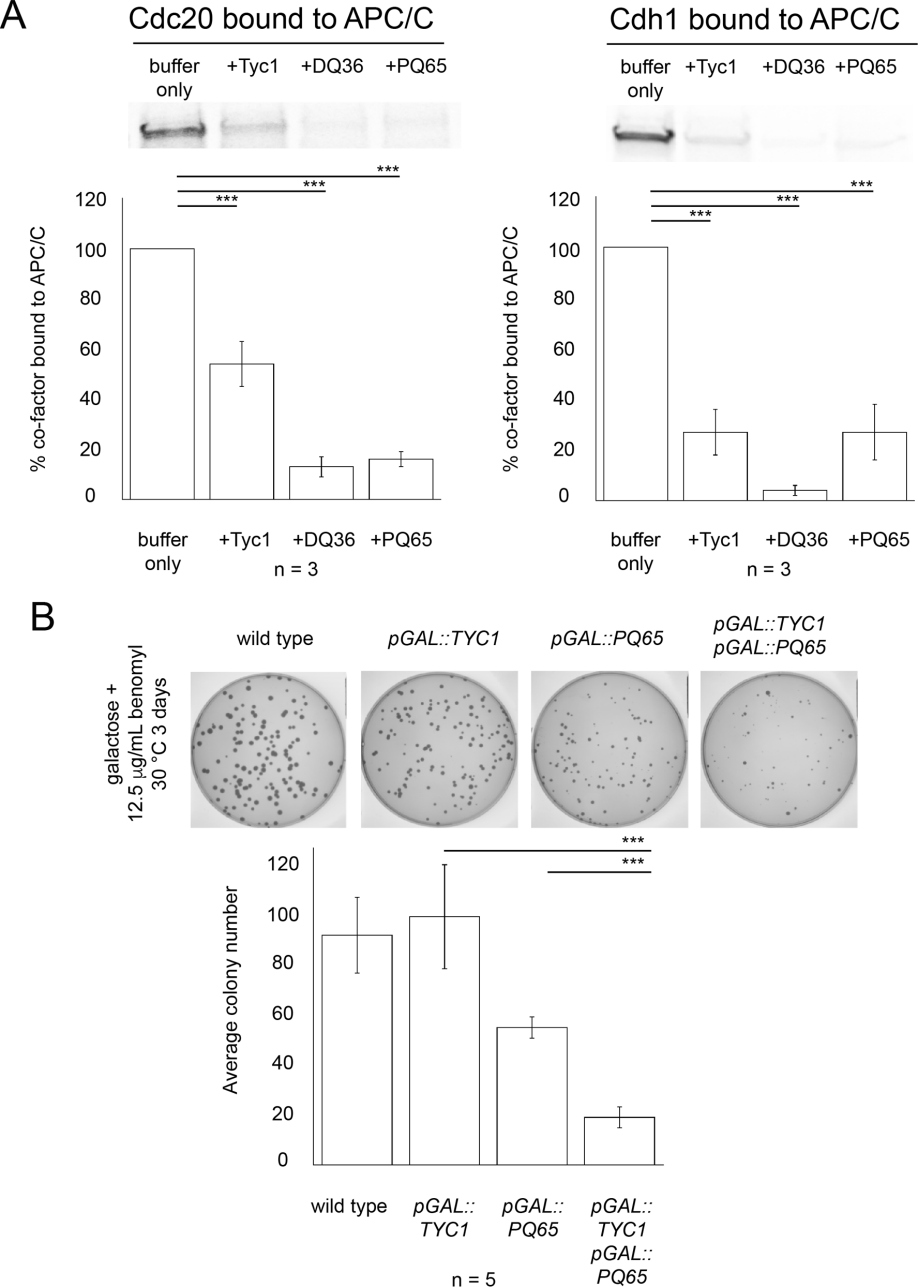
Mad2-binding motif peptides and Tyc1 disrupt co-factor binding to APC/C and display an additive phenotype for microtubule poison sensitivity when co-ever expressed. A) ^35^S-Methionine radio-labeled full-length Cdc20 or Cdh1 were made by IVT/T and pre-incubated with buffer only as a control, or Tyc1, DQ36 or PQ65 at a final concentration of 220 μM. Samples were mixed with enriched APC/C isolated on IgG beads and allowed to bind for 1 hour. Samples were washed and beads were boiled directly in protein sample buffer. The amount of APC/C-bound co-factor was measured by phosphor-imaging (n = 3). B) Co-over expression of PQ65 with Tyc1 *in vivo* leads to an increased sensitivity to the microtubule poison benomyl. In the presence of 12.5 μg/mL benomyl a significant decrease in the average colony number was observed (n = 5).

One potential mechanism by which Cdc20-derived peptides and Tyc1 inhibit APC/C may be by blocking and/or disrupting the association between the co-factors Cdc20 and Cdh1 and the APC/C holo-enzyme. To test this hypothesis we produced radio-labeled full-length Cdc20 and Cdh1 co-factors by IVT/T and then incubated them with 220 μM Tyc1, DQ36 or PQ65 and APC/C that had been isolated from mitotic yeast extracts on IgG beads. Unbound peptide was washed away, and the amount of the remaining APC/C bound Cdc20 or Cdh1 was quantified. Relative to a peptide buffer only control all three peptides Tyc1, DQ36 or PQ65 caused a significant decrease in the amount of APC/C bound co-factor Cdc20 and Cdh1 (Fig 14A). These observations indicate that Tyc1 and Mad2-binding motif peptides inhibit APC/C via a similar mechanism, leading to the disruption of the co-factor-APC/C interaction. This similar mechanism of action predicts that co-ever expression of Tyc1 and a Mad2-binding motif peptide *in vivo* would likely increase a cell’s sensitivity to microtubule poison. To test this hypothesis, cells co-over expressing Tyc1 and PQ65 were plated on 12 μg/mL benomyl for 3 days and were observed to display a significant loss in the average colony numbers (Fig 14B) indicating that Tyc1 and PQ65 display an additive effect on sensitizing cells to a microtubule poison.

### Discussion

We have observed that Mad2 binding motif peptides derived from Cdc20 and the endogenous 39 amino acid Tyc1 protein, that contains homology to human p31^comet^, can function as APC/C inhibitors when present in excess. Cdc20-derived peptides and Tyc1 also induced sensitivity to a microtubule poison when over-expressed *in vivo*. Over expression of a Mad2-binding motif peptide delayed the degradation of yeast securin Pds1 upon recovery from a benomyl arrest, and both Mad2-binding motif peptides and Tyc1 disrupt Cdc20 and Cdh1 co-factor binding with the APC/C. Notably, we have also observed that a homologous synthesized human peptide derived from human p31^comet^ bound to and inhibited budding yeast APC/C, demonstrating evolutionary retention of the inhibitory activity, even though only 11 out of the 39 amino acid residues are conserved. We conclude that Cdc20-derived peptides, the Tyc1 protein, and the human-derived hp31 peptide can serve as novel molecular tools to explore mechanisms for inhibiting the APC/C that give rise to a sensitivity to microtubule poison *in vivo* as a strategy for moving towards a long-term goal of targeting cancer cells that have been treated with a microtubule poison but under-go ‘mitotic slippage’ [1–12].

The PQ65 (Cdc20 167–232) peptide derived from Cdc20 displayed the highest level of APC/C inhibition *in vitro*. Altering the defined budding yeast KILR-motif residues RILQY to AAAAA gave rise to a loss in inhibitor activity. This suggests that the KILR-motif makes a substantial contribution to the observed inhibition, but that also the regions towards the N- or C-termini of the peptide outside of the KILR-motif also make contributions to inhibition. Indeed, the N-terminal region between the C-box and the Mad2-binding motif has the ability to inhibit APC/C as observed by analyzing PS46 (Cdc20 167–213) and the PL29 (Cdc20 167–196) peptides relative to PQ65 (Cdc20 167–232). PS46 maintained the ability to inhibit the APC/C compared to PQ65, but was consistently observed to pull-down less APC/C indicating that the 19 amino acids in the C-terminal region of PQ65 make a contribution to peptide binding with the APC/C. From structural analysis, this 19 amino acid region was undefined, and which APC/C subunit it interacts with remains unknown [31, 48]. PL29 does not contain any residues from the defined Mad2-binding motif or KILR-motif and only contains a few potentially highly conserved amino acids between yeast and humans and was derived from a region of unknown function in between the C-box and Mad2-binding motif, which is not well studied. In the context of the full-length Cdc20, the PL29 residues have been observed to associate closely with the Apc1 subunit of the holo-enzyme complex [48]. The inhibitory target site of PL29 (Cdc20 167–196) remains unknown, but in future work we plan to test the hypothesis that PL29 may be targeting a region on Apc1, where Apc1 is a regulated and an important subunit for APC/C activity [48].

The shorter DQ36 (Cdc20 197–232) peptide containing the Mad2-binding motif and the C-terminal region with several highly conserved residues had the ability to inhibit APC/C at a significant level, although the measured IC_50_ was about 3-times weaker than that of PQ65 (Cdc20 167–232). In the context of DQ36, the yeast KILR-motif RILQY was essential for inhibition, but was not required for binding to the APC/C as observed by analyzing DQ36-mut1. This indicates that for DQ36 inhibition of APC/C that binding to the APC/C is not equivalent to inhibition. The mechanism of inhibition remains unknown, but based on the analysis of DQ36-mut1 we speculate that an interaction between the KILR-motif residues RILQY on the surface of the Cdc23 (APC8B) subunit may be important for the observed inhibition [48]. We hypothesize that this potential interaction between the KILR-motif RILQY and the surface of Cdc23 (APC8B) may promote an allosteric change in protein structure that may be a contributing factor in APC/C inhibition.

In contrast to the analysis of DQ36-mut1, the analysis of DQ36-mut2, SQ19 (Cdc20 213–232) and SQ19-mut2 peptides, and the lack of any observed inhibition by the DS17 (Cdc20 197–212) peptide, all indicate that the 19 amino acid C-terminal region down stream of the KILR-motif makes a contribution to inhibition, where it appears there is a correlation between the strength of inhibition and the strength of the observed binding for this region. DQ36-mut2 has a diminished level of inhibition and consistently displayed weaker APC/C binding. The C-terminal SQ19 peptide still retained some ability to inhibit the APC/C, although it was weaker. However, SQ19-mut2 lost both inhibitory activity and the ability to bind with APC/C. As expected, the short DS17 peptide that lacked this C-terminal region all together did not have the ability to inhibit APC/C, even though it contained the full KILR-motif. In this initial set of experiments, we designed the mutant forms of the peptides in the C-terminal 19 amino acids by focusing on and changing the amino acid residues that displayed the highest level of conservation within fungi. Going forward, we intend to perform an analysis on the 6 amino acid residues that are conserved between yeast and humans, where three of these amino acid residues over-lap at Y220, R225, and Y228 with those that we have started to investigate in this study.

To validate our *in vitro* observations we tested if over expression of the PQ65 or DQ36 peptides *in vivo* could induce a microtubule poison sensitivity phenotype. For the individual over expression of peptides, we observed a significant phenotype. The phenotype is completely reversed simply by changing the KILR-motif residues RILQY to AAAAA providing a strong correlation between the loss of inhibitory activity *in vitro* with the reversion of the benomyl microtubule poison sensitivity phenotype *in vivo*. We went on to observe a delay in Pds1 degradation in cells over expressing DQ36 as they recovered from a microtubule poison-induced cell cycle arrest, an observation that corroborates the *in vitro* data that an excess of a Mad2-binding motif peptide can function to inhibit APC/C.

Because over expression of DQ36 led to delayed degradation of Pds1, were curious to try and identify a potential homologue of p31^Comet^ in yeast, a known factor for promoting cell cycle recovery after a mitotic arrest. We identified Tyc1 as a potential homologue with human p31^comet^ [33–42]. Tyc1 only contains a very short fragment of amino acid sequence of homology with p31^comet^, where the homology is within the Mad2-like “safety-belt” loop that in closed-Mad2 targets the Mad2-binding motif of Cdc20 [36]. The short region of homology implies that Tyc1 functions in a way that is fundamentally different from the proposed mechanism for human p31^comet^.

The *TYC1* gene is non-essential, but induced sensitivity to benomyl when over expressed that was similar to the phenotype observed for the Cdc20 peptides when over expressed. The function of Tyc1 *in vivo* remains unclear, but inducing sensitivity to benomyl, which is increased in the absence of the spindle checkpoint protein *mad2Δ*, indicates that Tyc1 is likely to perform a mitotic function. We intend to continue to investigate the function of native Tyc1 *in vivo* by performing further genetic and molecular genetic functional analyses.

In addition to Tyc1 inducing a similar phenotype to the Mad2-binding motif peptides when over expressed *in vivo*, a second unexpected similarity was observed *in vitro*, as Tyc1 was also able to inhibit and bind to the APC/C. The full inhibition of APC/C^Cdc20^ required the highly conserved TISLS residues in the C-terminal portion of the protein. Tyc1 bound to APC/C directly, and could also pull-down a full-length form of Cdc20 made by *in vitro* transcription/translation. Notably, Tyc1 also had the ability to block the DQ36 peptide from binding with the APC/C, an activity, which also depended on the conserved TISLS residues. This result correlates with observed decrease in the ability to inhibit APC/C^Cdc20^ and an observed decrease in the ability of Bio-Tyc1-mut2 to pull-down full-length Cdc20 from IVT/T extracts indicating these conserved TISLS residues may be important for Tyc1 function.

Tyc1 was identified based on homology with the human p31^comet^ protein. However, our results indicate that Tyc1 functions in a way that is different from the way that is currently proposed for human p31^comet^, which releases Cdc20 from inhibition by Mad2, and promotes mitotic progression [33–42]. Because of this unexpected difference, we examined if the homologous region of human p31^comet^ retained the ability to inhibit budding yeast APC/C. We have observed that the human 39 amino acid region, which only contains 11 conserved amino acid residues, in the form of the hp31 human peptide was able to bind to and inhibit budding yeast APC/C. This indicates that this inhibitory function was retained during the course of evolution between yeast and humans.

One simple potential mechanism of APC/C inhibition might be a disruption in the ability of the co-factors Cdc20 and Cdh1 to interact with the APC/C holo-enzyme. We tested this hypothesis and observed that Tyc1, DQ36 and PQ65 displayed the ability to disrupt both Cdc20 and Cdh1 from interacting with the APC/C *in vitro*. This observation provides a common potential mechanism of inhibition for these different peptides, and also indicated that co-over expression of the peptides in the same cell would increase the microtubule poison phenotype, where an additive phenotypic effect was observed.

### Conclusion

In the broader context, there is a general interest in trying to identify and develop inhibitors of APC/C as potential therapeutic agents targeting cells that undergo ‘mitotic slippage’ even after being exposed to microtubule poisons [1–12]. Our current set of APC/C inhibitor peptides are too big and are functionally too weak to serve as potential therapeutic agents. To make progress, we are confident that Cdc20-derived peptides, the Tyc1 protein, and the human-derived hp31 peptide can all serve as novel molecular tools to further explore mechanisms for inhibiting the APC/C that give rise to a sensitivity to microtubule poison *in vivo* as a strategy for targeting cancer cells. Our current work is developing in three directions: i) we are actively investigating unstudied regions in the peptides and their potential APC/C target sites by focusing on conserved amino acid residues between yeast and humans, ii) we have designed a set of peptide-mimetic small molecules using D-amino acids and will employ budding yeast as a model test system to explore their ability to inhibit the APC/C, and iii) we have begun to employ a human cell-based APC/C assay to test if homologous human Cdc20 peptides and the human hp31 peptide can function as inhibitors of human APC/C.

## Supporting information

S1 Fig Quantitative measurements of yeast APC/C^Cdc20^ inhibition by MCC subunits. A) Phosphor-image of an initial set of APC/C^Cdc20^ reaction time courses in the presence of buffer (left) or pure recombinant MCC inhibitors, such as Mad2 alone (middle-left), the Mad3-Bub3 complex (middle-right), or the Mad3-Bub3 complex plus Mad2 (right). B) The measured IC_50_ for Mad2 was observed to be 2.3 ± 0.5 μM with a Hill coefficient of 2.4 ± 0.3. The graph of the titration curve over two orders of magnitude for Mad2 is shown (top). In each graph, the solid line is the estimated best-fit curve of a 4-parameter Hill equation with the data, and error bars represent the standard deviations from three independent titrations. The three phosphor-images show the increase in substrate consumption as the amount of Mad2 inhibitor is decreased from right to left (bottom). C) Phosphor-image of an initial APC/C^Cdc20^ reaction time courses in the presence of buffer (left) or Bub3 (right). D) The IC_50_ for Bub3 was approximately 8.7 ± 2.4 μM with a Hill coefficient of 2.2 ± 0.4. E) The measured IC_50_ for Mad3 was 240 ± 10 nM with a Hill coefficient of 5.0 ± 0.9. F) The measured IC_50_ for the Mad3-Bub3 complex is 89 ± 4 nM with a Hill coefficient of 7.3 ± 2.6.
(TIF)

S2 Fig Mad2 and the Mad3-Bub3 complex work synergistically to promote full inhibition of APC/C^Cdc20^ substrate poly-ubiquitination. A) The measured IC_50_ for the Mad3-Bub3 complex in the presence of 0.5 μM Mad2 was 70 ± 3 nM with a Hill coefficient of 8.2 ±1.9. Error bars represent the standard deviations from three independent titrations. B) Phosphor-images of time courses of APC/C^Cdc20^ reactions in the absence (buffer) or presence of Mad2, the Mad3-Bub3 complex, or both at concentrations at about 4-times the measured IC_50_ values. C) Intensity plots from the top of the SDS-PAGE gel (left) towards the bottom (right) of the target substrate Pds1 from the 60 minute time-point shown in B) In the presence of Mad2 (red), the small amount of Pds1 that is ubiquitinated appears to be fully poly-ubiquitinated. The Inset shows a magnified view of the mono-, di-, tri-, tetra-, and penta-ubiquitinated species of Pds1 revealing that in the presence of the Mad3-Bub3 complex (green) the Pds1 accumulated in these lower molecular weight species. D) An example of a silver-stained gel of the purified APC/C that was used to perform the experiments shown in the S1 and S2 Figs. APC/C subunits are labeled based on molecular weight and the banding pattern in comparison to the gels published by Passmore *et al*. 2003 [54].
(TIF)

S3 Fig Analysis of conserved and by-pass allele mutants identify amino acid residues necessary for APC/C activation and Mad2-dependent inhibition of Cdc20. A) Phosphor-images of APC/C reaction time courses in the presence of Cdc20 (left) or Cdc20-127 (Y205N) (right). B) The amount of Pds1 remaining was measured as a percentage of the initial value at the 0 time point and graphed as the mean ± standard deviation (n = 3) over time. Data were analyzed by employing the Student’s *t*-test comparing Cdc20 with Cdc20-127 (Y205N) APC/C activity, which yielded *p*-values of 0.017, 0.008, and 0.011 at 15, 30, and 60 minutes, respectively. C) Phosphor-images of APC/C^Cdc20-127^ reaction time courses in the presence of buffer (left) or Mad2 (right). After IVT/T for 1 hr, Cdc20-127 (Y205N) was incubated with QAH buffer or Mad2 for an additional 1 hr. D) The amount of Pds1 remaining was measured as a percentage of the initial value at the 0 time point and graphed as the mean ± standard deviation (n = 3) over time. Data were analyzed by employing the Student’s *t*-test comparing Cdc20-127 (Y205N) APC/C activity in the absence and presence of Mad2, which yielded *p*-values of 0.290, 0.367, and 0.191 at 15, 30, and 60 minutes, respectively. E) Typical examples of the amounts of enriched APC/C used in enzyme assays above, or for the binding assays that are shown here observed by Coomassie stain. The IgG beads used for protein purification were boiled in protein sample buffer directly, also releasing the IgG heavy (hc) and light chains (lc). The high molecular weight APC/C subunits are readily visible in the amounts or protein isolated on beads from about 1 or 2 mL of a typical yeast extract. F) A summary of the observed results on the analysis of by-pass alleles. Leucine 203 and Tyrosine 205 make a contribution to APC/C activation, and Tyrosine 205 and Proline 209 are required for Mad2 inhibition.
(TIF)

S4 Fig Biotinylated PL29 (Cdc20 167–196) can no longer bind to Avidin in the presence of pure APC/C. A) Coomassie staining of synthesized PL29 (Cdc20 167–196) and biotinylated PL29 (Bio-PL29) peptides. B) Western blot of synthesized peptides alone (left) or of peptides that have been isolated with Avidin magnetic beads (right) as detected by Avidin-HRP. In the absence of the APC/C the Bio-PL29 peptide interacts with the Avidin beads and can be pulled down. C) Bio-PL29 did not bind to Avidin beads in the presence of pure APC/C. Pull-downs of pure APC/C by Bio-DQ36 as a positive control (left) are shown. Both the APC/C and the biotinylated peptide were observed by Western blotting (n = 2). In the presence of pure APC/C Bio-PL29 is no longer able to bind Avidin and the APC/C was not pulled-down by the Avidin beads.
(TIF)

S5 Fig An example of all 5 replicates of the DQ36/DQ36-mut1 and PQ65/PQ65-mut1 peptide over expression phenotypes. A) Five replicates of *pGAL*::*PQ65* and *pGAL*::*PQ65-mut1* when over expressed on control plates without the addition of benomyl. B) Five replicates of *pGAL*::*DQ36* and *pGAL*::*DQ36-mut1* when over expressed on control plates without the addition of benomyl. C) Quantitation of the results shown in A and B demonstrates there is no significant difference in colony numbers per plate in the absence of benomyl, even in strains over expression the peptides. D) Five replicates of *pGAL*::*PQ65* and *pGAL*::*PQ65-mut1* when over expressed on experimental plates containing 12.5 μg/mL of benomyl where quantitative results are displayed in Fig 7 in the text. E) Five replicates of *pGAL*::*DQ36* and *pGAL*::*DQ36-mut1* when over expressed on experimental plates containing 12.5 μg/mL of benomyl where quantitative results are displayed in Fig 7 in the text.
(TIF)

S6 Fig Tyc1 inhibits APC/C^Cdc20^ and APC/C^Cdh1^ when Cdc20 is used as the target substrate. A) APC/C^Cdc20^ reactions using Pds1 (top) and Cdc20 (bottom) as the target substrates. Tyc1 inhibits APC/C^Cdc20^ activity in both reactions, but the quantification of the Cdc20 substrate results is confounded by the presence of lower molecular weight forms that also migrate up the gel over time. B) APC/C^Cdh1^ reactions using Pds1 (top) and Cdc20 (bottom) as the target substrates. Tyc1 inhibits APC/C^Cdh1^ activity in both reactions, but the quantification of the Cdc20 substrate results is confounded by the presence of lower molecular weight forms that also migrate up the gel over time.
(TIF)

S7 Fig Tyc1 inhibits APC/C^Cdc20-106^. A) APC/C^Cdc20^ reactions using the wild type Cdc20 are shown as a positive control. B) APC/C^Cdc20-106^ reactions are also inhibited by Tyc1. The Cdc20-106 (P209Q) allele was chosen because this allele can promote a normal level of APC/C activity but was not inhibited by Mad2 (see S5 and S6 Tables).
(TIF)

S8 Fig Tyc1 can pull-down recombinant Cdc20 synthesized by IVT/T. A) A Western blot displaying the detection of Cdc20 protein made by IVT/T (left), where the epitope tag is at the N-terminus of the ORF. The IVT/T reaction yields a series of protein products that display full-length and lower molecular weights, potentially the result of pre-mature translation termination. An Avidin-bead pull-down using a biotinylated-Tyc1 (Bio-Tyc1) is shown (right). Both full-length and lower molecular weight forms of Cdc20 were observed that all contain the N-terminal region of Cdc20 where the TAP-epitope tag is located. B) An Avidin-bead pull-down using a biotinylated-Tyc1-mut1 (Bio-Tyc1-mut1) (middle) and biotinylated-Tyc1-mut2 (Bio-Tyc1-mut2) are shown (right). We consistently observed (n = 3) that the amount of Cdc20 protein isolated on the Avidin-beads in the presence of Bio-Tyc1-mut2 was lower.
(TIF)

S9 Fig The full unaltered and uncropped images of the gel and blot images used to make figures. Image files and notebook scans are provided in sequential order as they appear in the paper.
(PDF)

S1 Table The estimated median inhibitory concentrations (IC_50_) and Hill coefficients for mitotic checkpoint complex subunits. Standard deviations (sd) were derived from curve fitting a 4-parameter Hill equation to the titration data for each subunit or complex.
(DOC)

S2 Table A comparison of median inhibitory concentrations (IC_50_) values ± standard deviations (sd) for mitotic checkpoint complex subunits using the *t*-test, where *p*-values are shown. (DOC)

S3 Table A comparison of estimated Hill coefficients ± standard deviations (sd) for mitotic checkpoint complex subunits using the *t*-test, where *p*-values are shown. (DOC)

S4 Table A comparison of mutant versus wild type Cdc20 protein production by IVT/T employing the Student’s *t*-test, where *p*-values are shown. ^35^S-labeled protein amounts were measured after 2 hours of protein synthesis via IVT/T. The *cdc20-120* mutant allele contained a single amino acid change at P210, where as the isolated genetic by-pass allele had additional mutations leading the changes at residues T226S and T247I. We chose to focus on the single P210S residue change to simplify the functional analysis of this individual residue, and because it lies within the defined Mad2-binding motif between amino acids 197–223, whereas the T226S change is relatively conservative and lies outside of the defined motif. To mark this difference, we denote this single change amino acid residue allele as cdc20-120*.
(DOC)

S5 Table A comparison of mutant versus wild type Cdc20 APC activity, as measured by Pds1 remaining, employing the Student’s *t*-test, where *p*-values are shown. Time points of 15, 30, and 60 minutes are across the top, and each mutant allele tested is on the left. Boxes shaded in grey indicate a *p*-value < 0.05 relative to wild type Cdc20. The mutant alleles Cdc20-127 (Y205N), Cdc20-L203A, and the control mutant Cdc20-CB (I147A, P148A) all consistently displayed a decrease in APC activity (n = 3).
(DOC)

S6 Table A comparison of mutant versus wild type APC^Cdc20^ activity, as measured by Pds1 remaining, inhibited by 5.0 μM Mad2 employing the Student’s *t*-test, where *p*-values are shown. Time points are on the left, and mutant alleles tested are labeled across the top. Grey boxes indicate a *p*-value > 0.05 relative to wild type Cdc20. The mutant alleles Cdc20-127 (Y205N) and Cdc20-106 (P209Q) consistently displayed APC activity in the presence of Mad2 (n = 3). Cdc20-107 (P210L) was not tested, as the mutation in this residue is at the same amino acid residue as the Cdc20-120* (P210S) mutant allele.
(DOC)
