# Supplementary figures and images for: Peptide inhibitors of the anaphase promoting-complex that cause sensitivity to microtubule poison
Source: PLoS One. 2018 Jun 8;13(6):e0198930. doi: 10.1371/journal.pone.0198930 (PMC5993284; doi:10.1371/journal.pone.0198930)

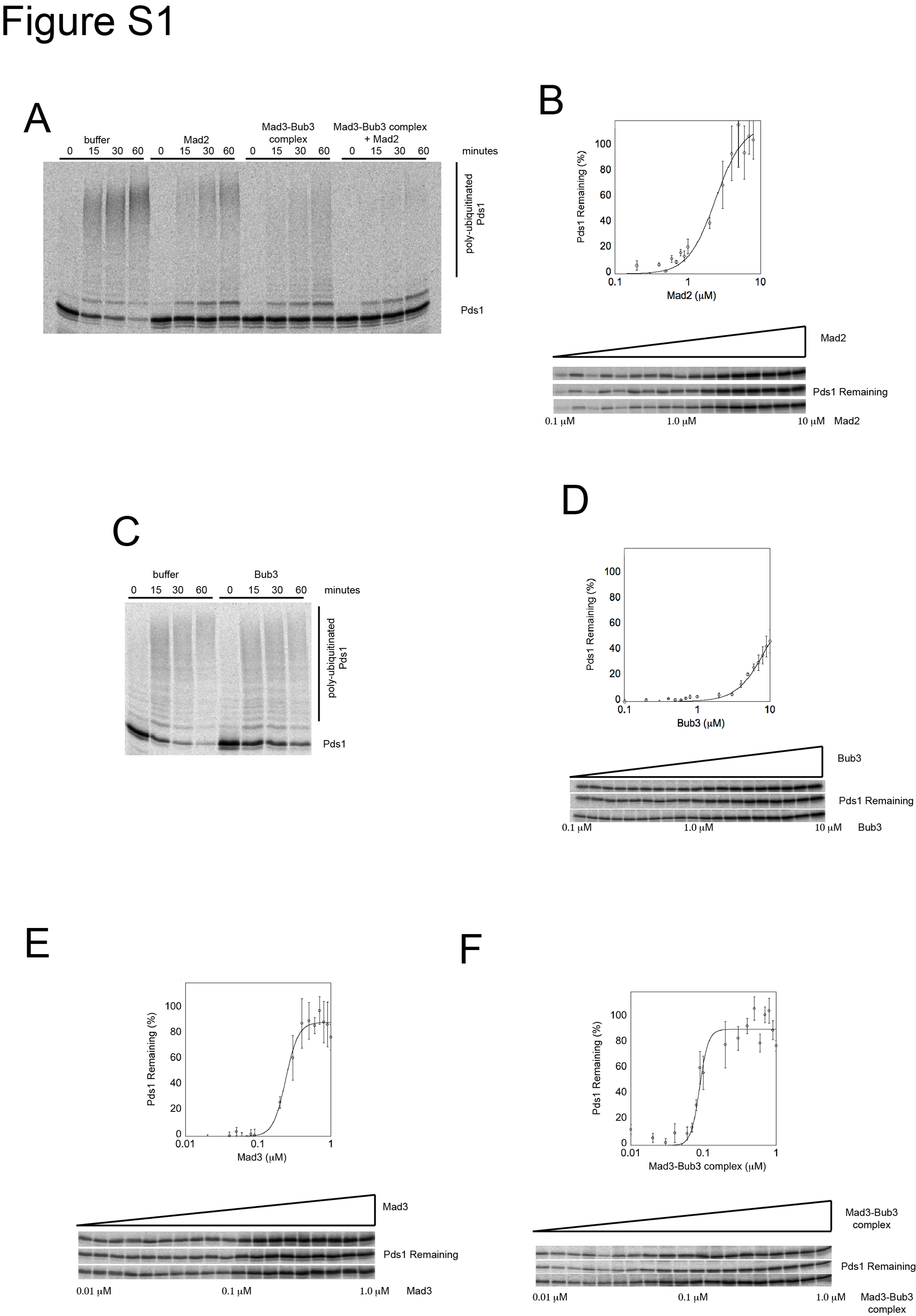

Supplement: S1 Fig — A) Phosphor-image of an initial set of APC/CCdc20 reaction time courses in the presence of buffer (left) or pure recombinant MCC inhibitors, such as Mad2 alone (middle-left), the Mad3-Bub3 complex (middle-right), or the Mad3-Bub3 complex plus Mad2 (right). B) The measured IC50 for Mad2 was observed to be 2.3 ± 0.5 μM with a Hill coefficient of 2.4 ± 0.3. The graph of the titration curve over two orders of magnitude for Mad2 is shown (top). In each graph, the solid line is the estimated best-fit curve of a 4-parameter Hill equation with the data, and error bars represent the standard deviations from three independent titrations. The three phosphor-images show the increase in substrate consumption as the amount of Mad2 inhibitor is decreased from right to left (bottom). C) Phosphor-image of an initial APC/CCdc20 reaction time courses in the presence of buffer (left) or Bub3 (right). D) The IC50 for Bub3 was approximately 8.7 ± 2.4 μM with a Hill coefficient of 2.2 ± 0.4. E) The measured IC50 for Mad3 was 240 ± 10 nM with a Hill coefficient of 5.0 ± 0.9. F) The measured IC50 for the Mad3-Bub3 complex is 89 ± 4 nM with a Hill coefficient of 7.3 ± 2.6. (TIF) [file pone.0198930.s001.tif]

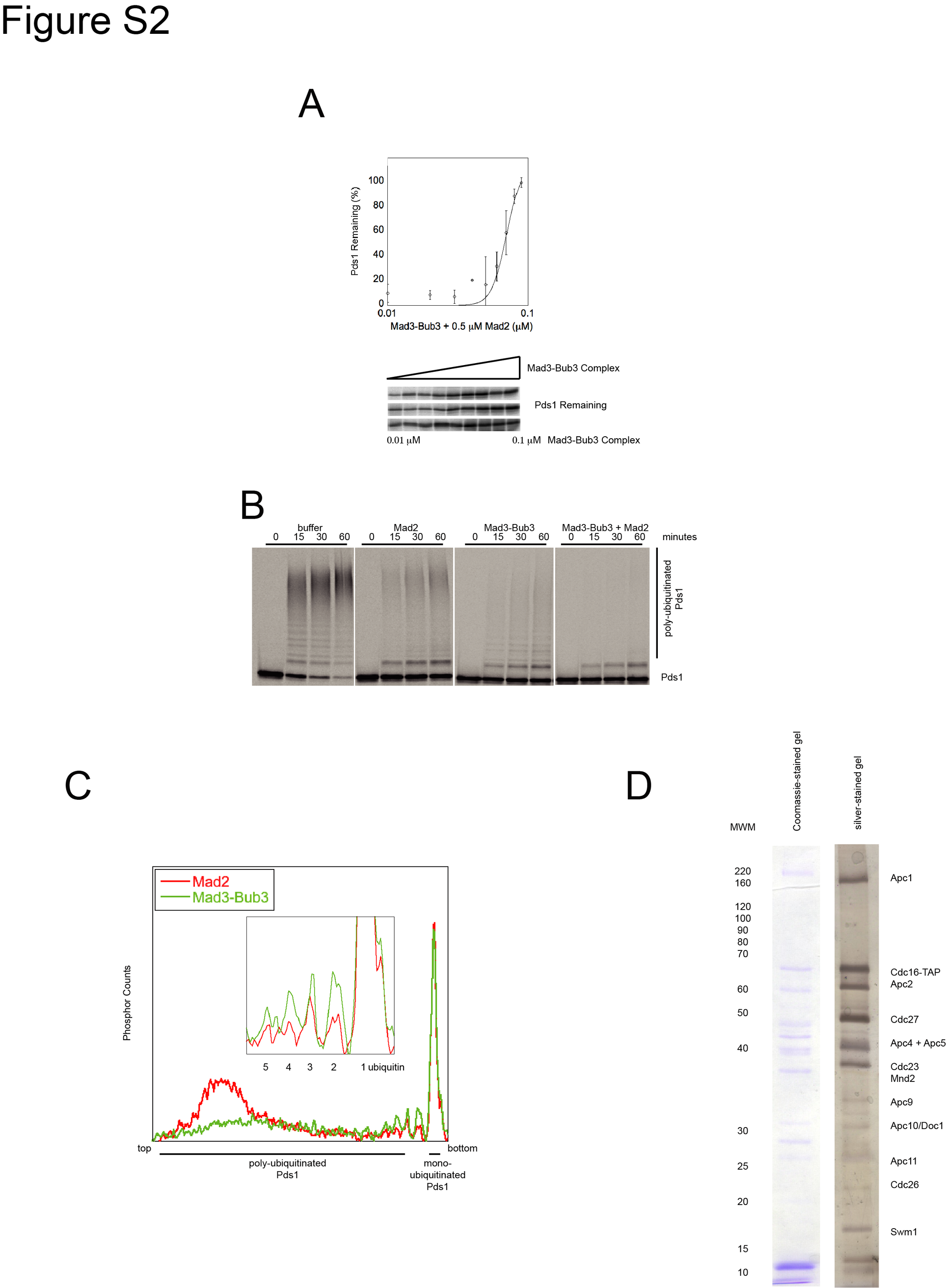

Supplement: S2 Fig — A) The measured IC50 for the Mad3-Bub3 complex in the presence of 0.5 μM Mad2 was 70 ± 3 nM with a Hill coefficient of 8.2 ±1.9. Error bars represent the standard deviations from three independent titrations. B) Phosphor-images of time courses of APC/CCdc20 reactions in the absence (buffer) or presence of Mad2, the Mad3-Bub3 complex, or both at concentrations at about 4-times the measured IC50 values. C) Intensity plots from the top of the SDS-PAGE gel (left) towards the bottom (right) of the target substrate Pds1 from the 60 minute time-point shown in B) In the presence of Mad2 (red), the small amount of Pds1 that is ubiquitinated appears to be fully poly-ubiquitinated. The Inset shows a magnified view of the mono-, di-, tri-, tetra-, and penta-ubiquitinated species of Pds1 revealing that in the presence of the Mad3-Bub3 complex (green) the Pds1 accumulated in these lower molecular weight species. D) An example of a silver-stained gel of the purified APC/C that was used to perform the experiments shown in the S1 and S2 Figs. APC/C subunits are labeled based on molecular weight and the banding pattern in comparison to the gels published by Passmore et al. 2003 [54]. (TIF) [file pone.0198930.s002.tif]

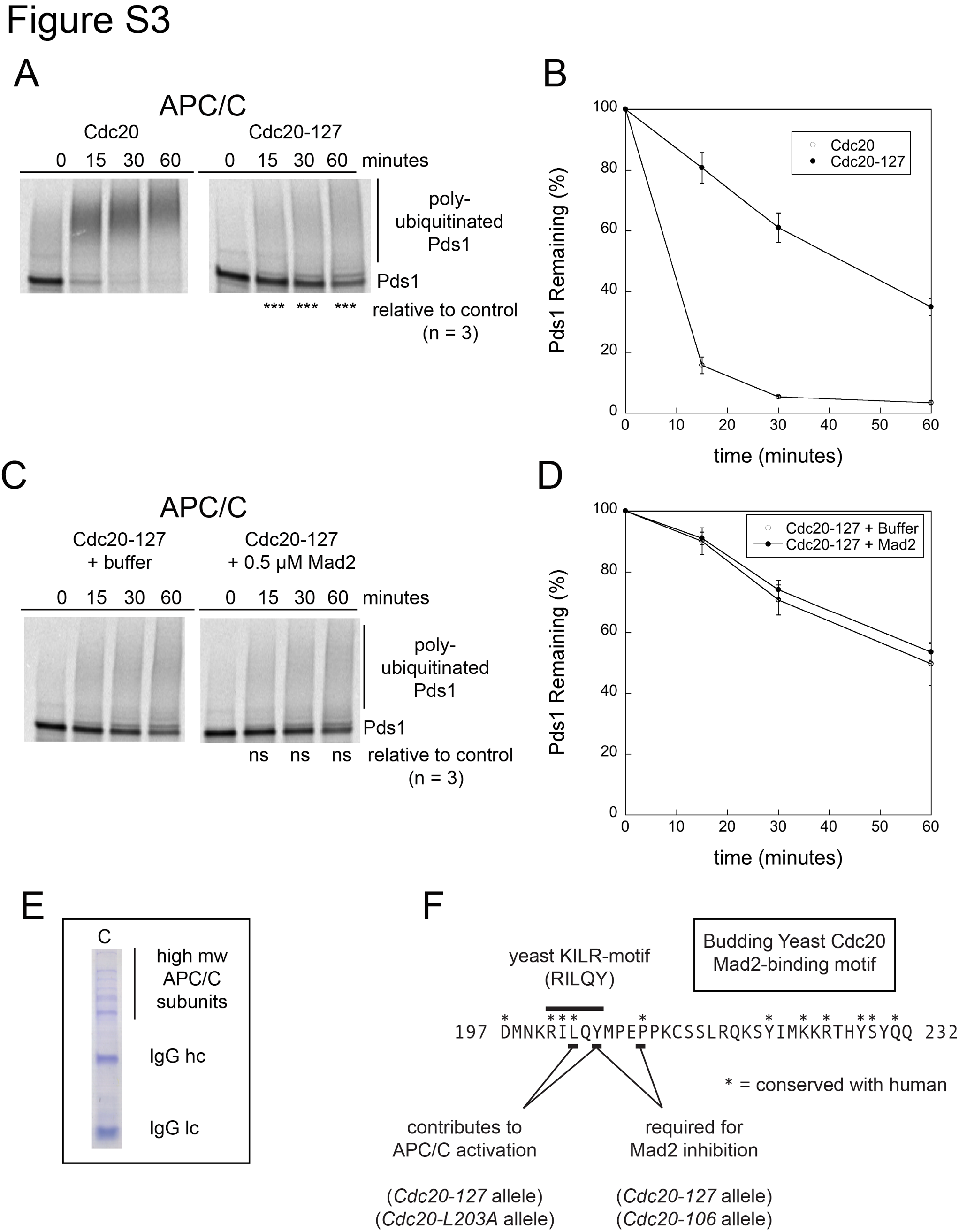

Supplement: S3 Fig — A) Phosphor-images of APC/C reaction time courses in the presence of Cdc20 (left) or Cdc20-127 (Y205N) (right). B) The amount of Pds1 remaining was measured as a percentage of the initial value at the 0 time point and graphed as the mean ± standard deviation (n = 3) over time. Data were analyzed by employing the Student’s t-test comparing Cdc20 with Cdc20-127 (Y205N) APC/C activity, which yielded p-values of 0.017, 0.008, and 0.011 at 15, 30, and 60 minutes, respectively. C) Phosphor-images of APC/CCdc20-127 reaction time courses in the presence of buffer (left) or Mad2 (right). After IVT/T for 1 hr, Cdc20-127 (Y205N) was incubated with QAH buffer or Mad2 for an additional 1 hr. D) The amount of Pds1 remaining was measured as a percentage of the initial value at the 0 time point and graphed as the mean ± standard deviation (n = 3) over time. Data were analyzed by employing the Student’s t-test comparing Cdc20-127 (Y205N) APC/C activity in the absence and presence of Mad2, which yielded p-values of 0.290, 0.367, and 0.191 at 15, 30, and 60 minutes, respectively. E) Typical examples of the amounts of enriched APC/C used in enzyme assays above, or for the binding assays that are shown here observed by Coomassie stain. The IgG beads used for protein purification were boiled in protein sample buffer directly, also releasing the IgG heavy (hc) and light chains (lc). The high molecular weight APC/C subunits are readily visible in the amounts or protein isolated on beads from about 1 or 2 mL of a typical yeast extract. F) A summary of the observed results on the analysis of by-pass alleles. Leucine 203 and Tyrosine 205 make a contribution to APC/C activation, and Tyrosine 205 and Proline 209 are required for Mad2 inhibition. (TIF) [file pone.0198930.s003.tif]

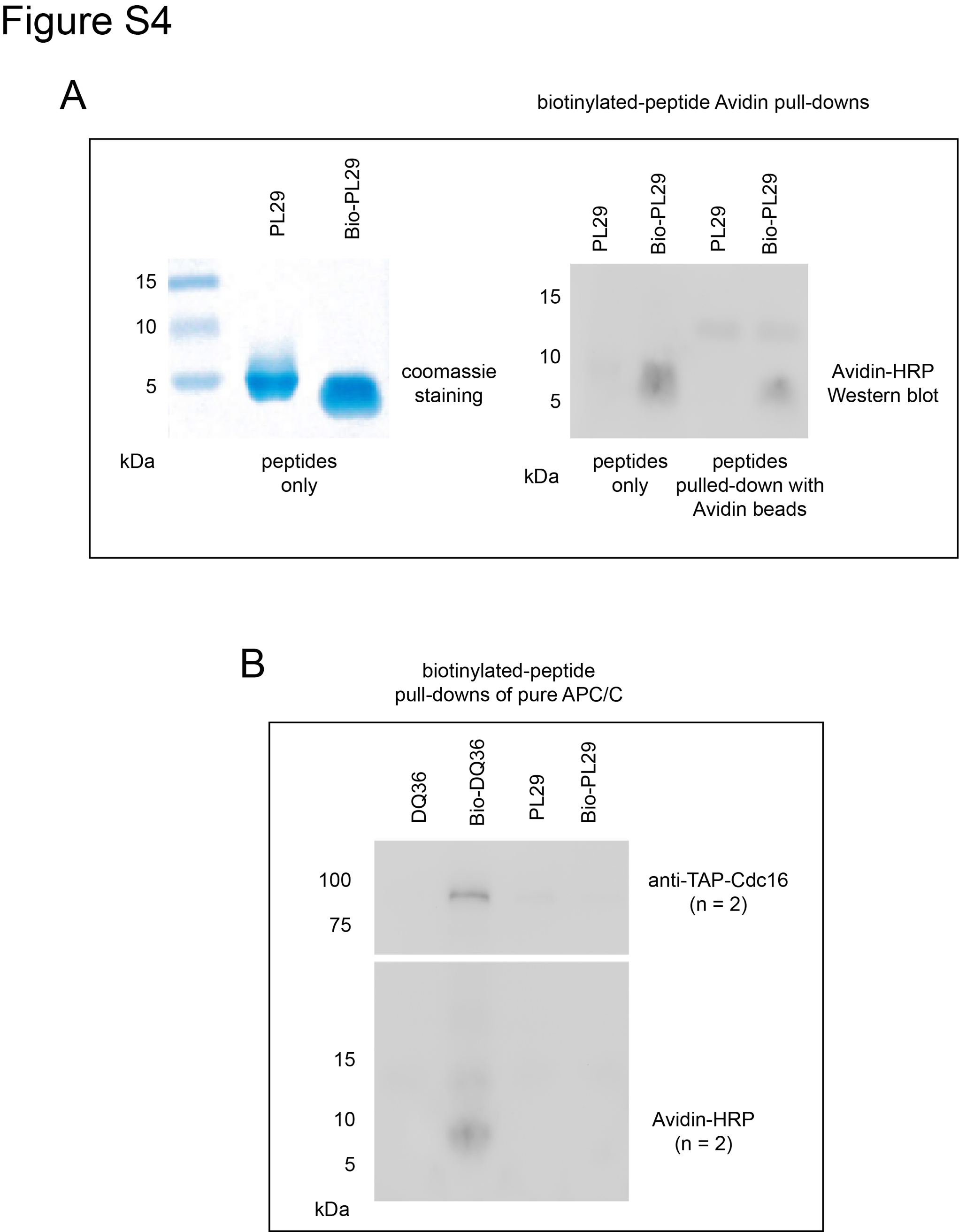

Supplement: S4 Fig — A) Coomassie staining of synthesized PL29 (Cdc20 167–196) and biotinylated PL29 (Bio-PL29) peptides. B) Western blot of synthesized peptides alone (left) or of peptides that have been isolated with Avidin magnetic beads (right) as detected by Avidin-HRP. In the absence of the APC/C the Bio-PL29 peptide interacts with the Avidin beads and can be pulled down. C) Bio-PL29 did not bind to Avidin beads in the presence of pure APC/C. Pull-downs of pure APC/C by Bio-DQ36 as a positive control (left) are shown. Both the APC/C and the biotinylated peptide were observed by Western blotting (n = 2). In the presence of pure APC/C Bio-PL29 is no longer able to bind Avidin and the APC/C was not pulled-down by the Avidin beads. (TIF) [file pone.0198930.s004.tif]

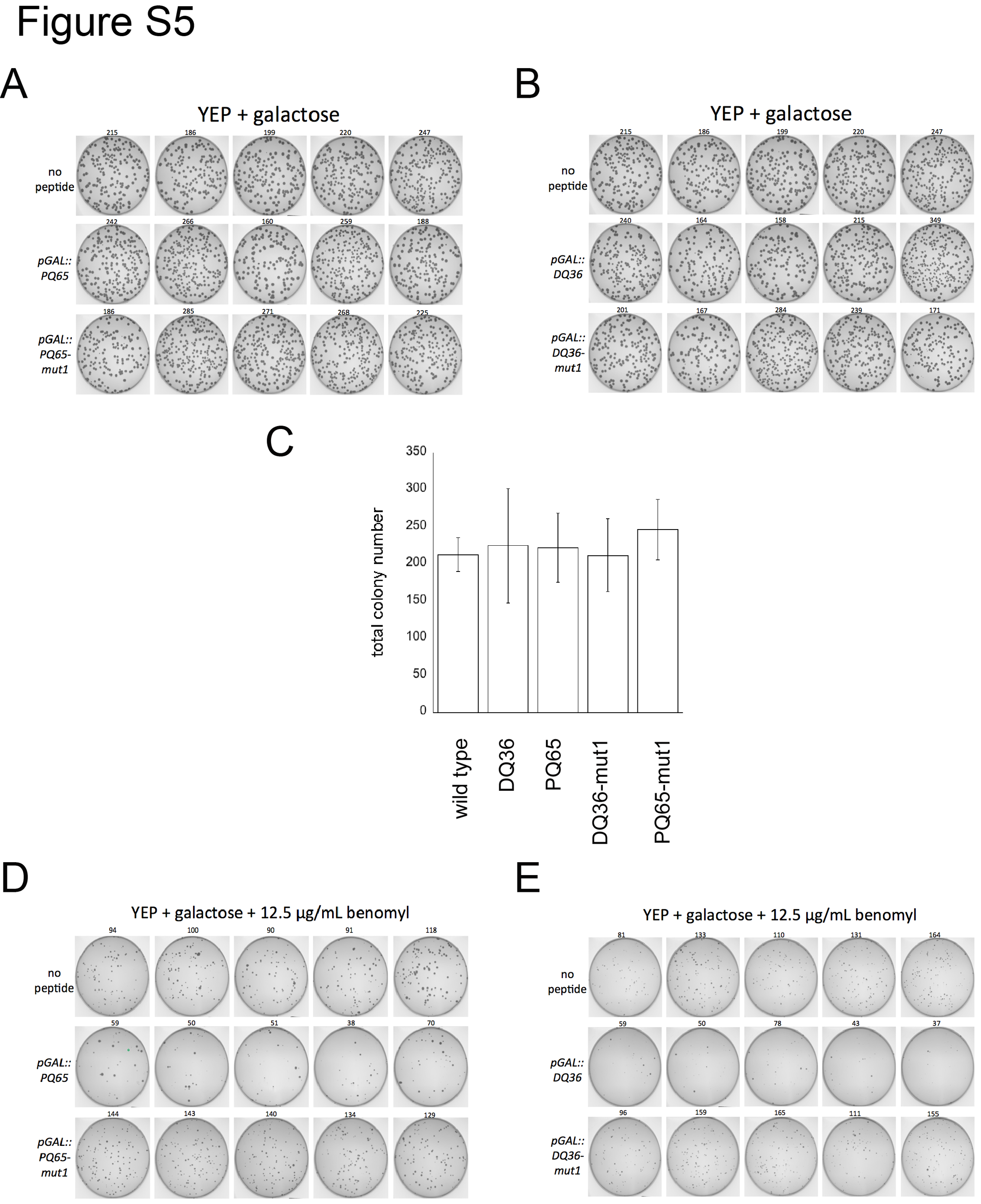

Supplement: S5 Fig — A) Five replicates of pGAL::PQ65 and pGAL::PQ65-mut1 when over expressed on control plates without the addition of benomyl. B) Five replicates of pGAL::DQ36 and pGAL::DQ36-mut1 when over expressed on control plates without the addition of benomyl. C) Quantitation of the results shown in A and B demonstrates there is no significant difference in colony numbers per plate in the absence of benomyl, even in strains over expression the peptides. D) Five replicates of pGAL::PQ65 and pGAL::PQ65-mut1 when over expressed on experimental plates containing 12.5 μg/mL of benomyl where quantitative results are displayed in Fig 7 in the text. E) Five replicates of pGAL::DQ36 and pGAL::DQ36-mut1 when over expressed on experimental plates containing 12.5 μg/mL of benomyl where quantitative results are displayed in Fig 7 in the text. (TIF) [file pone.0198930.s005.tif]

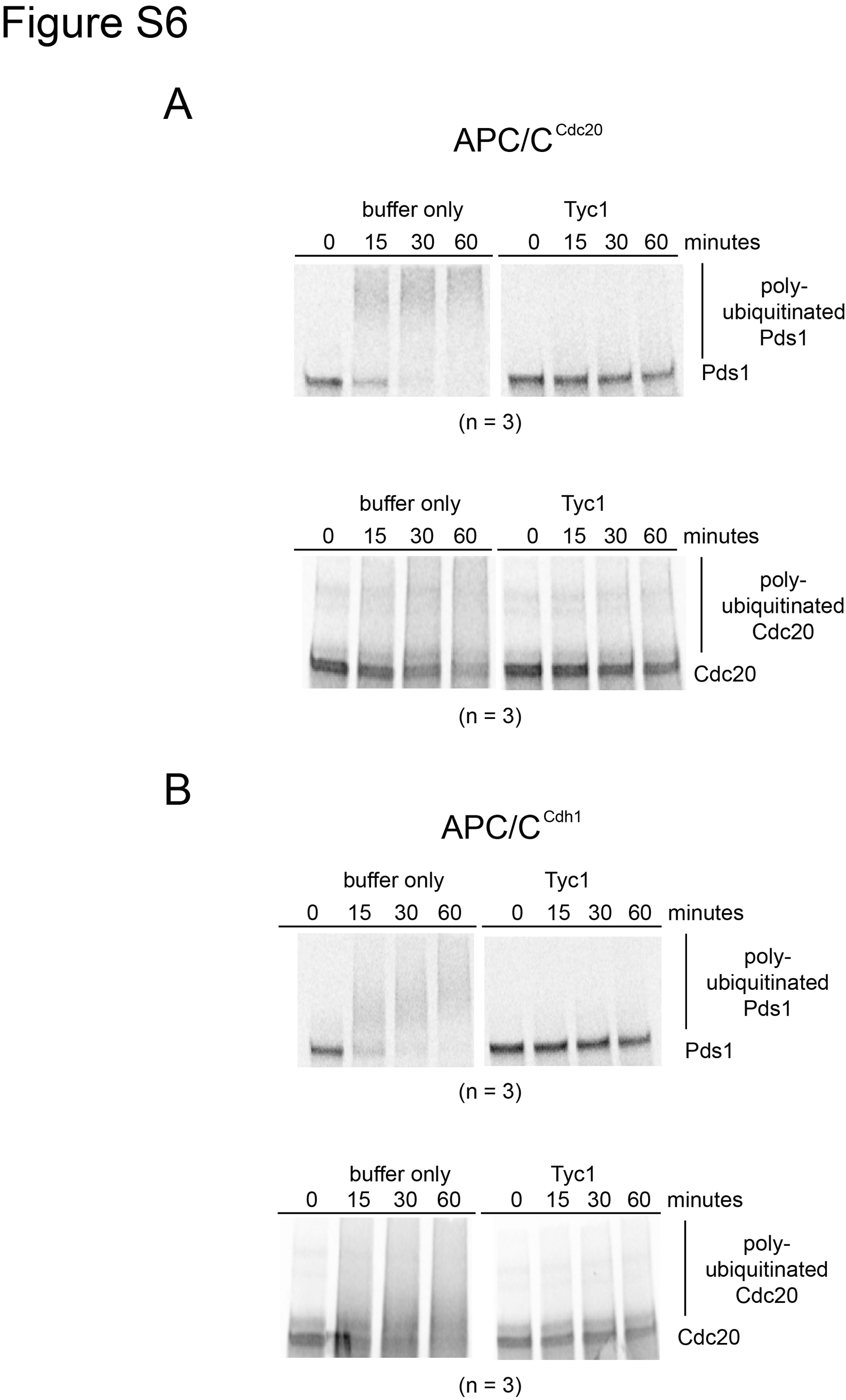

Supplement: S6 Fig — A) APC/CCdc20 reactions using Pds1 (top) and Cdc20 (bottom) as the target substrates. Tyc1 inhibits APC/CCdc20 activity in both reactions, but the quantification of the Cdc20 substrate results is confounded by the presence of lower molecular weight forms that also migrate up the gel over time. B) APC/CCdh1 reactions using Pds1 (top) and Cdc20 (bottom) as the target substrates. Tyc1 inhibits APC/CCdh1 activity in both reactions, but the quantification of the Cdc20 substrate results is confounded by the presence of lower molecular weight forms that also migrate up the gel over time. (TIF) [file pone.0198930.s006.tif]

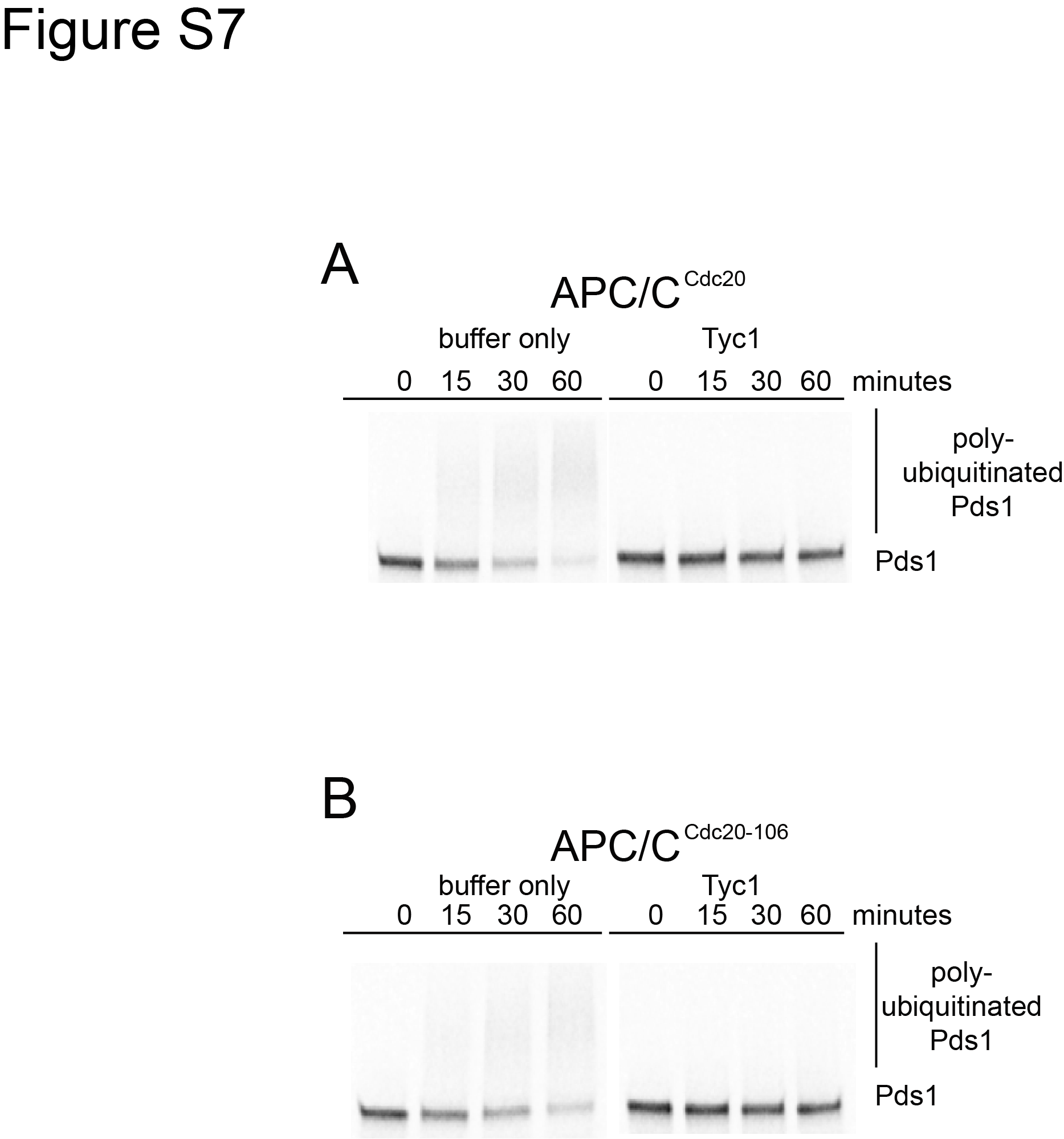

Supplement: S7 Fig — A) APC/CCdc20 reactions using the wild type Cdc20 are shown as a positive control. B) APC/CCdc20-106 reactions are also inhibited by Tyc1. The Cdc20-106 (P209Q) allele was chosen because this allele can promote a normal level of APC/C activity but was not inhibited by Mad2 (see S5 and S6 Tables). (TIF) [file pone.0198930.s007.tif]

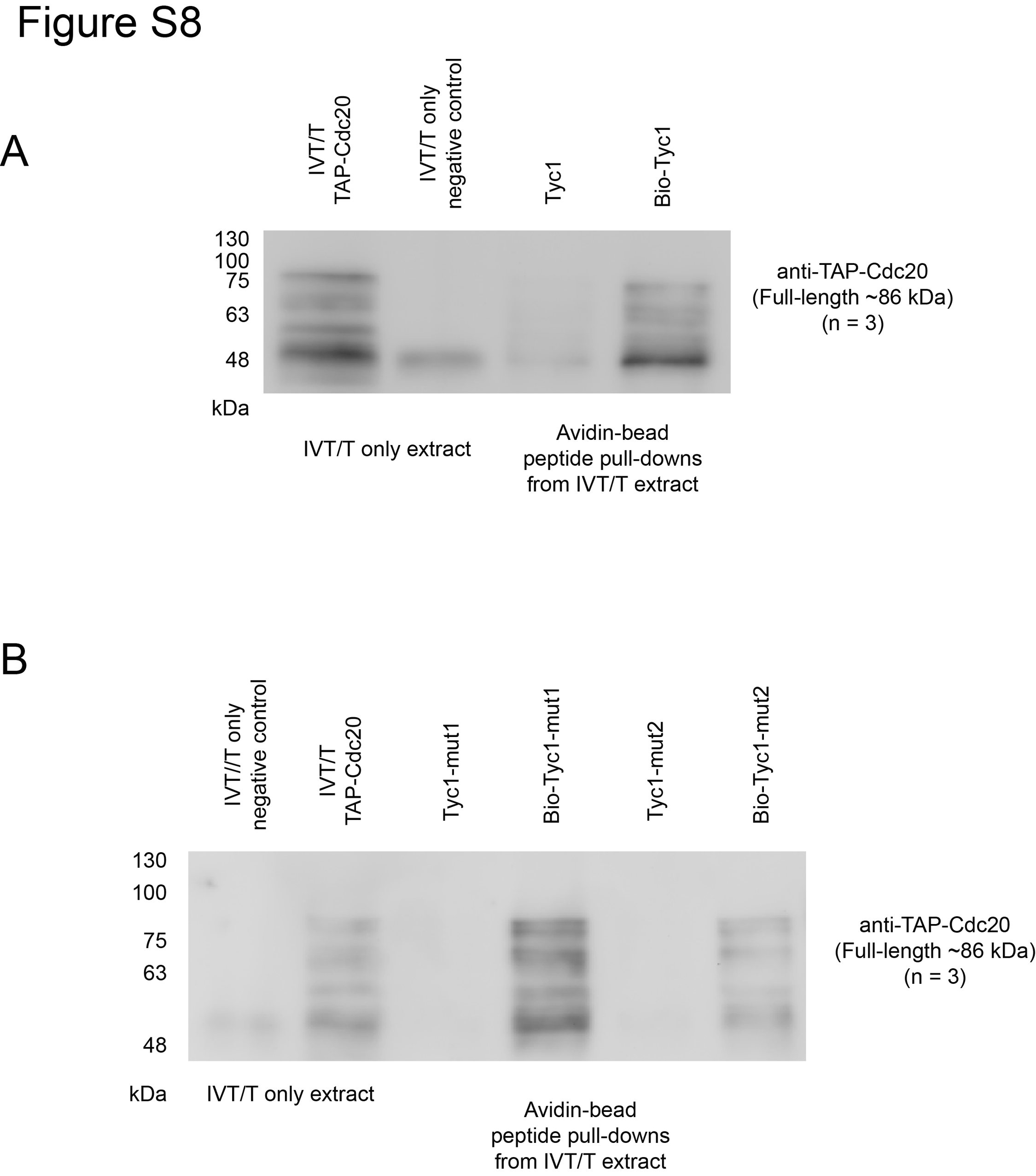

Supplement: S8 Fig — A) A Western blot displaying the detection of Cdc20 protein made by IVT/T (left), where the epitope tag is at the N-terminus of the ORF. The IVT/T reaction yields a series of protein products that display full-length and lower molecular weights, potentially the result of pre-mature translation termination. An Avidin-bead pull-down using a biotinylated-Tyc1 (Bio-Tyc1) is shown (right). Both full-length and lower molecular weight forms of Cdc20 were observed that all contain the N-terminal region of Cdc20 where the TAP-epitope tag is located. B) An Avidin-bead pull-down using a biotinylated-Tyc1-mut1 (Bio-Tyc1-mut1) (middle) and biotinylated-Tyc1-mut2 (Bio-Tyc1-mut2) are shown (right). We consistently observed (n = 3) that the amount of Cdc20 protein isolated on the Avidin-beads in the presence of Bio-Tyc1-mut2 was lower. (TIF) [file pone.0198930.s008.tif]
