# Supplementary material for: Peptide inhibitors of the anaphase promoting-complex that cause sensitivity to microtubule poison
Source: PLoS One. 2018 Jun 8;13(6):e0198930. doi: 10.1371/journal.pone.0198930 (PMC5993284; doi:10.1371/journal.pone.0198930)

Fig 1B

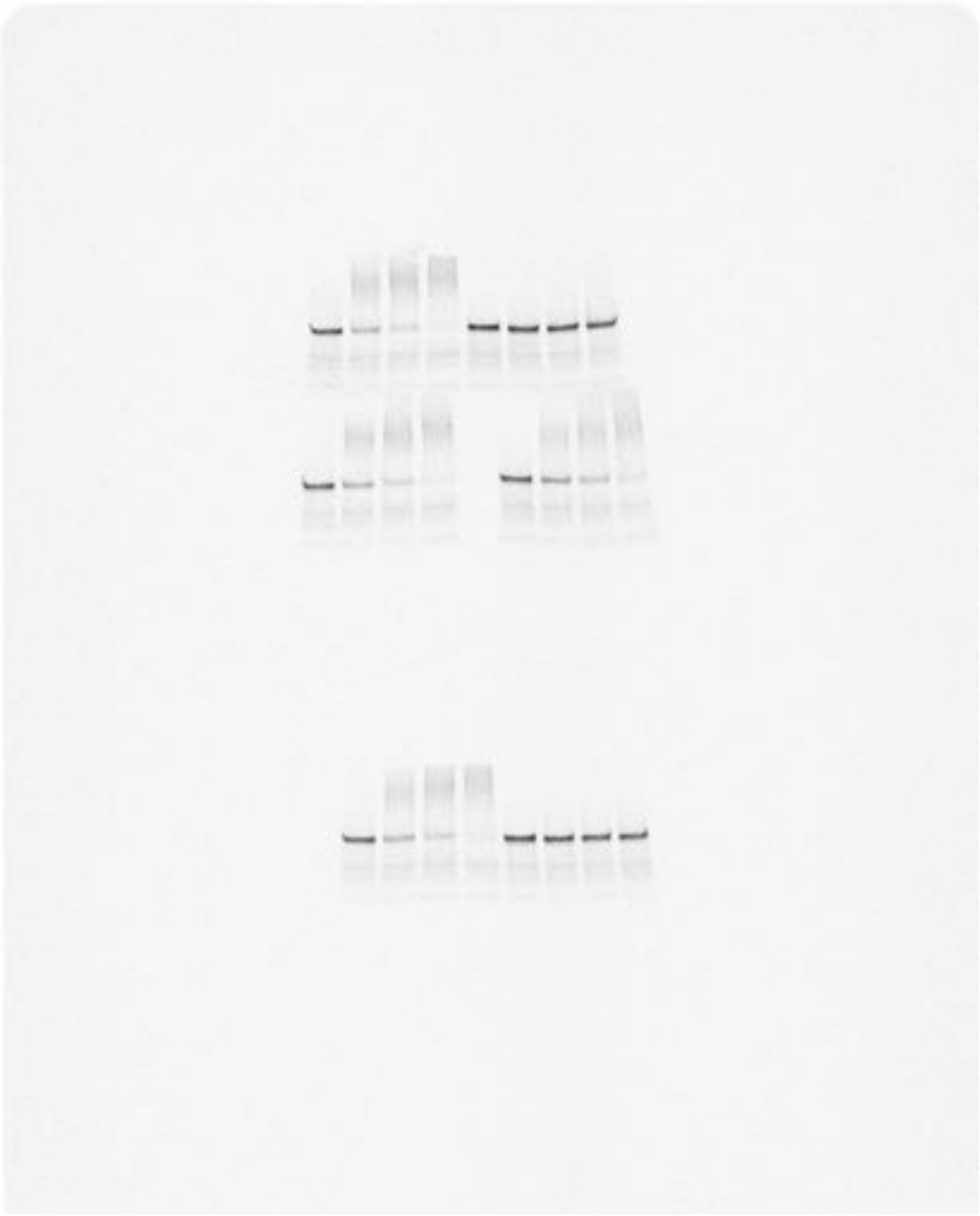

Fig 1C

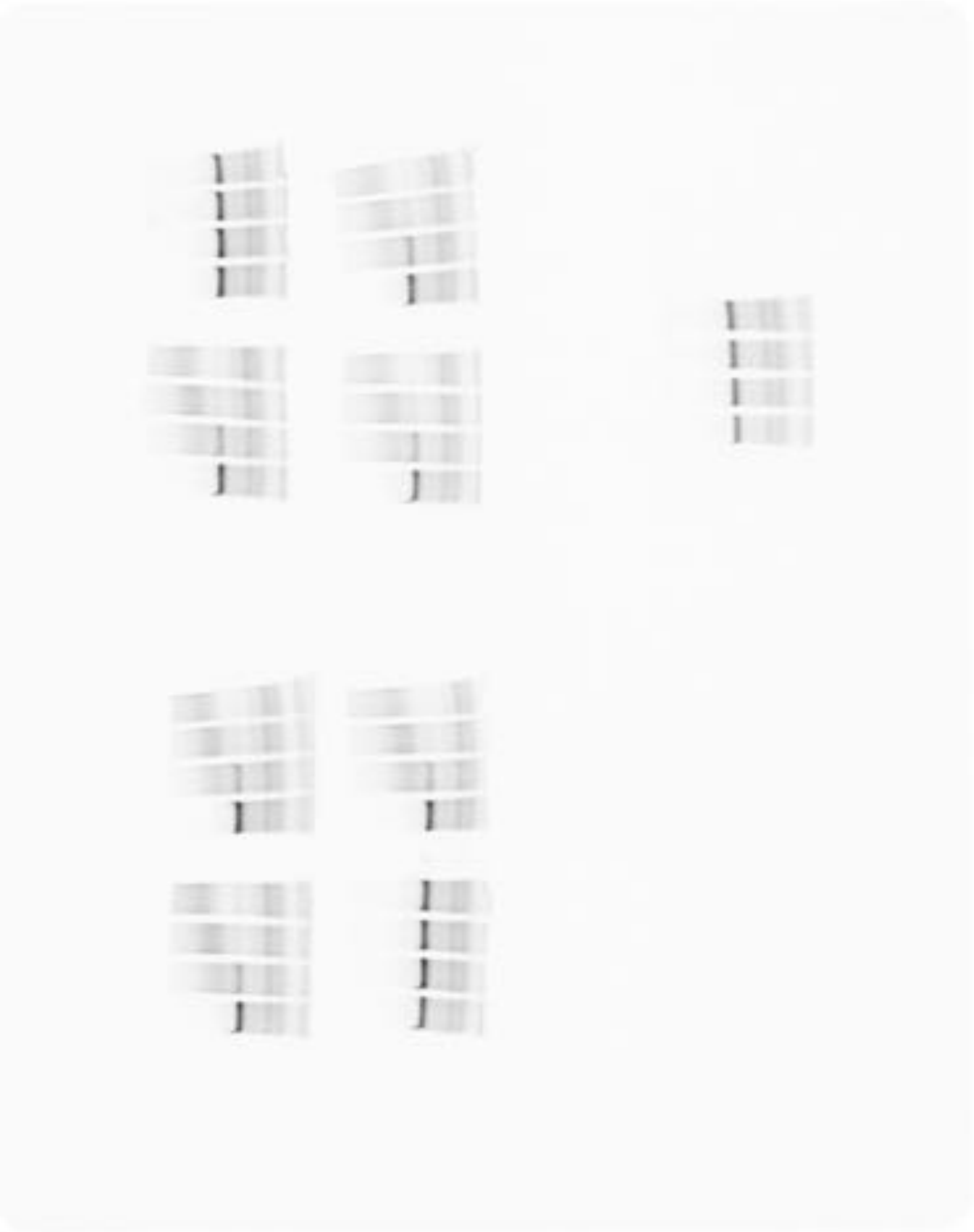

Fig 2B

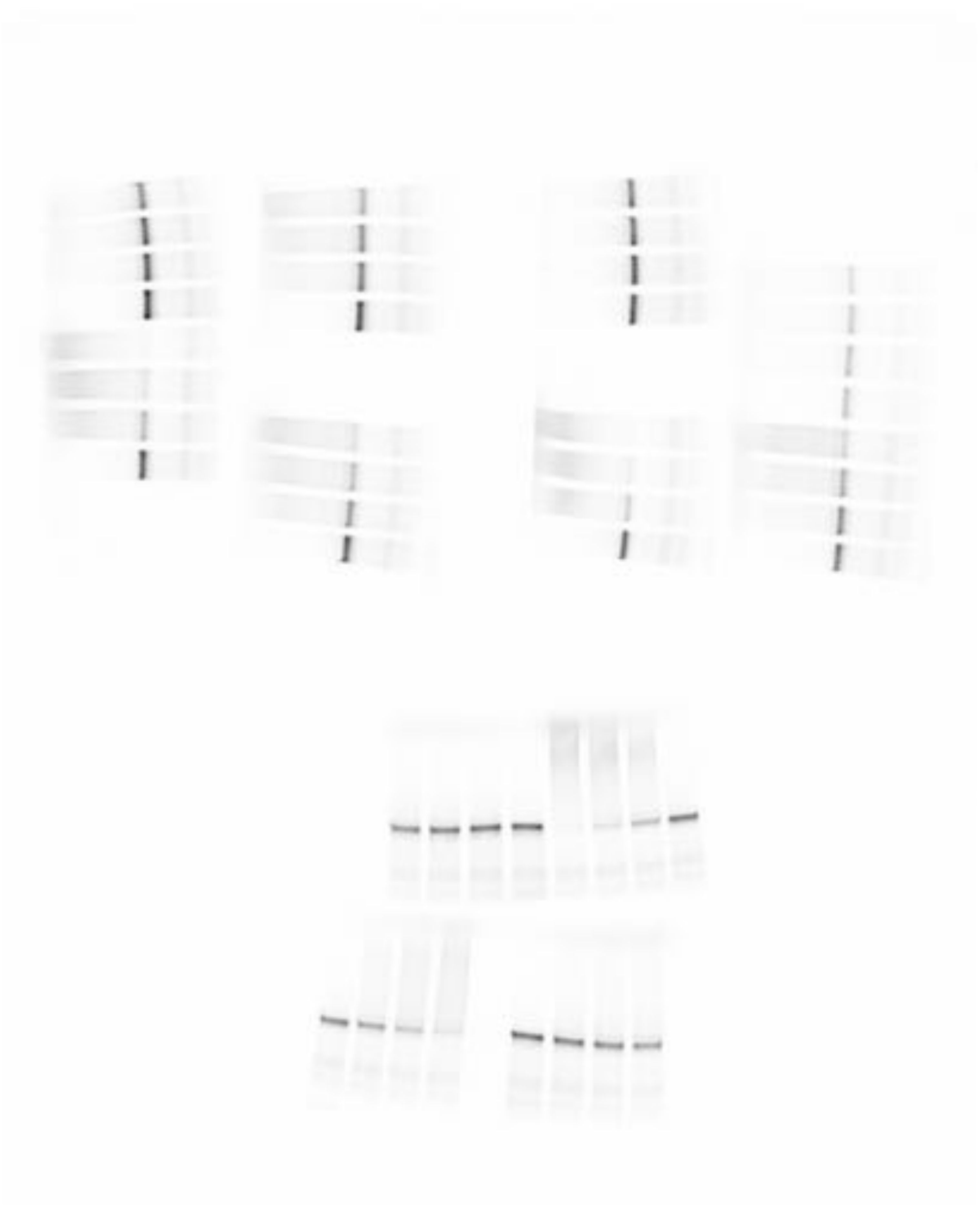

Fig 2C

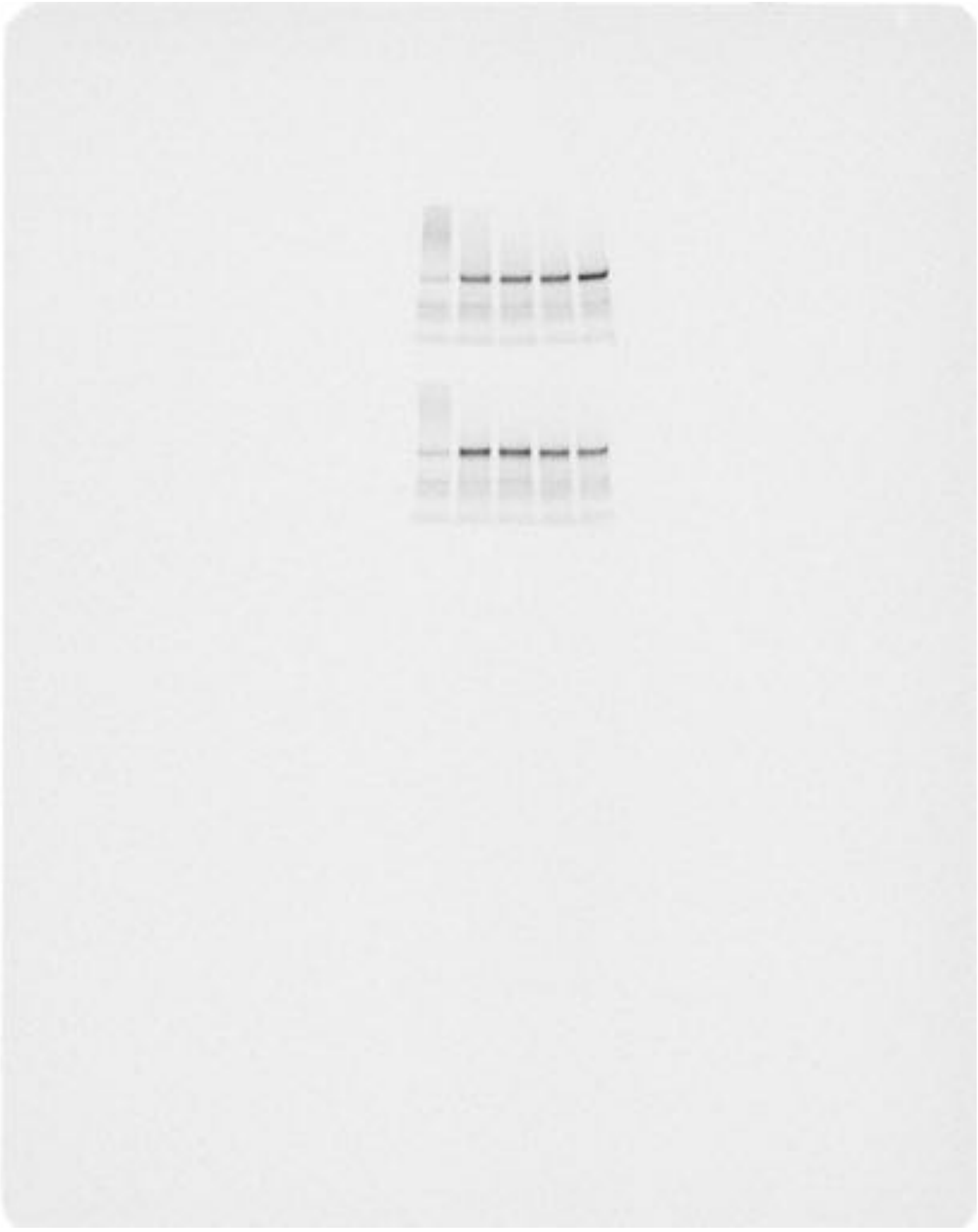

Fig 2D

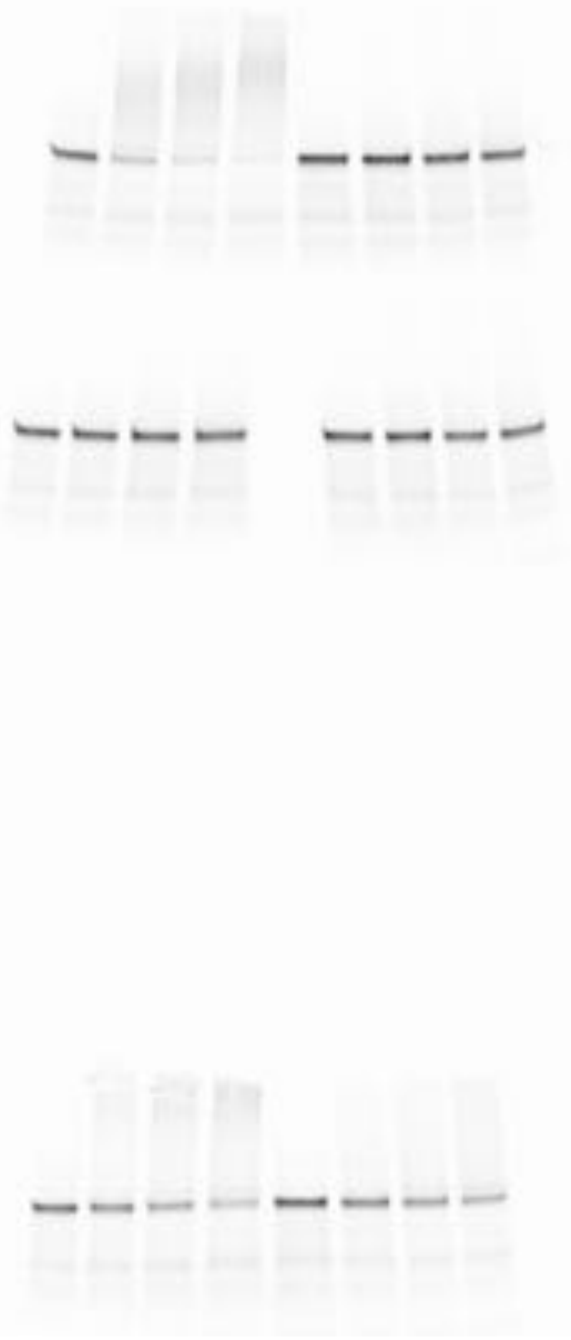

Fig 3B

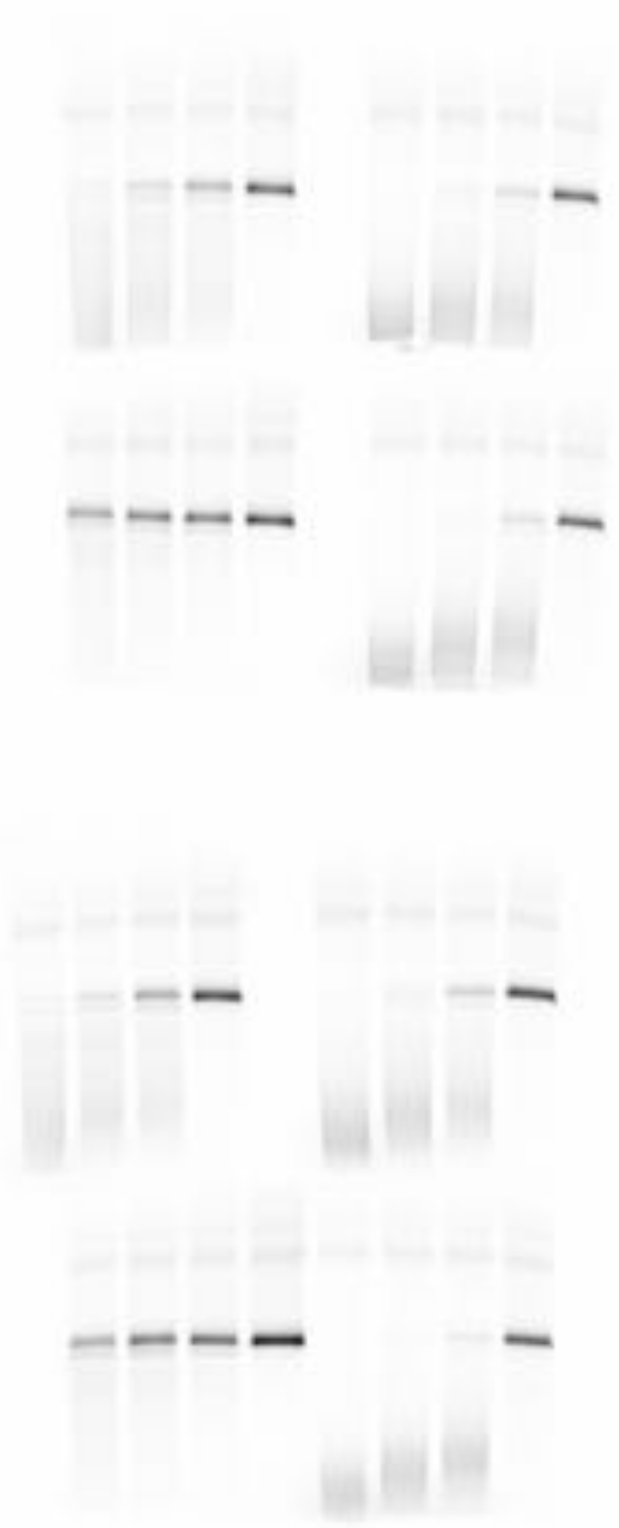

Fig 3C

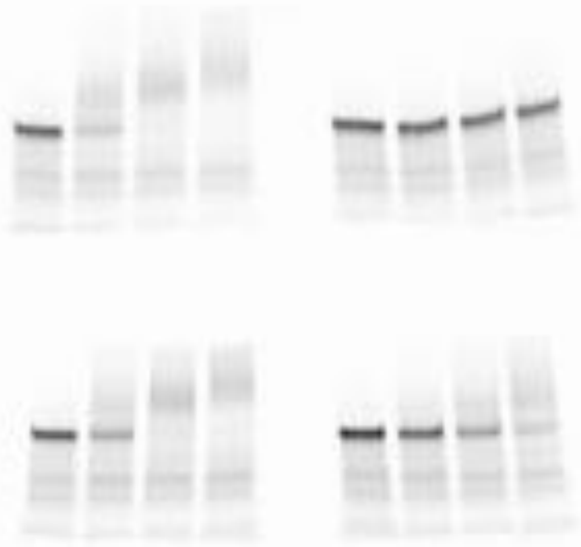

Fig 3C

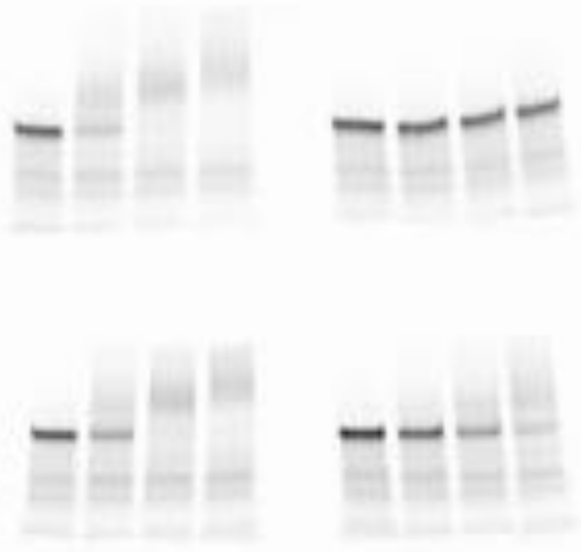

Fig 3D

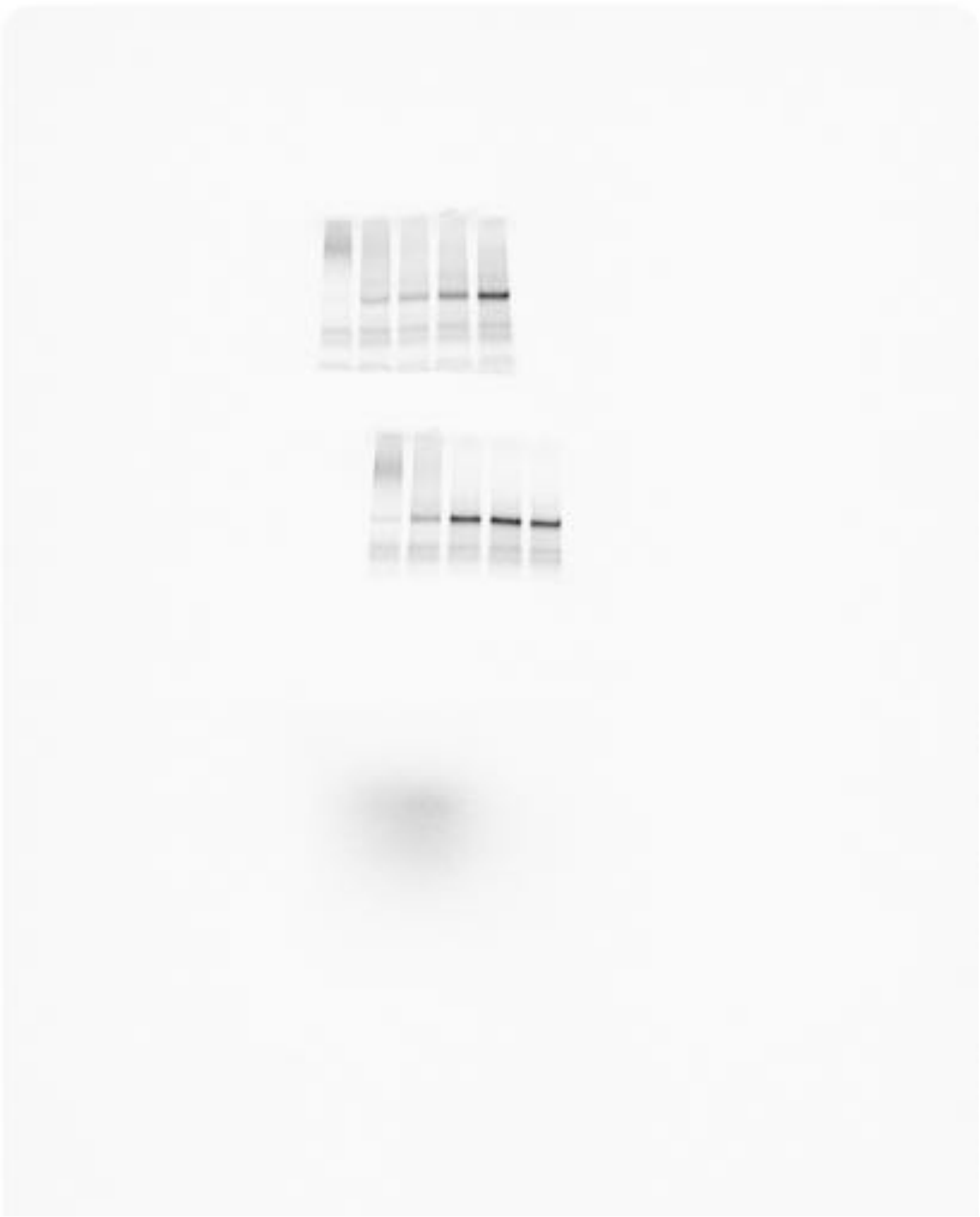

Fig 4A

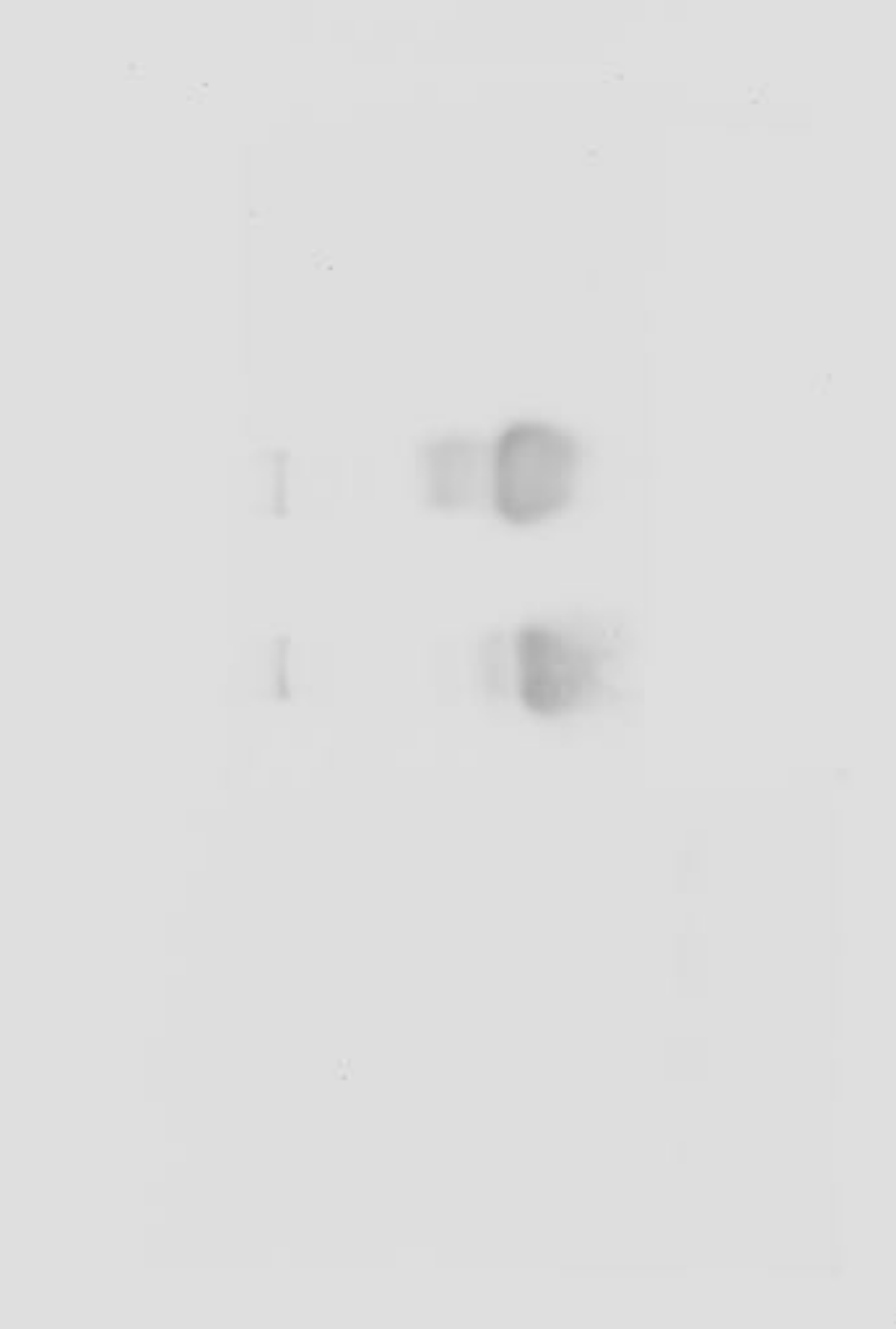

Fig 4A-1

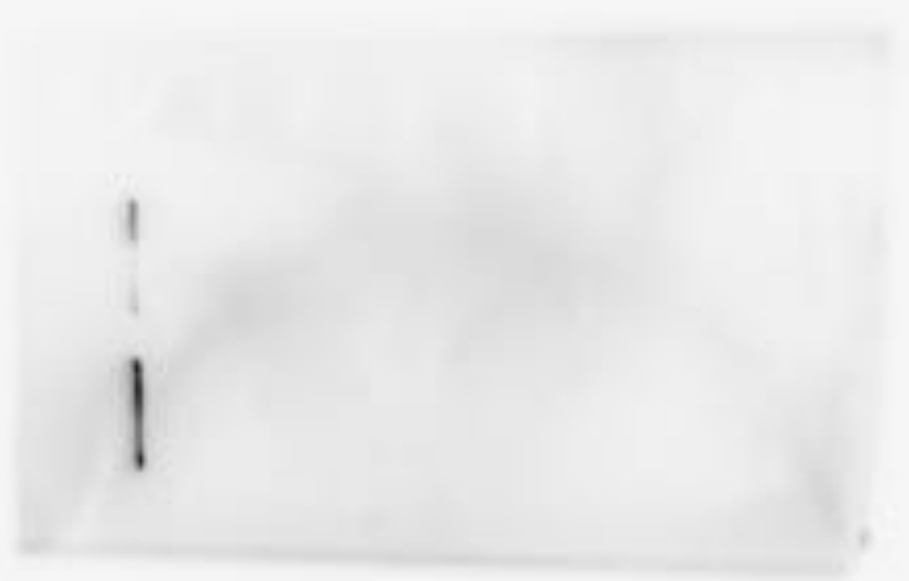

Fig 4A-2

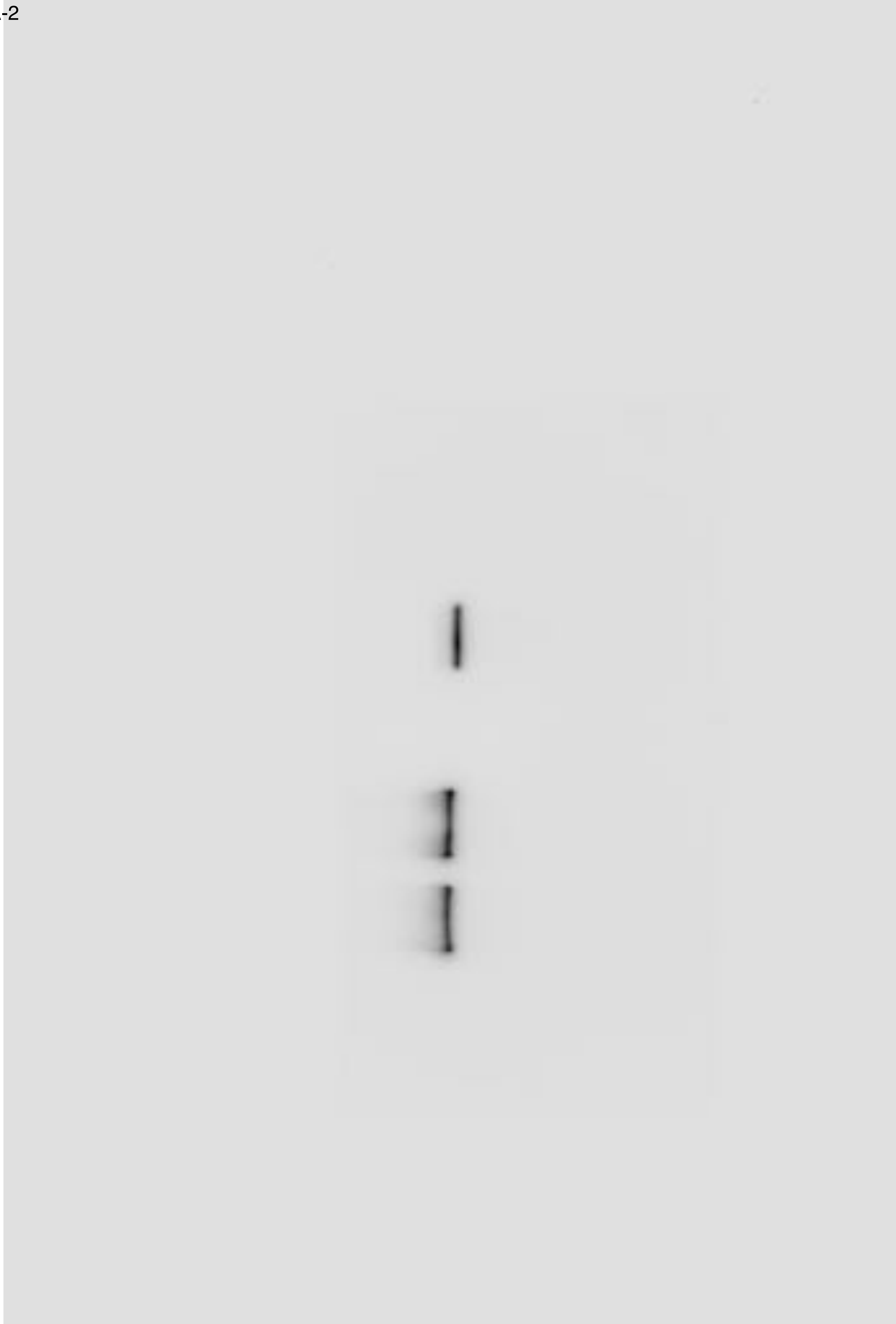

Fig 4B

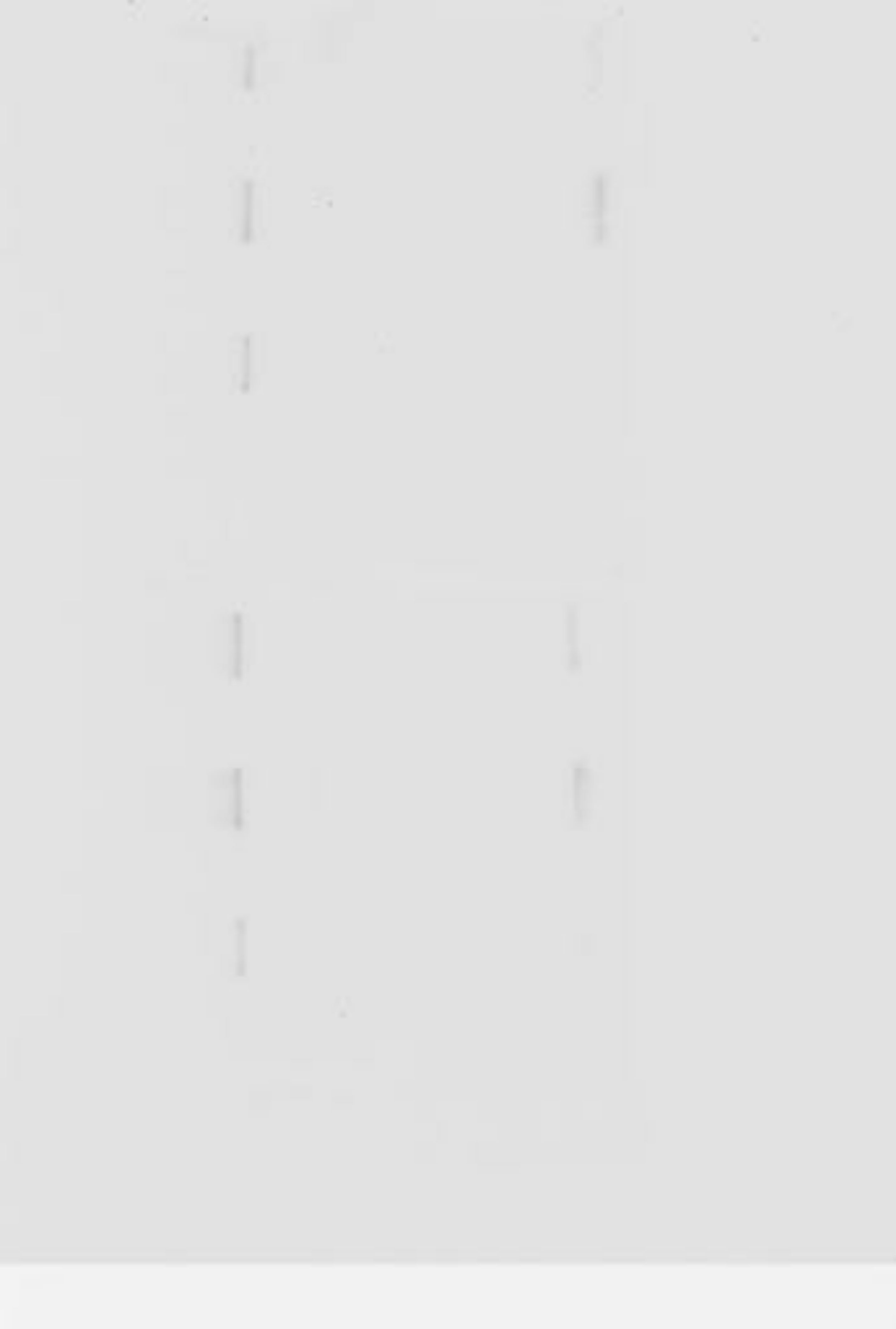

Fig 4B-1

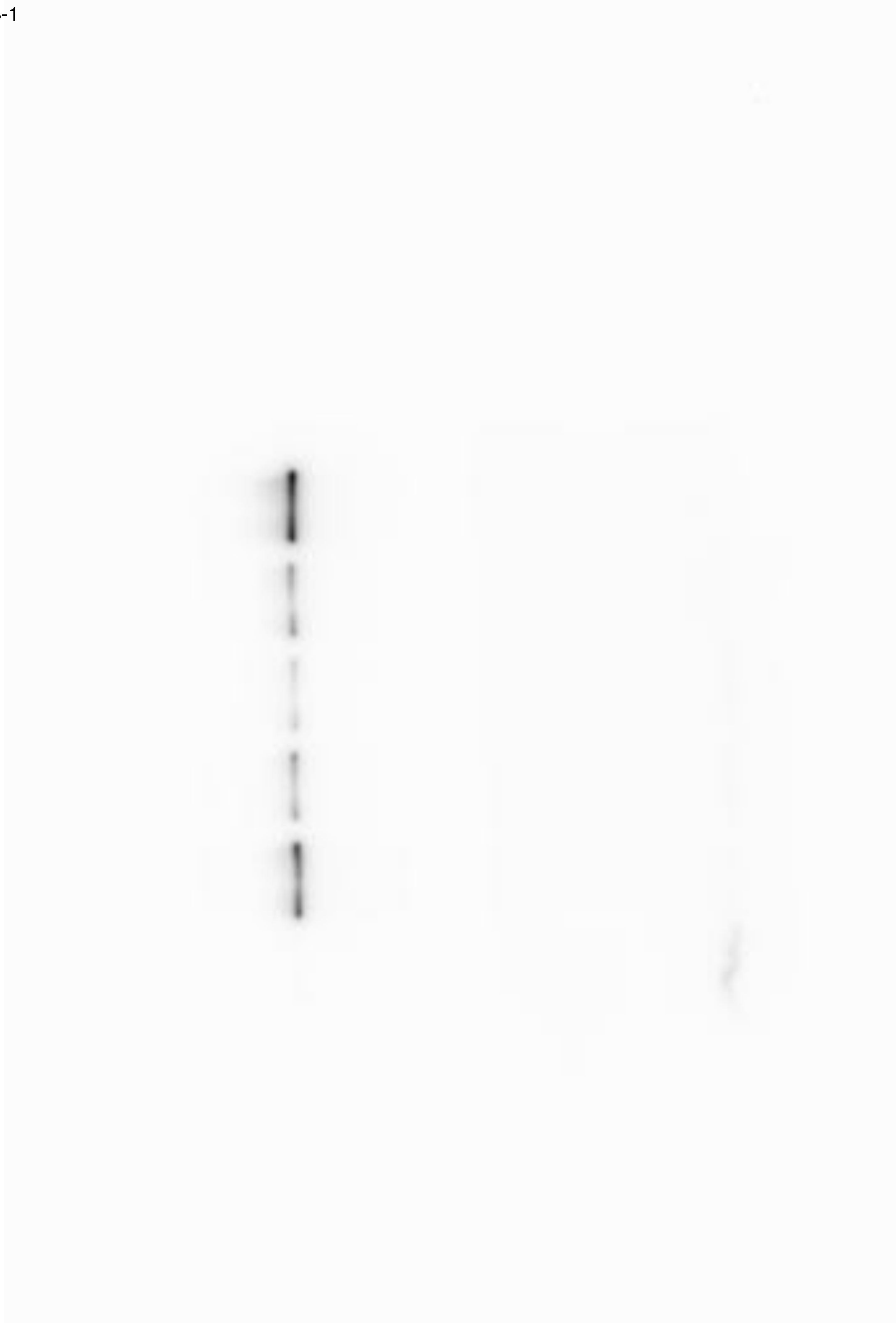

Fig 4B-2

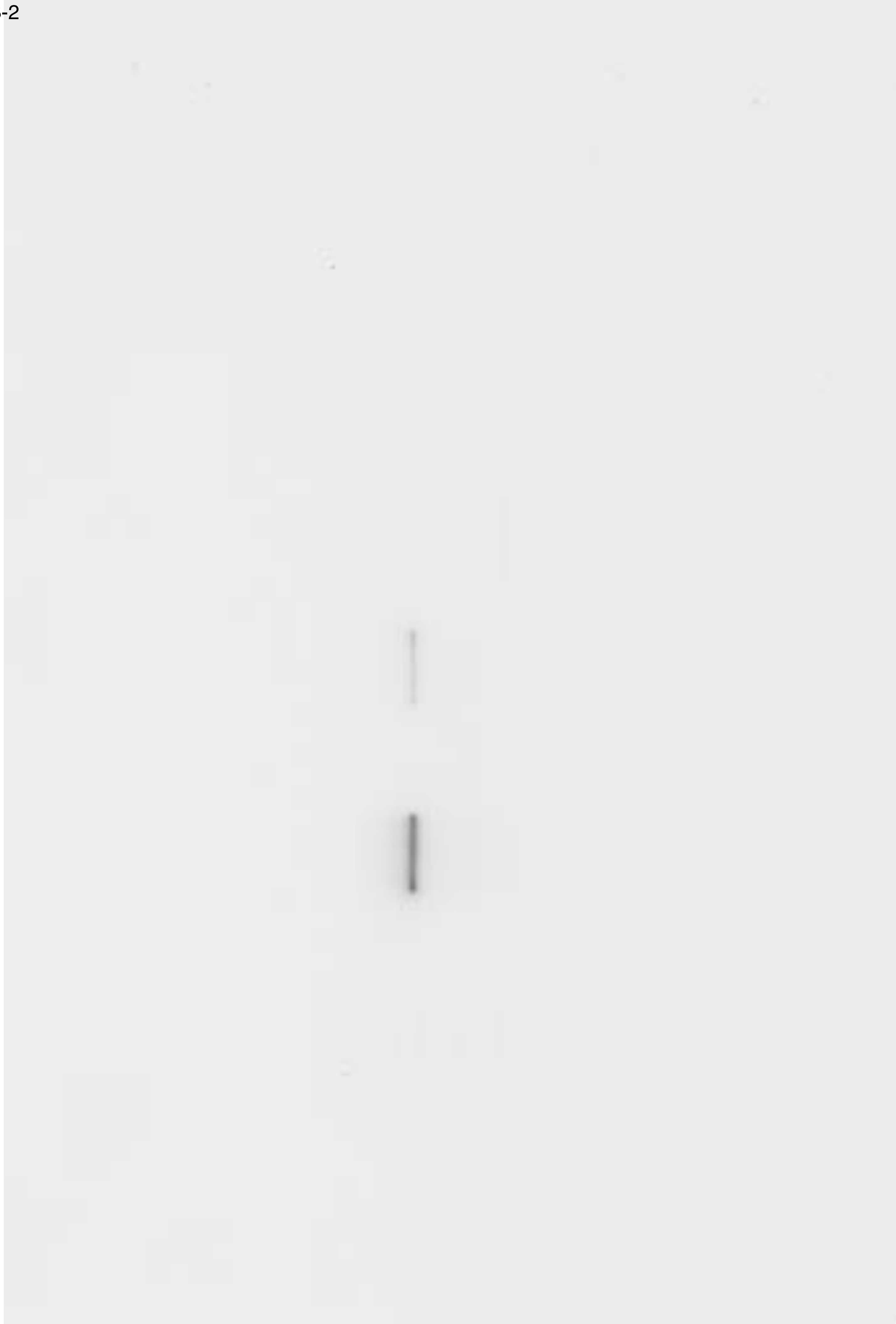

Fig 5B

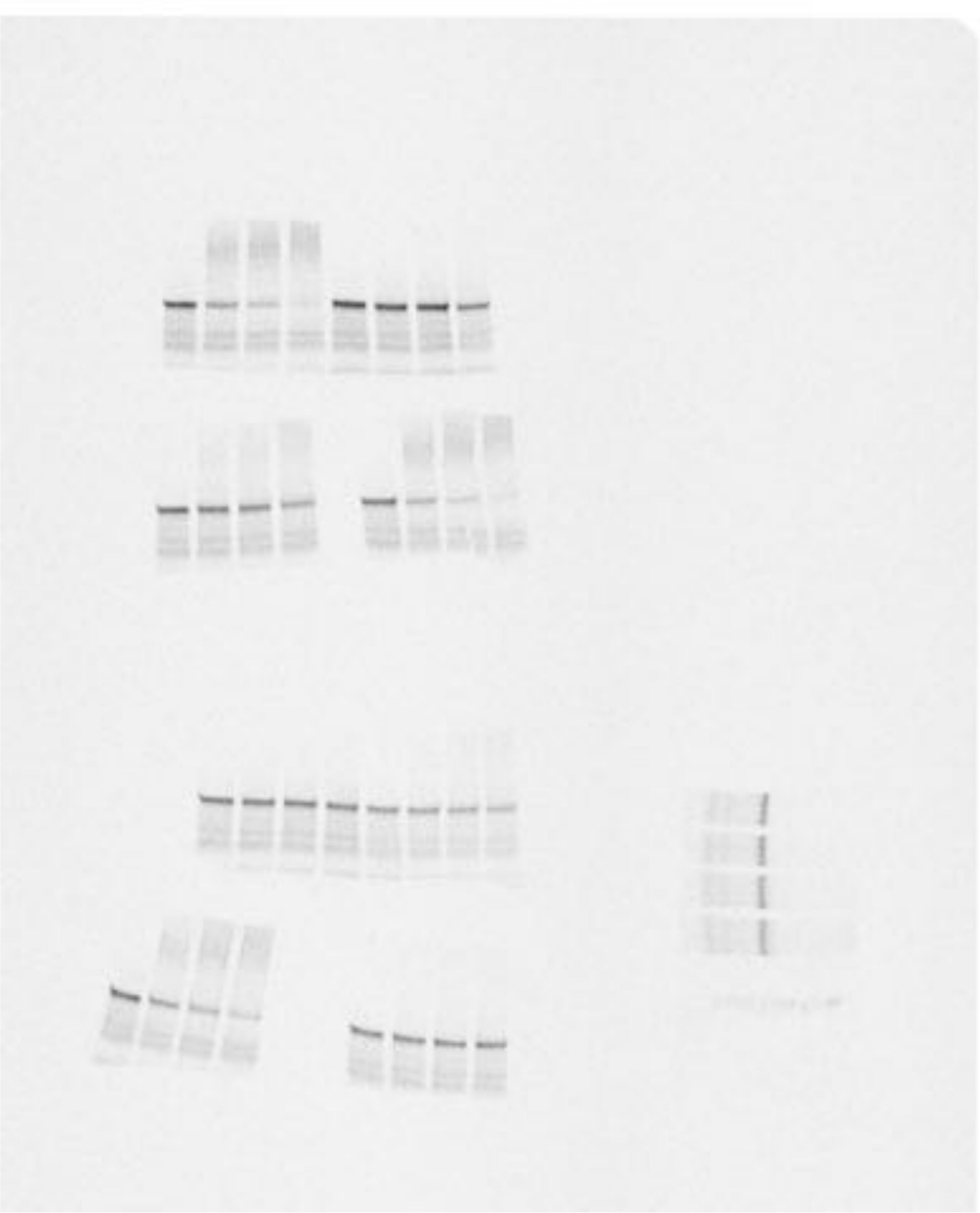

Fig 5C

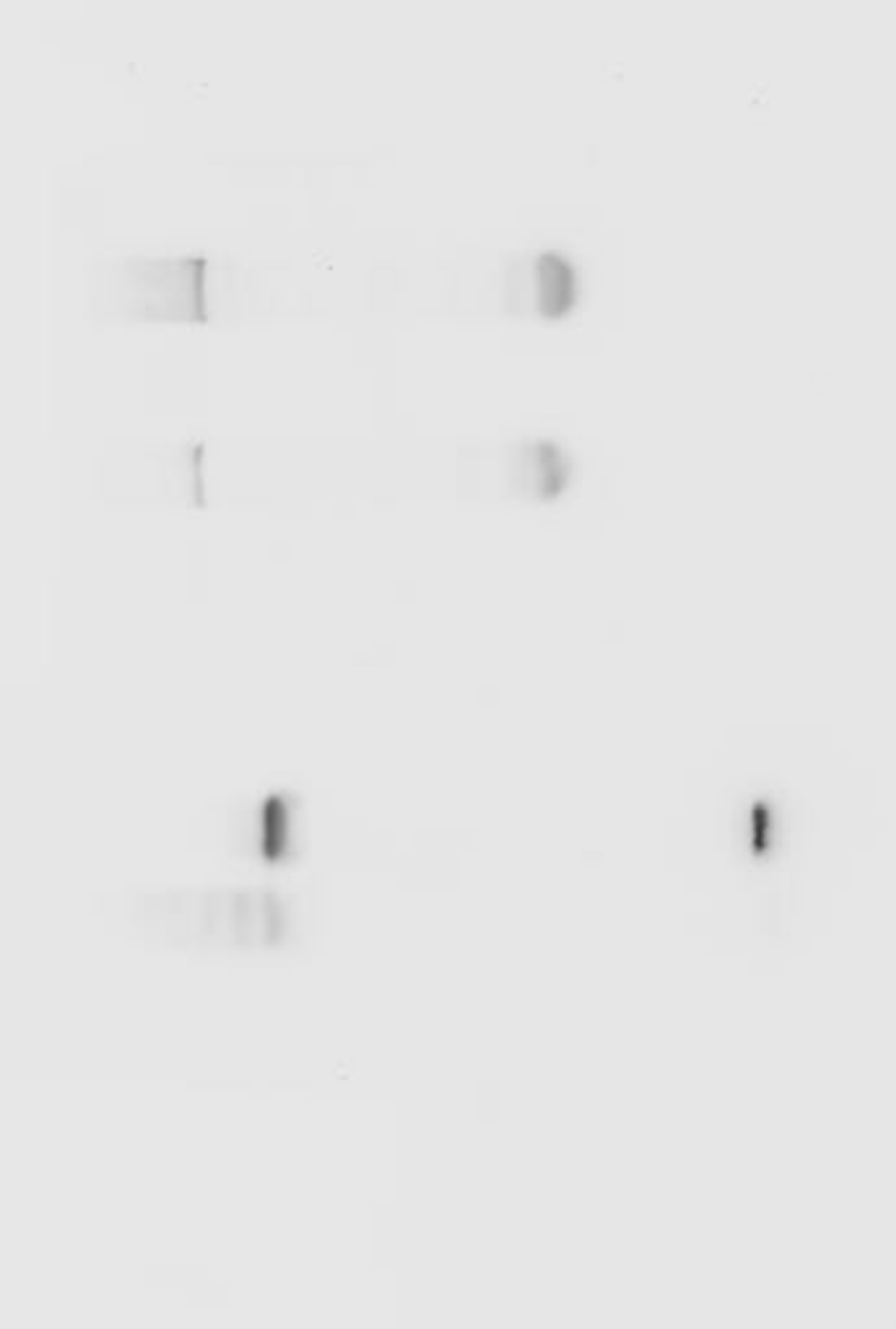

Fig 5D

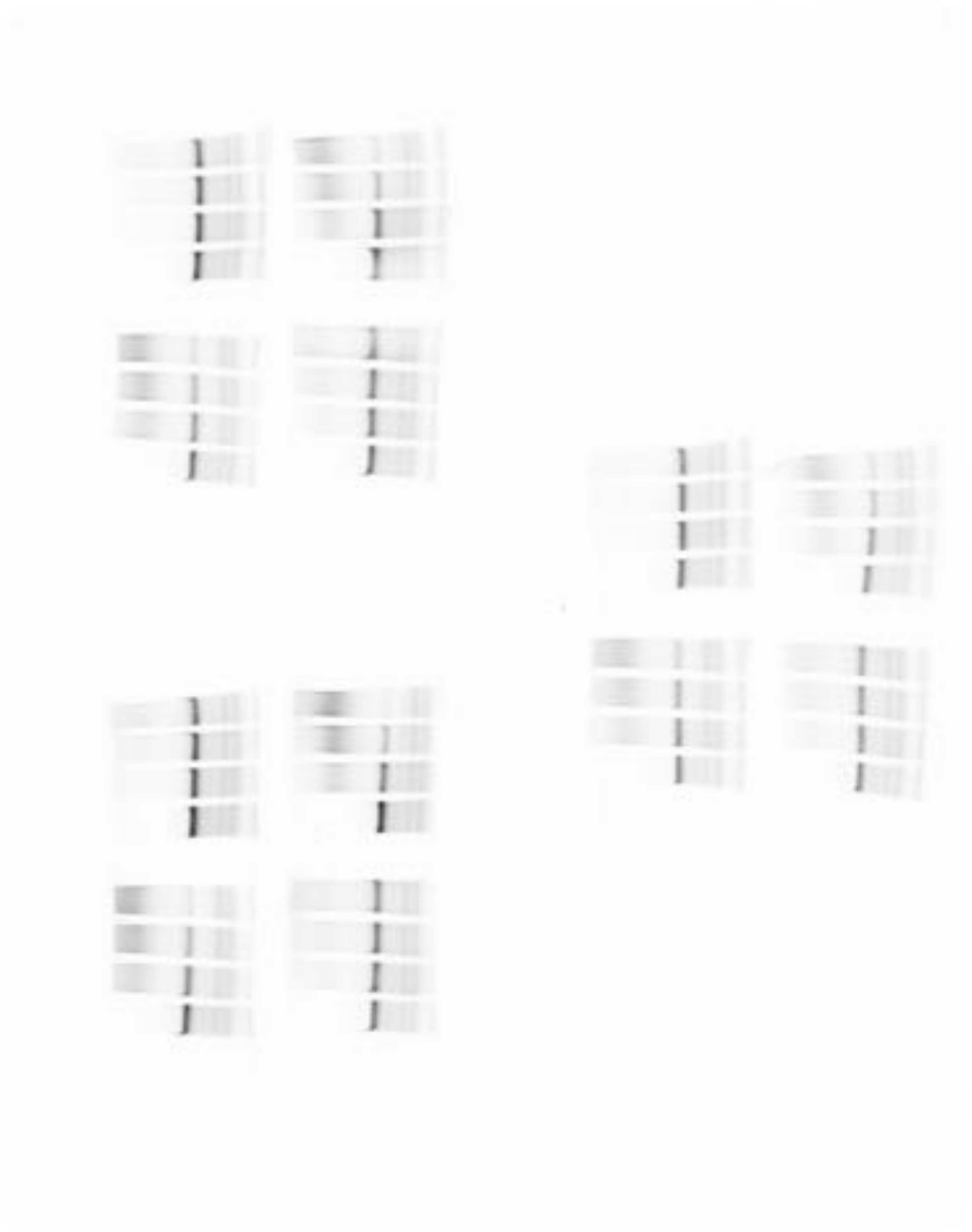

Fig 6B

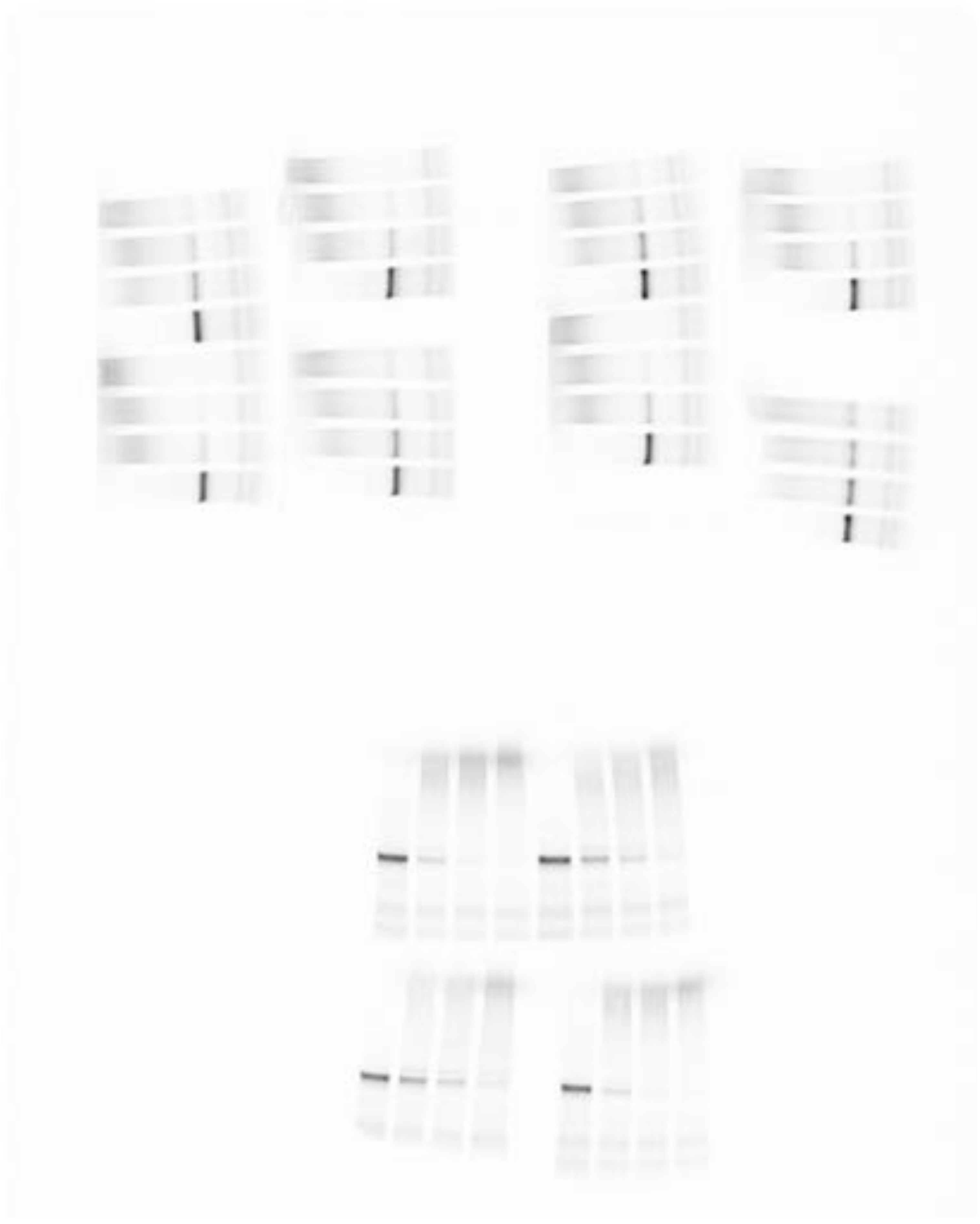

Fig S6C

Fig S6C-1

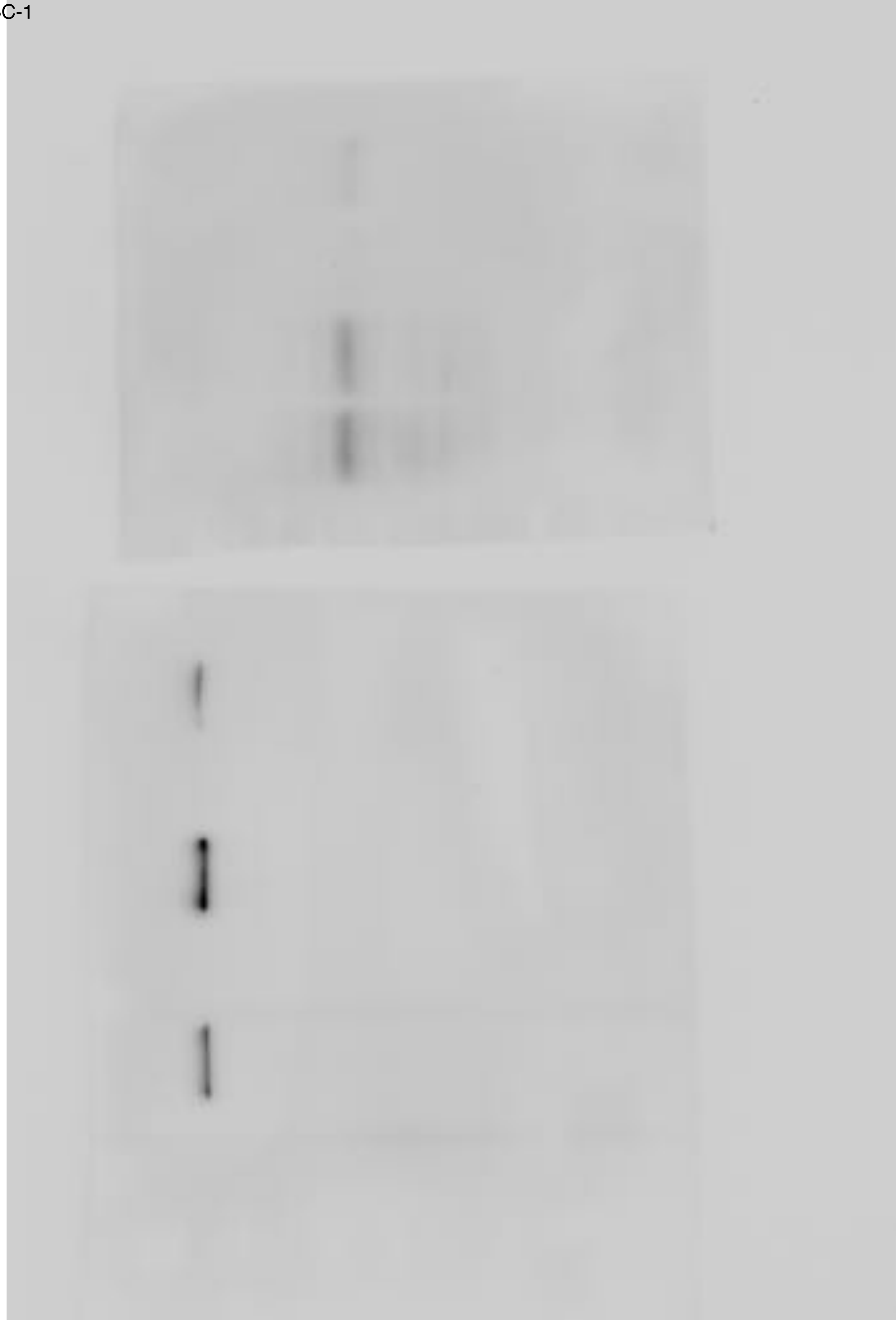

Fig 6D

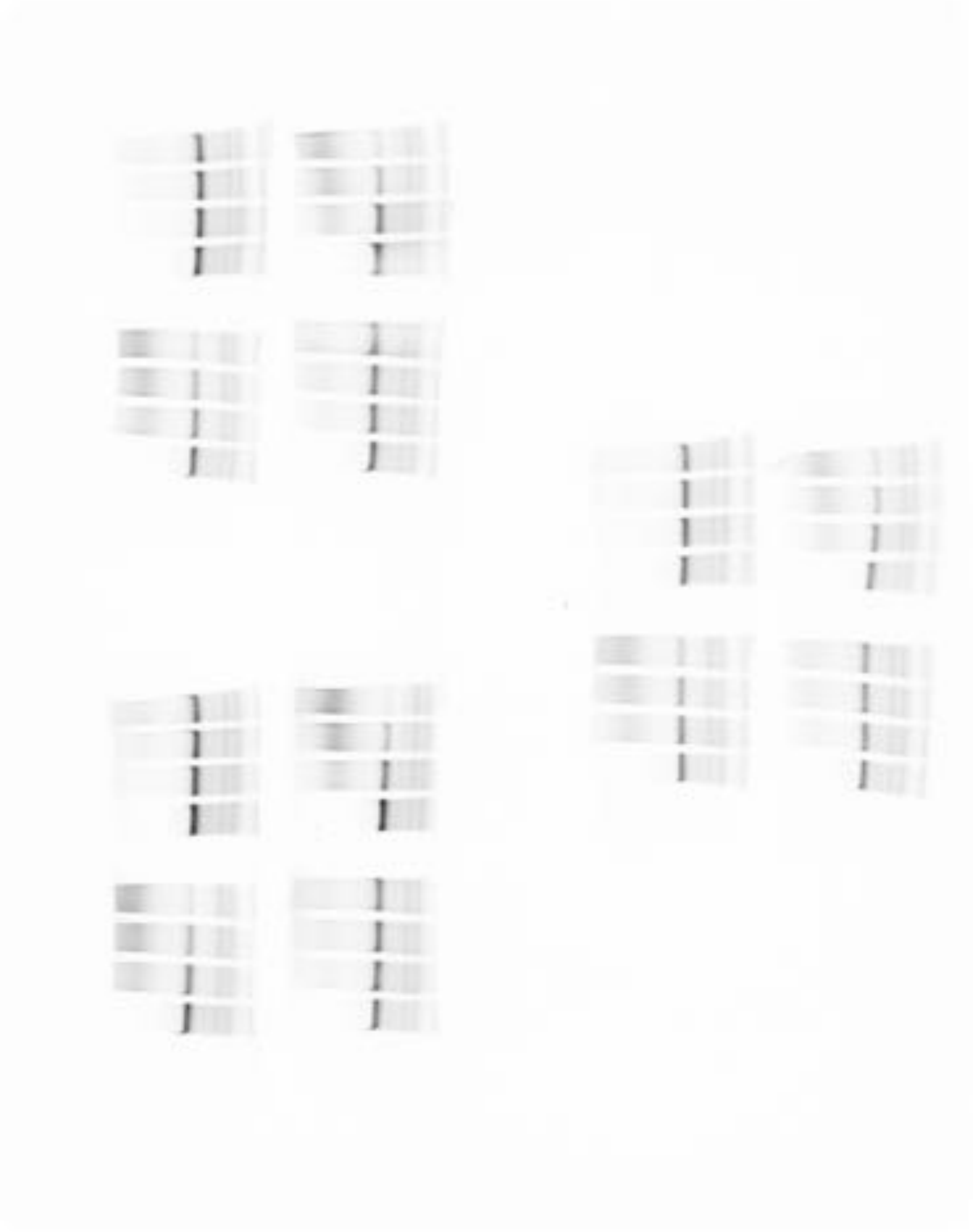

Fig 7C

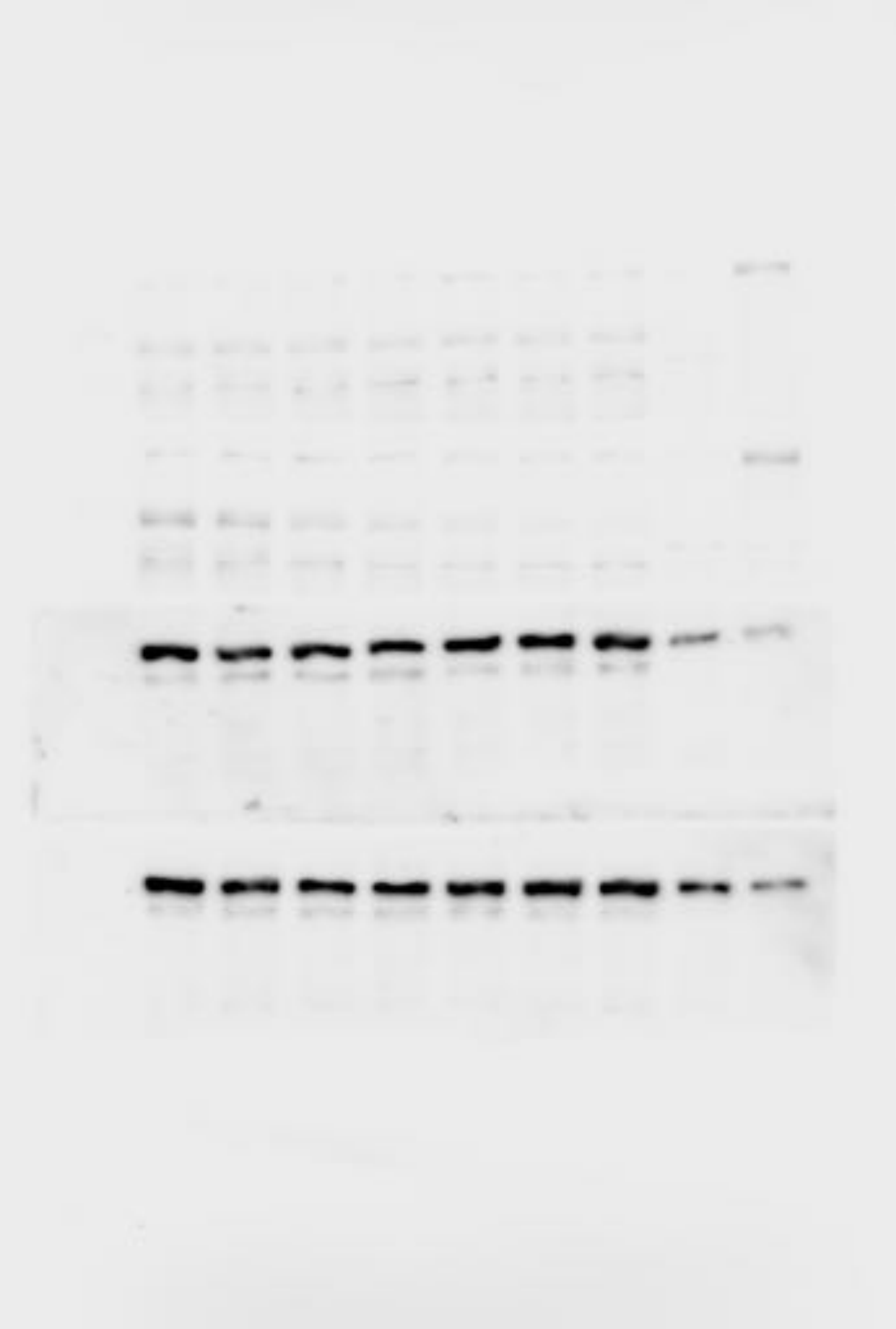

Fig 9B

2012/06/14

V: VILO (5/3)
P: gDNA
N: NTC

[illegible]

DNA	1.5
10×P1	1
20×P2	1
dNTP	2
10×Buf	2.5
enzyme	0.5
H_2O	16.5
	25

Tm = 50°C
40X

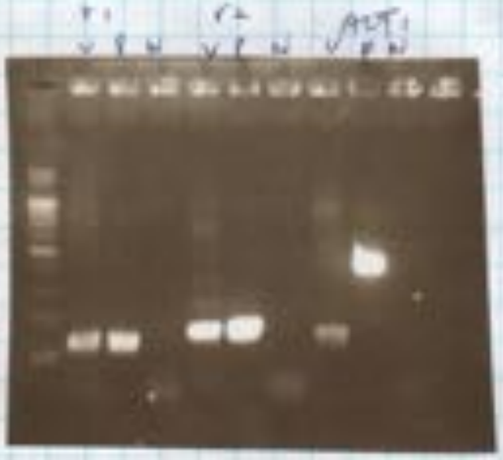

2012/06/15

∵ template 用完了, ∴ 試用 5/10 的 cDNA [illegible]

DNA 5/10 VILO H

Tm = ~~45~~ 50°C 40X

condition 与 6/14 相同

DNA	1
20×P1	1
20×P2	1
dNTP	2
10×Buf	2.5
enzyme	0.5
H_2O	17
	25

Tm = 52°C
40X

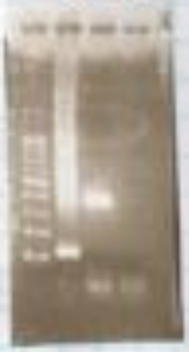

P1 P2 ACT1 ← [illegible]

V P N V P N V P N

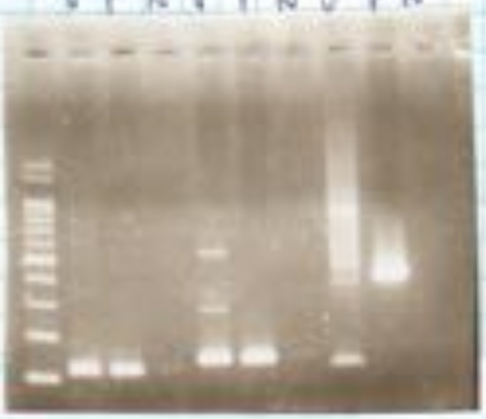

Fig 9C

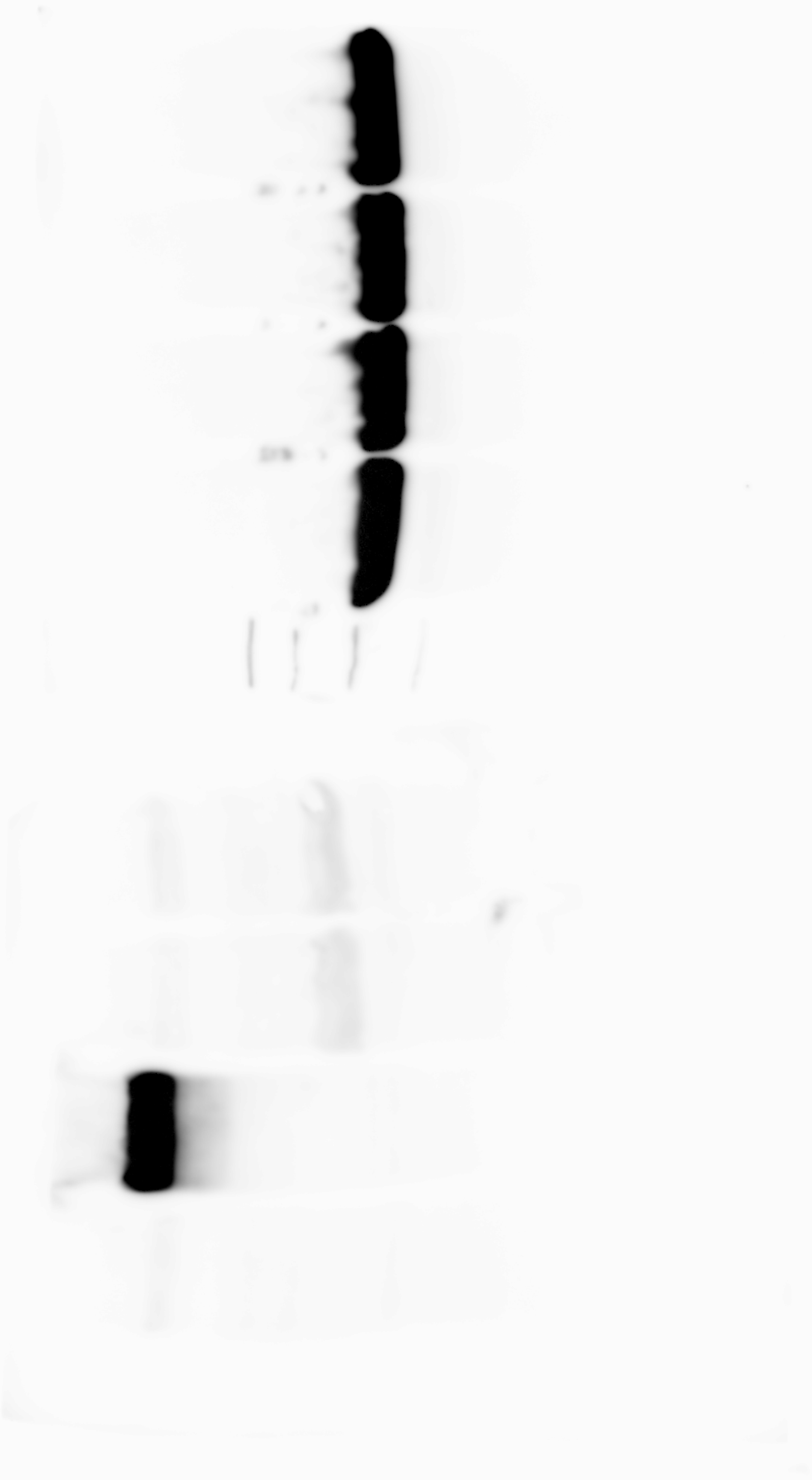

Fig 9D

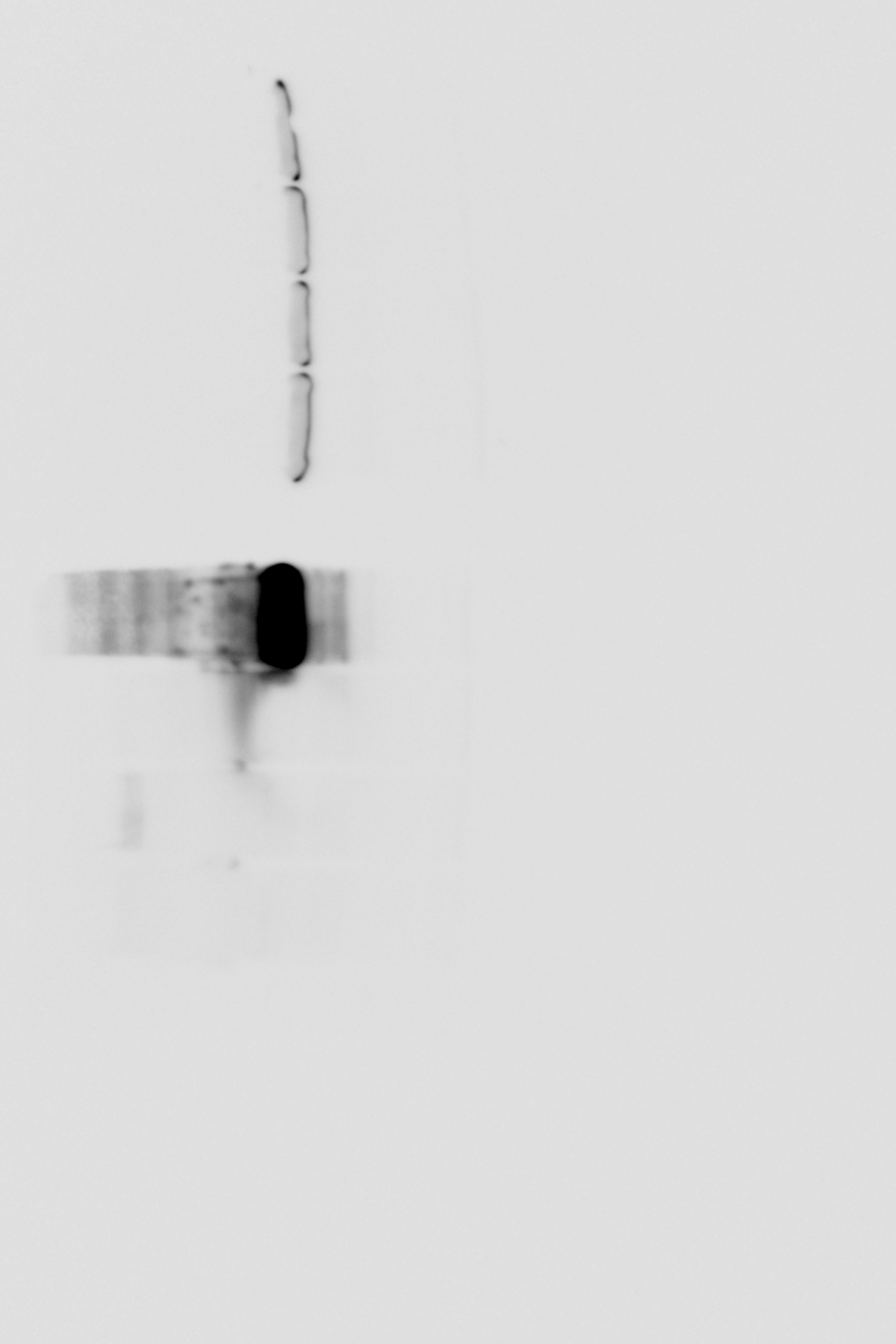

Fig 11B

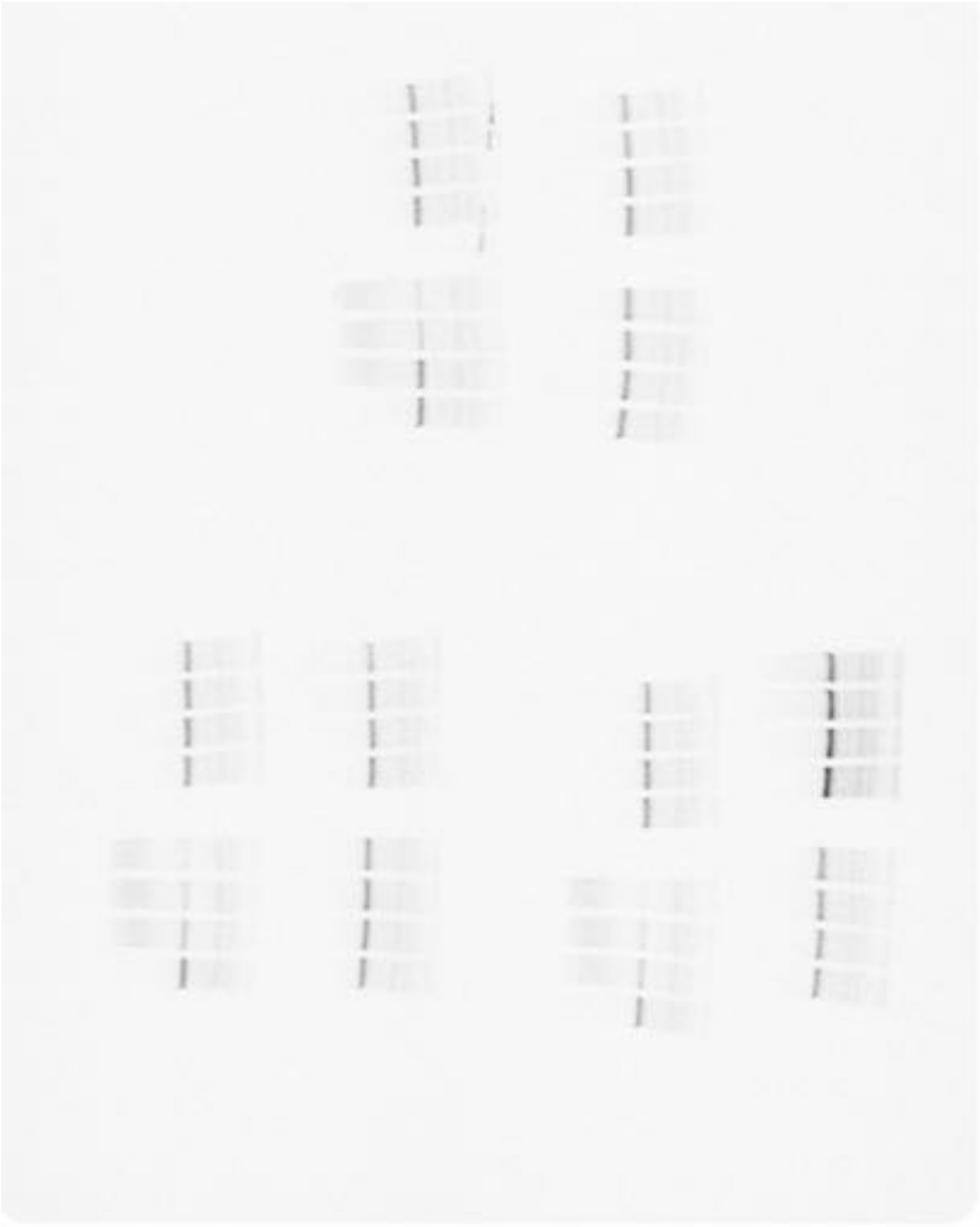

Fig 11C

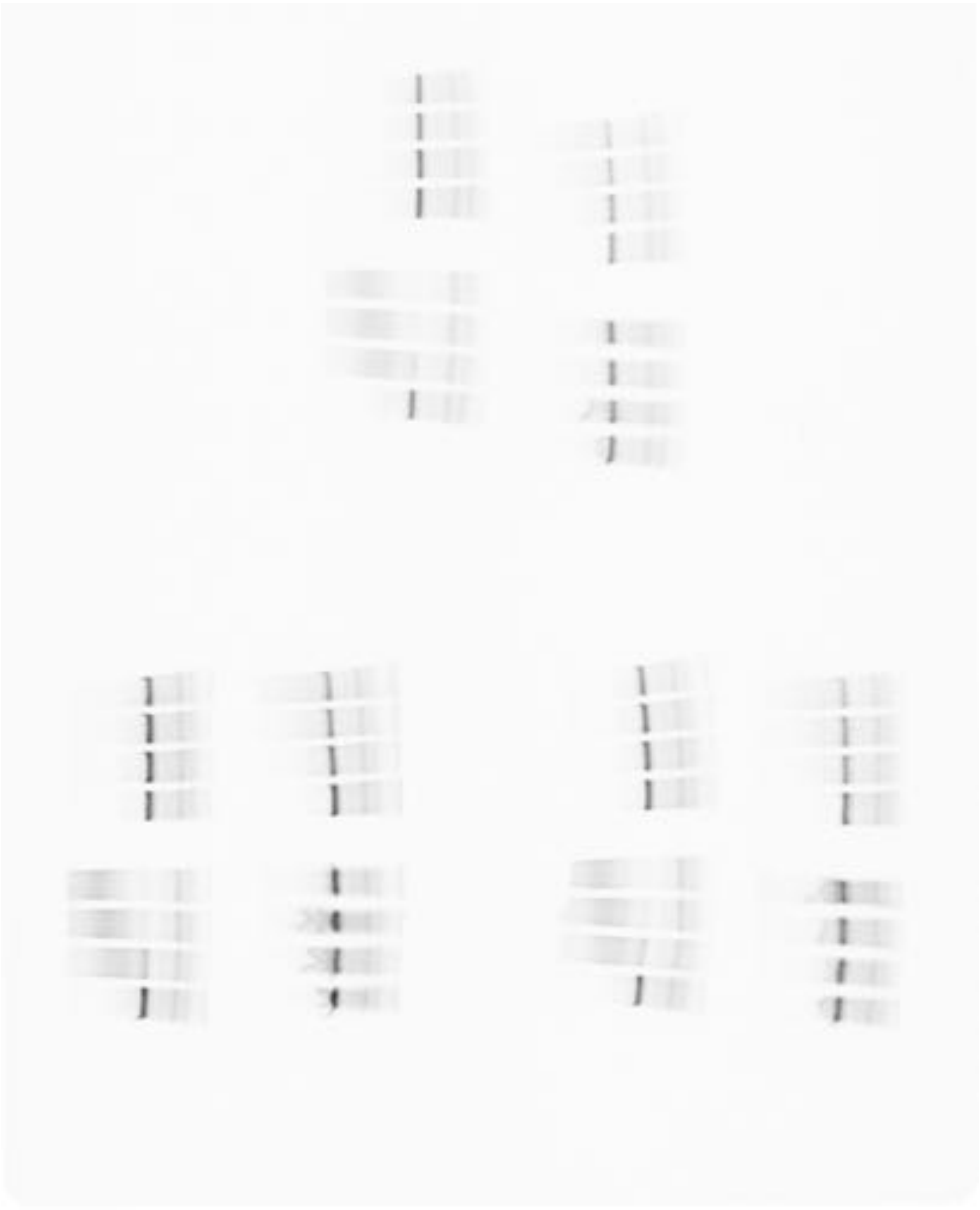

Fig 11C-1

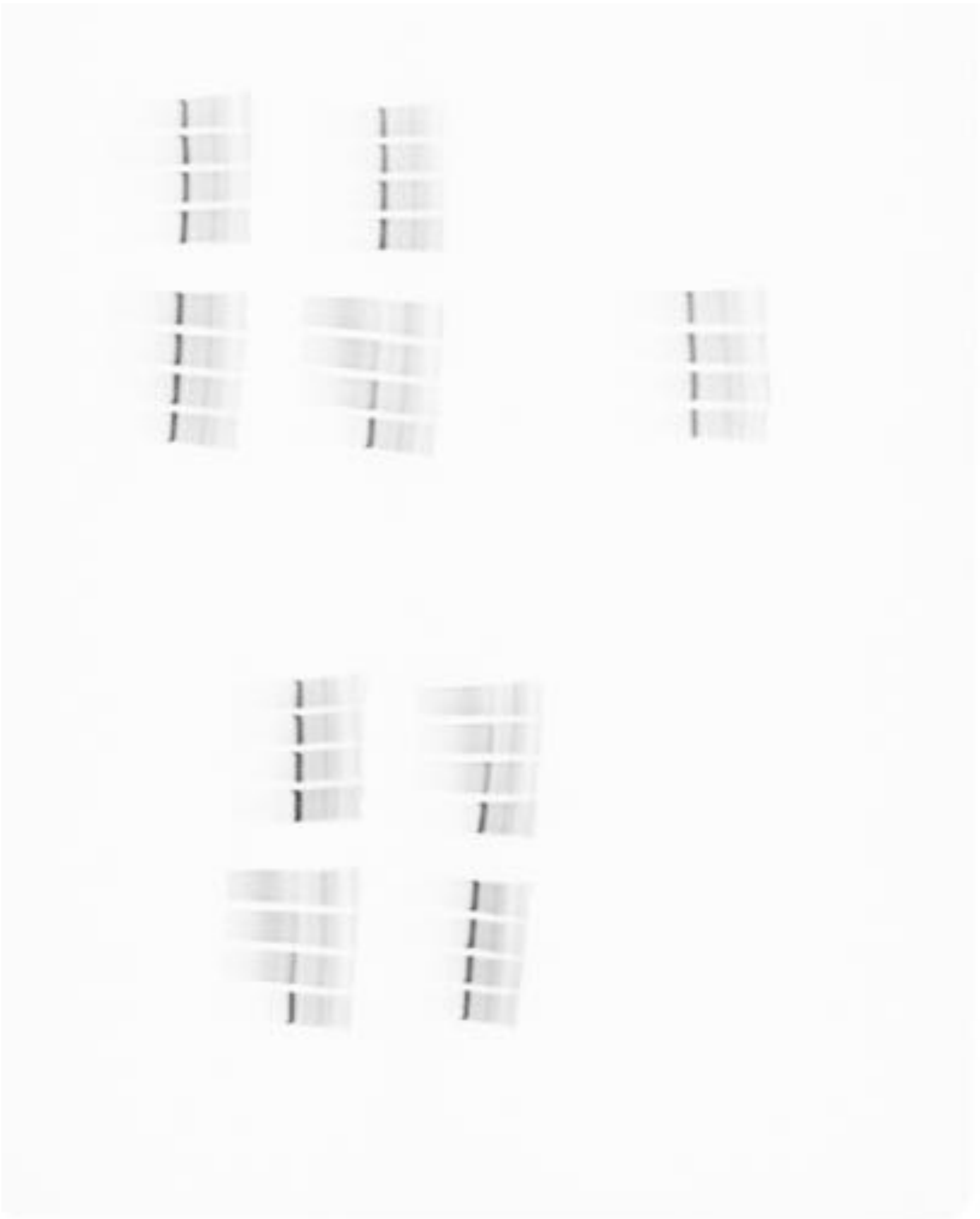

Fig 11D

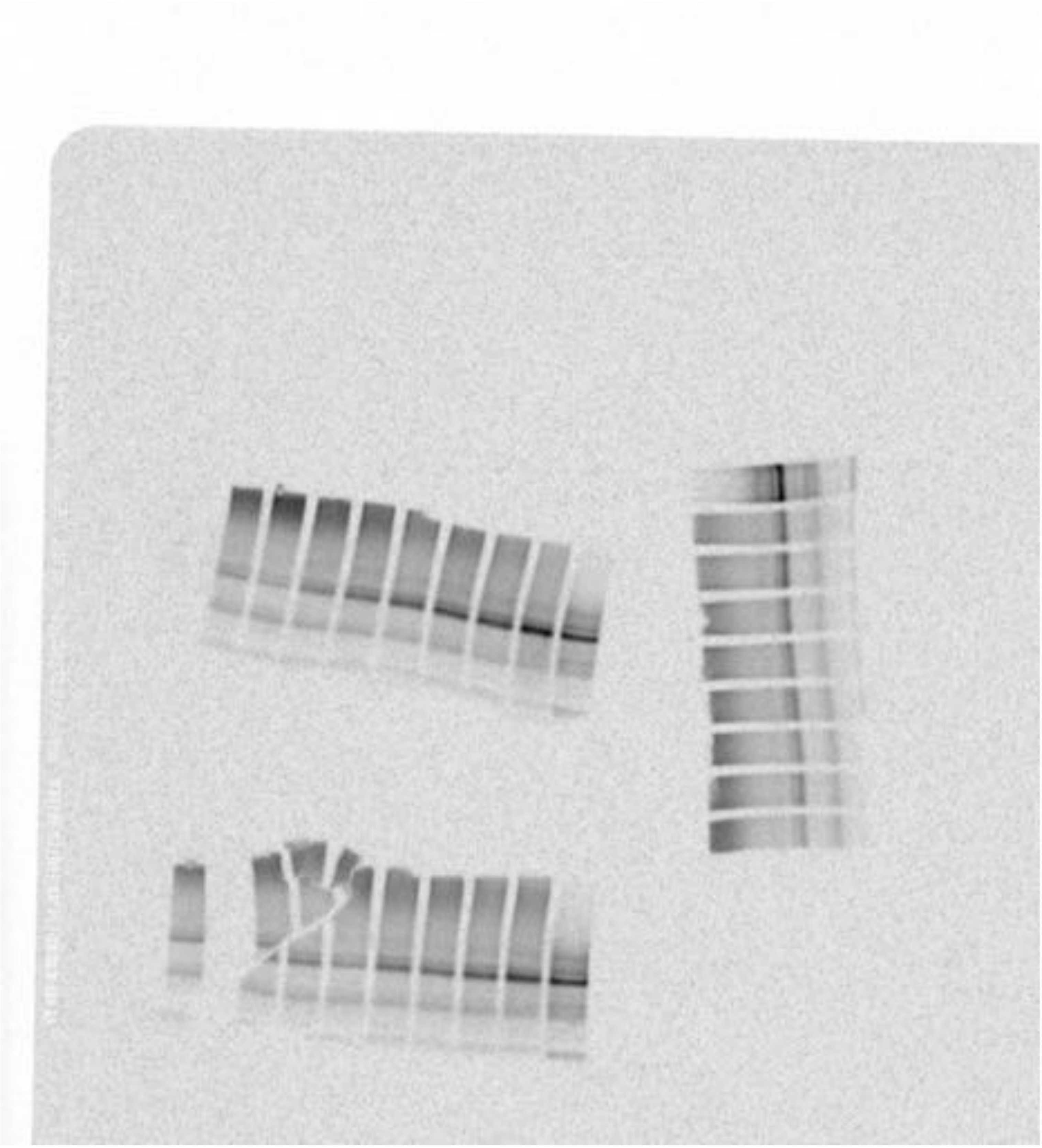

Fig 12A

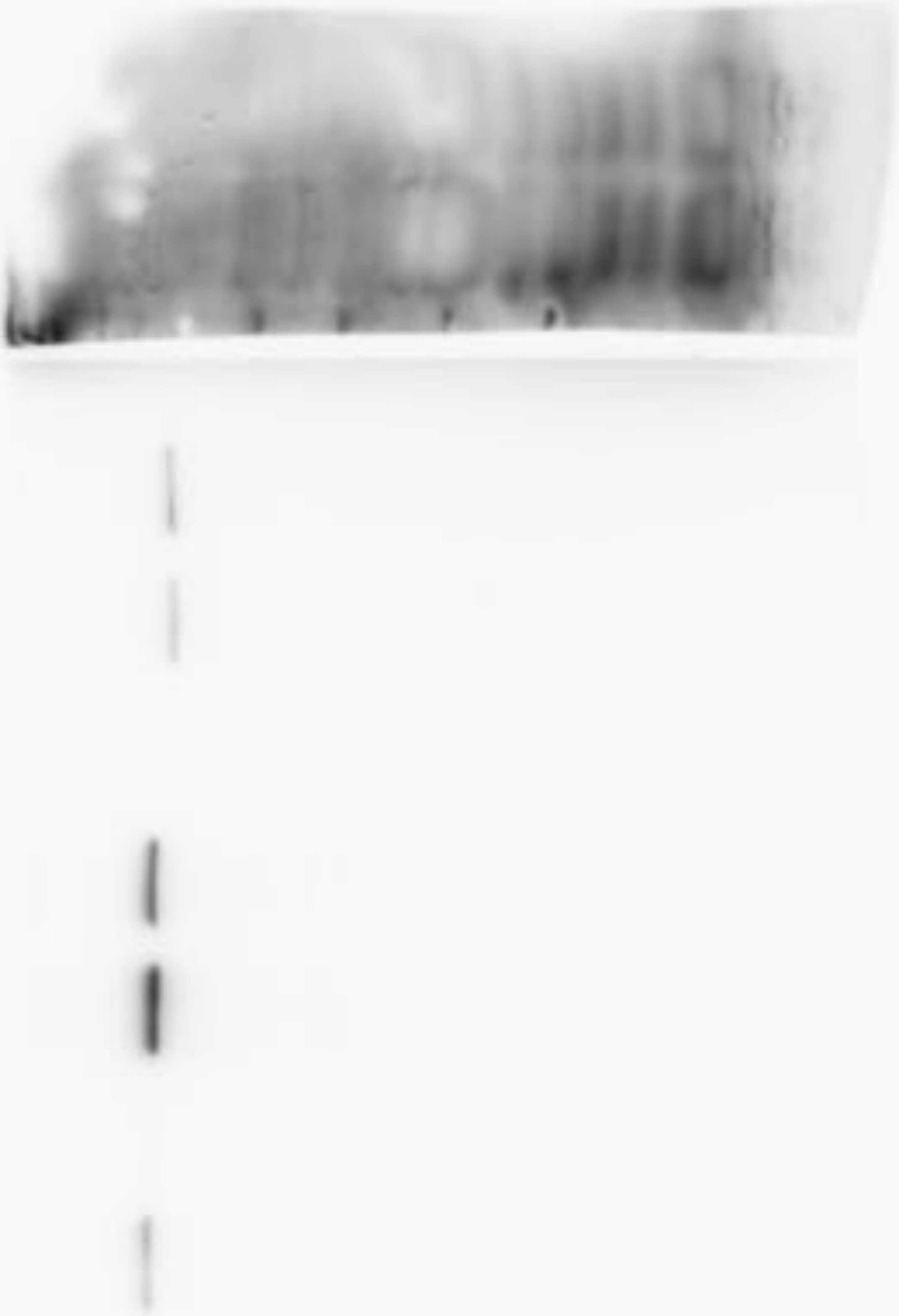

Fig 12B

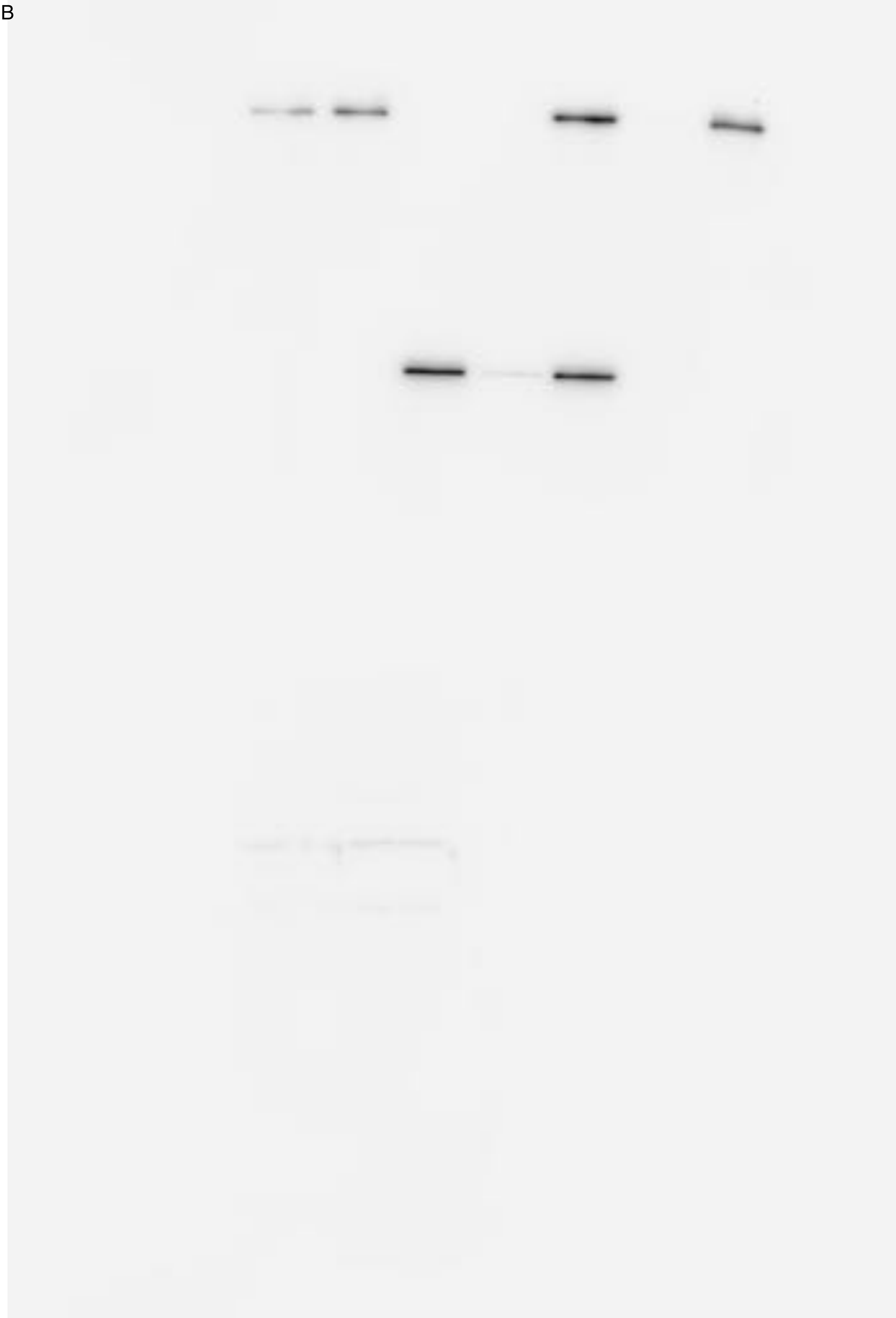

Fig 12B-1

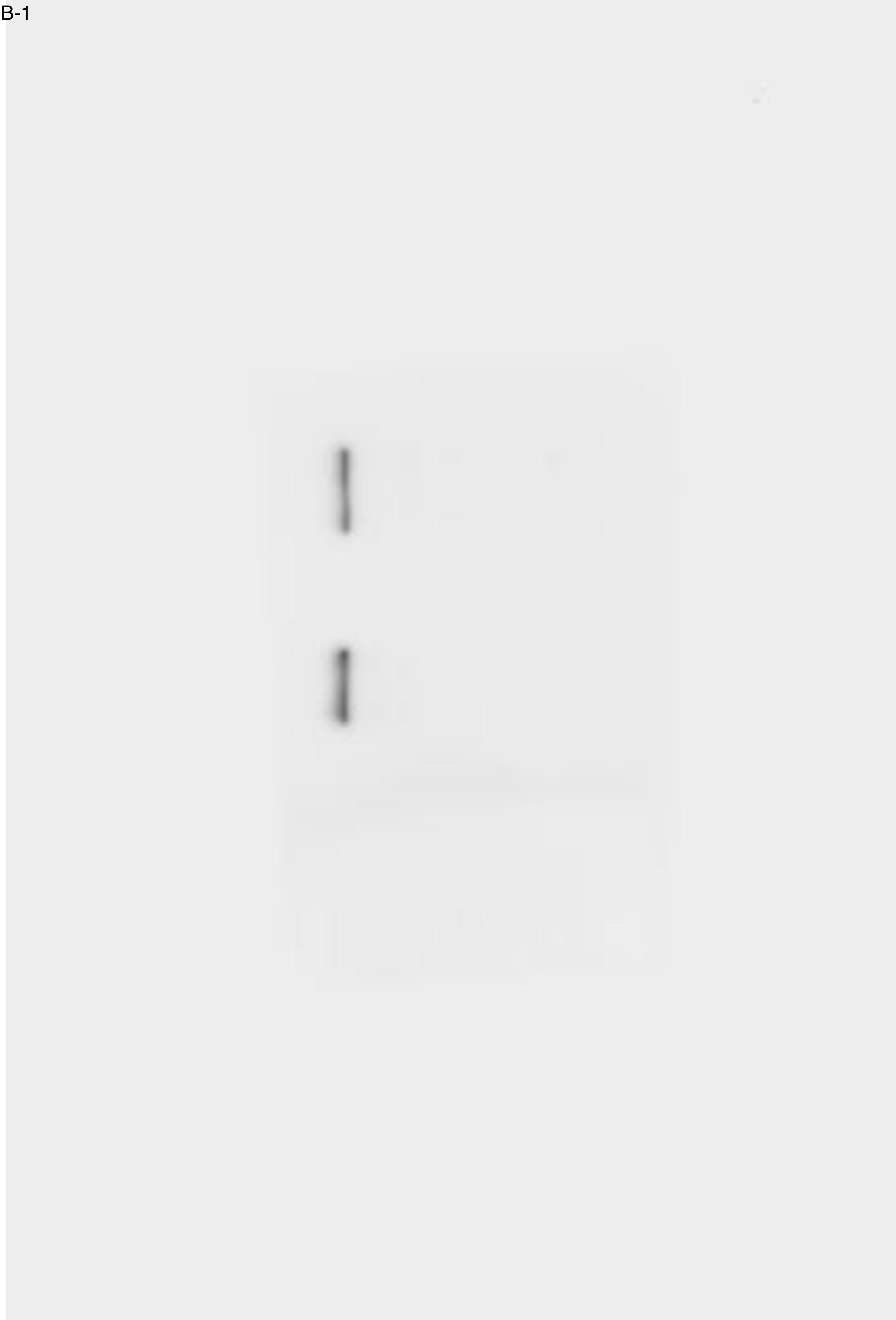

Fig 12C

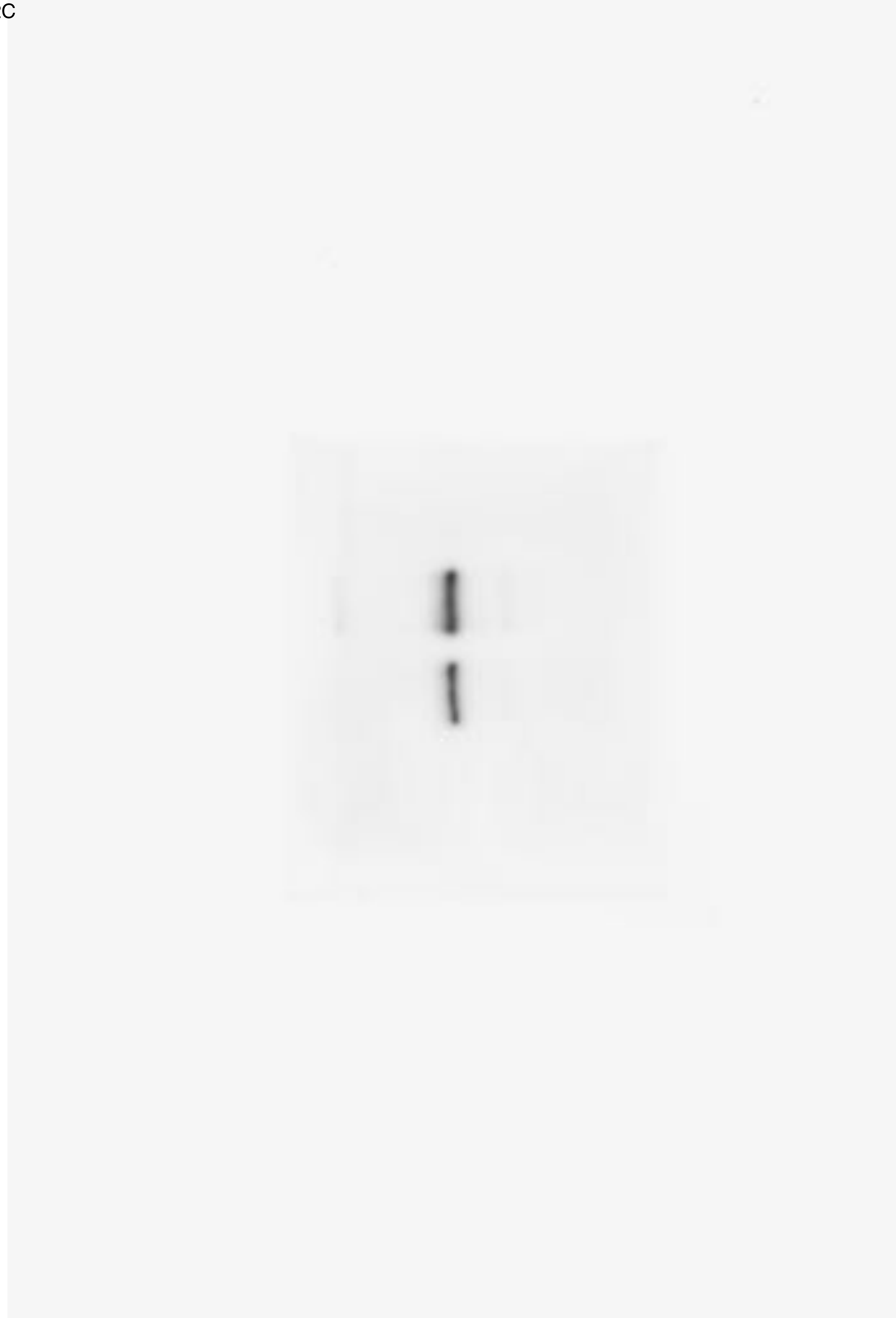

Fig 12D

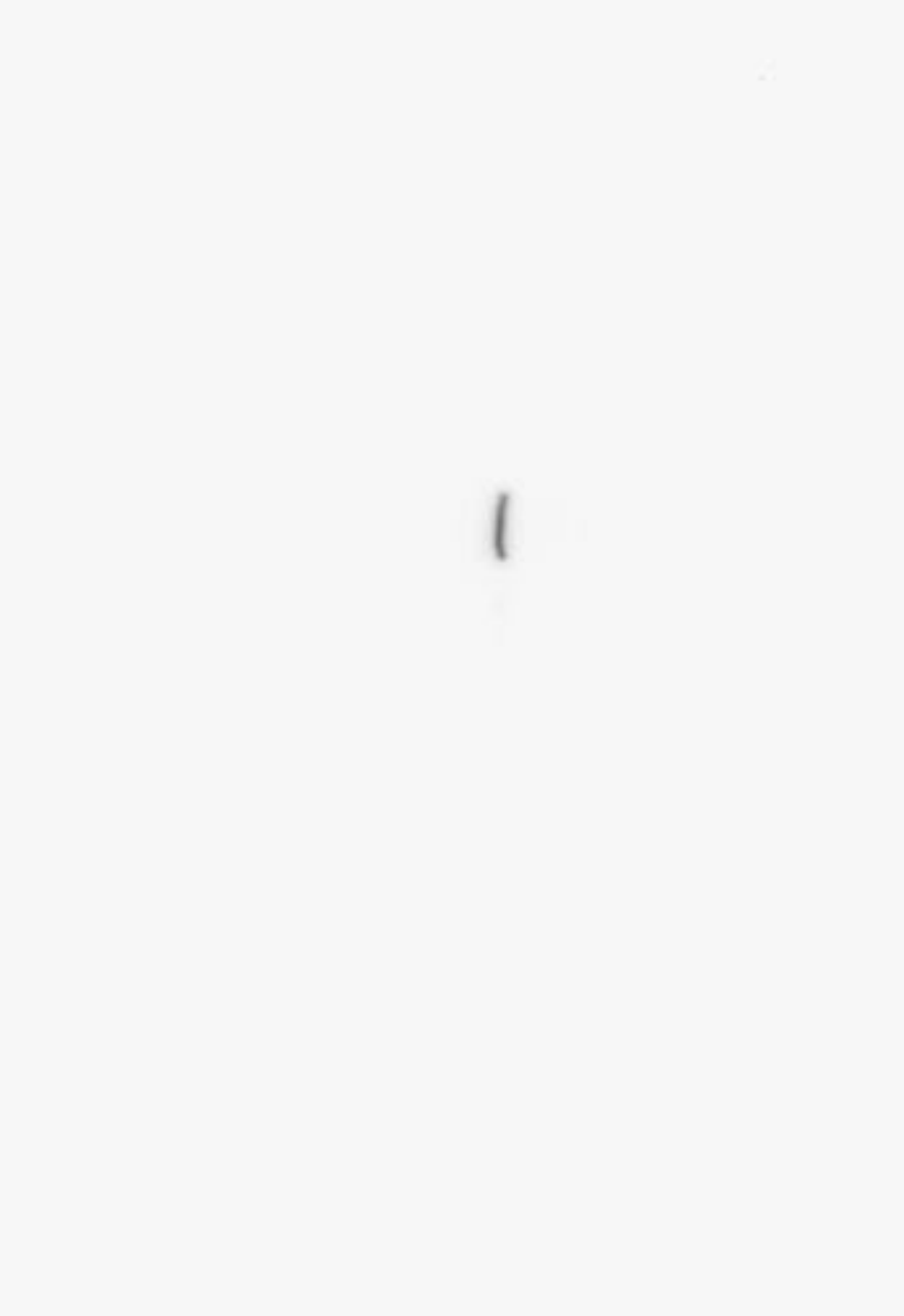

Fig 12E

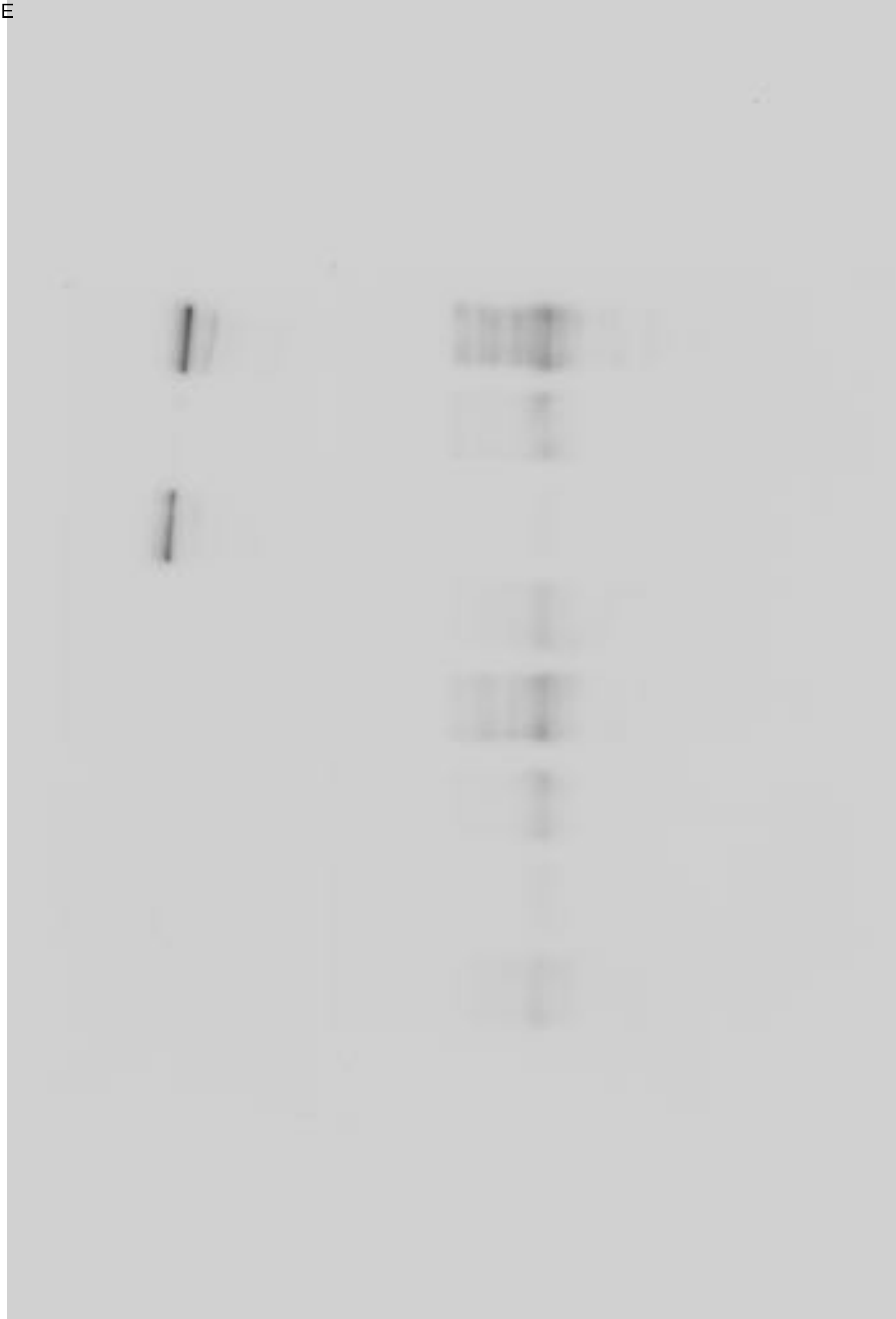

Fig 13B

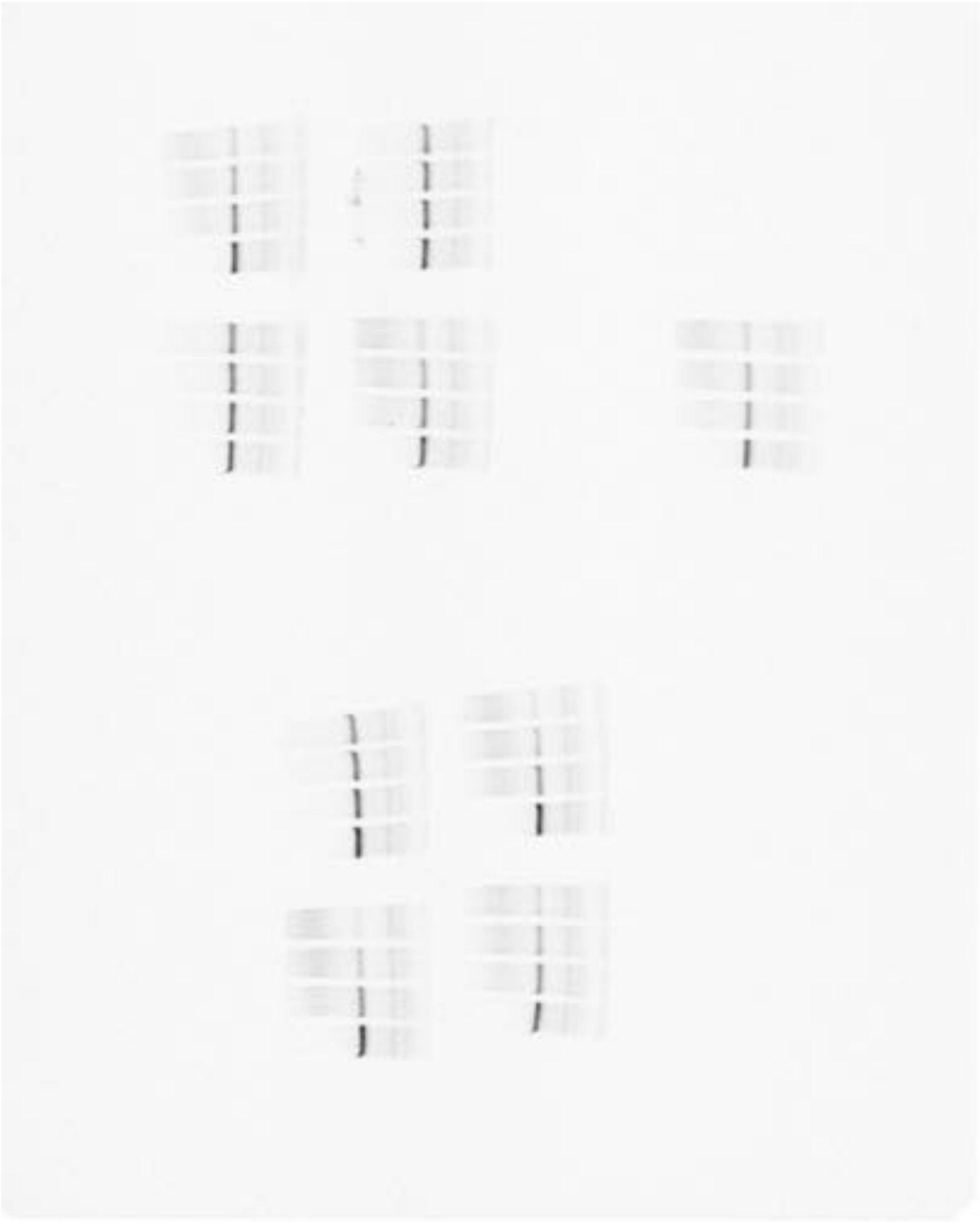

Fig 13C

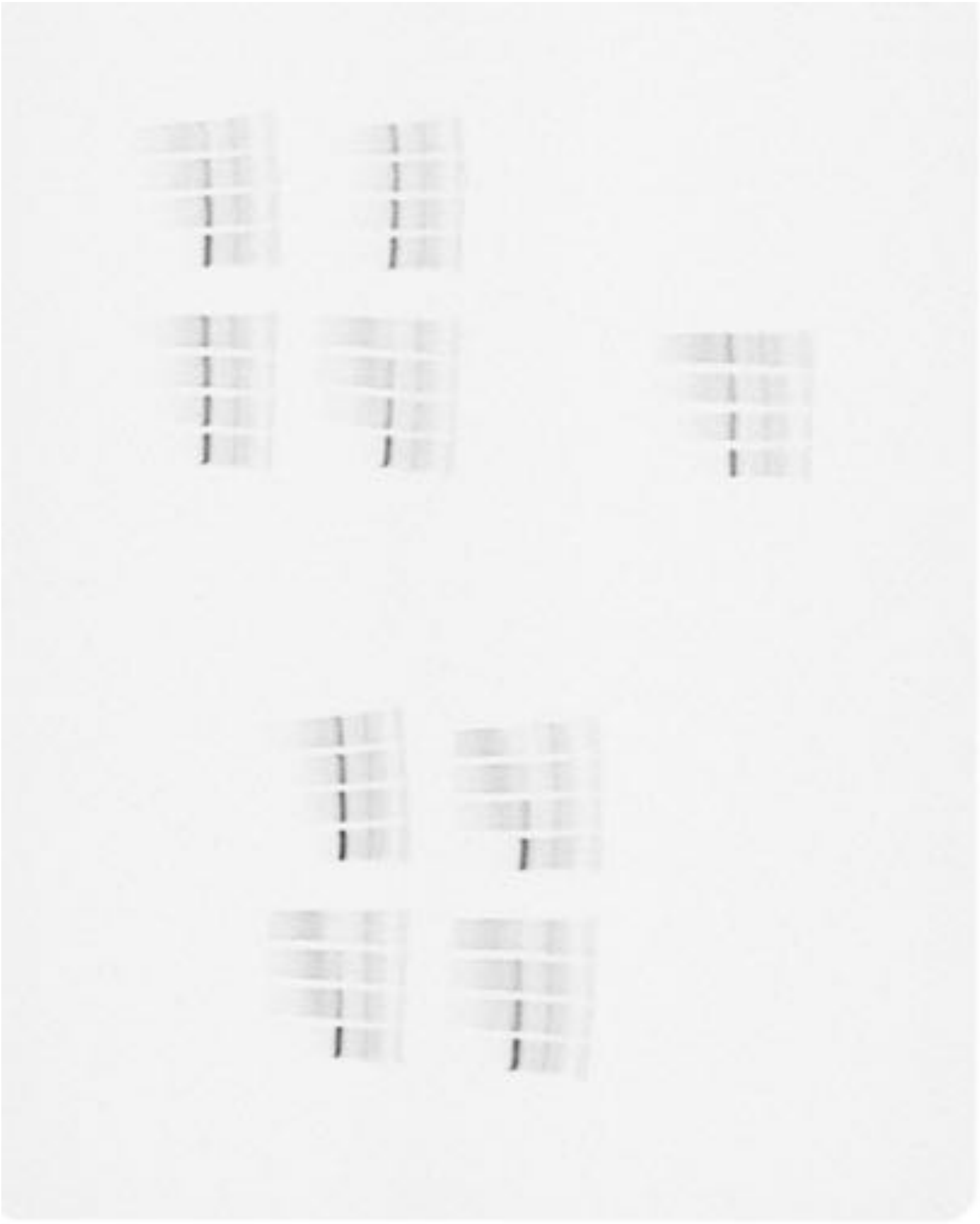

Fig 13D

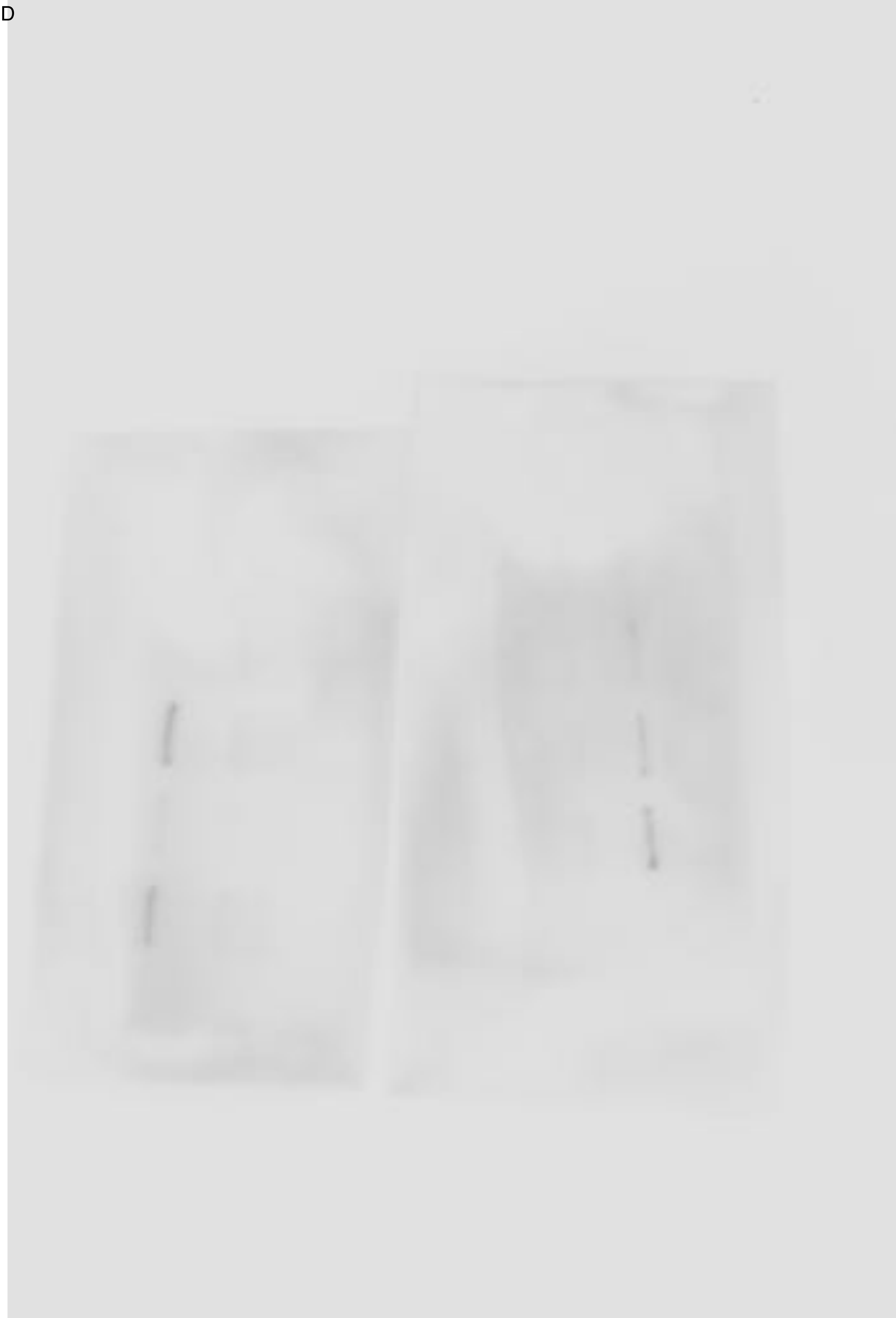

Fig 14A

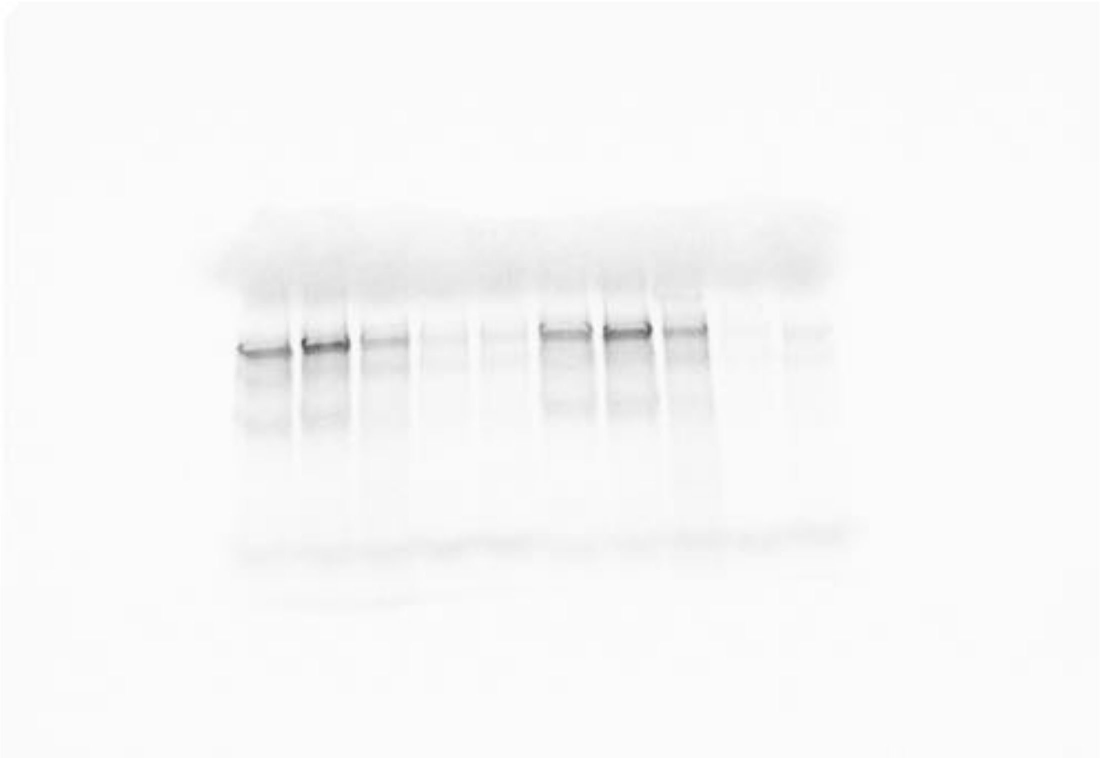

Fig 14A-1

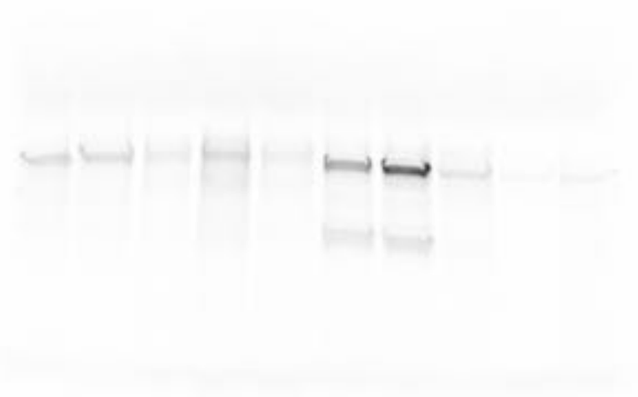

Fig S1A

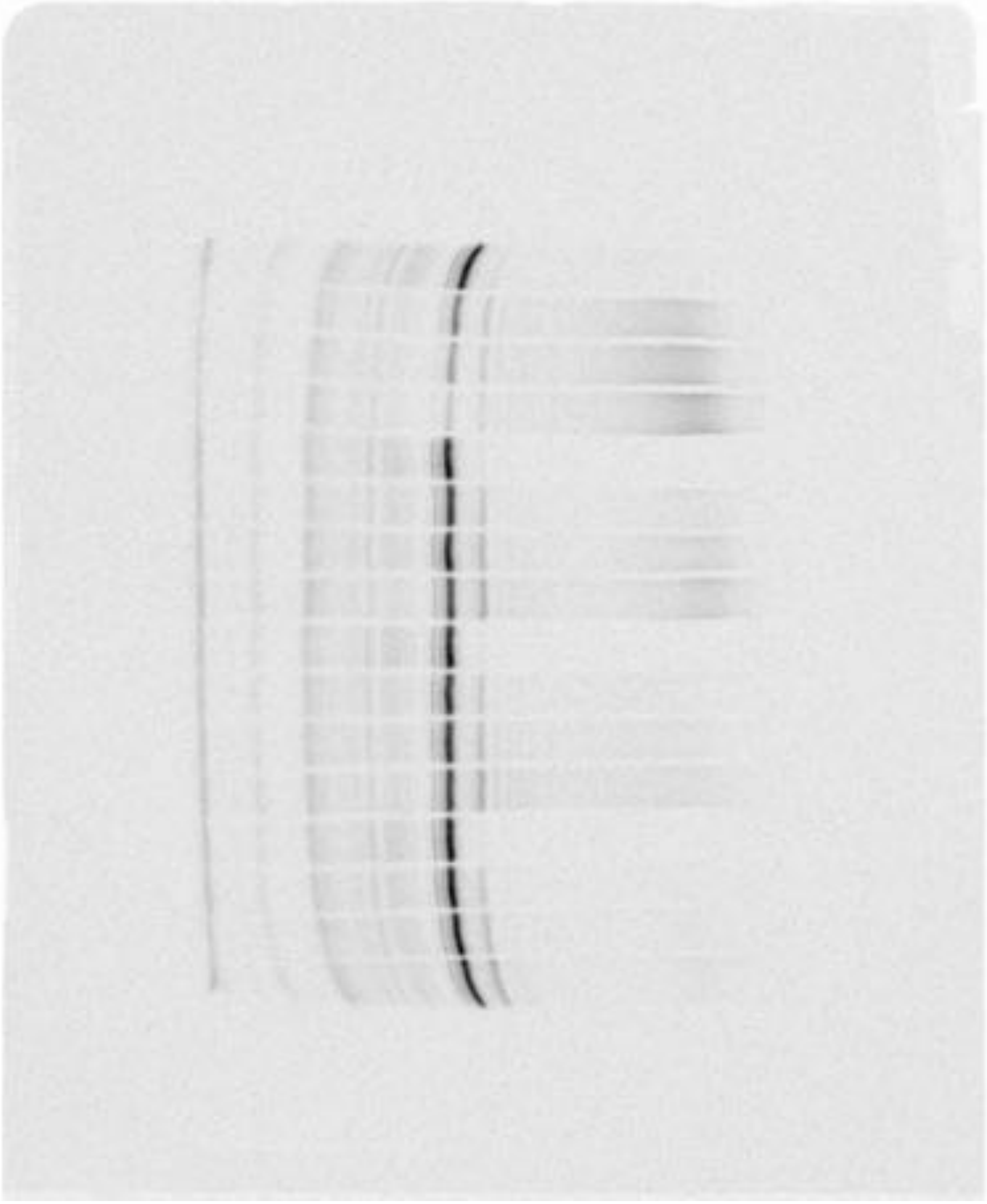

Fig S1B

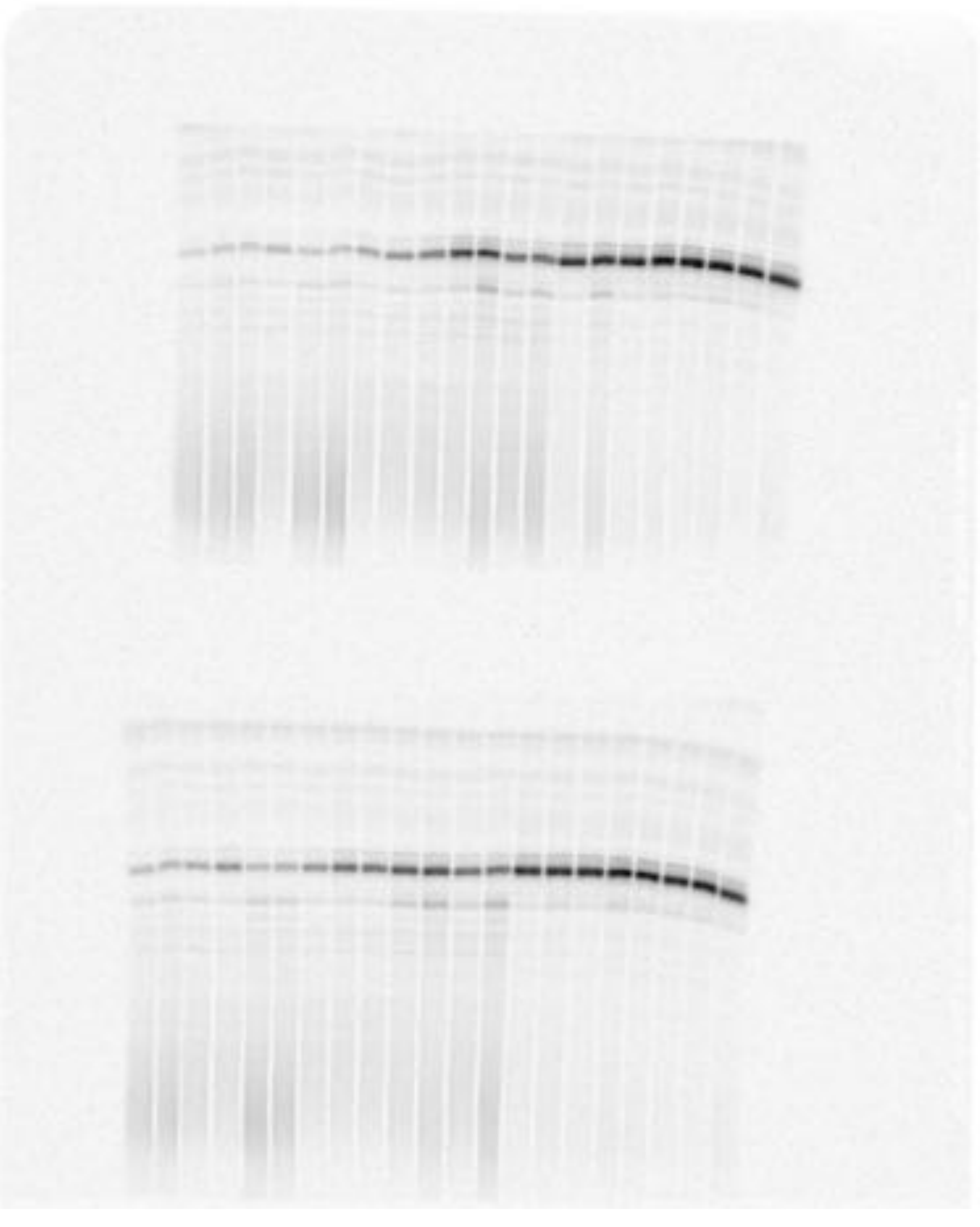

Fig S1B-1

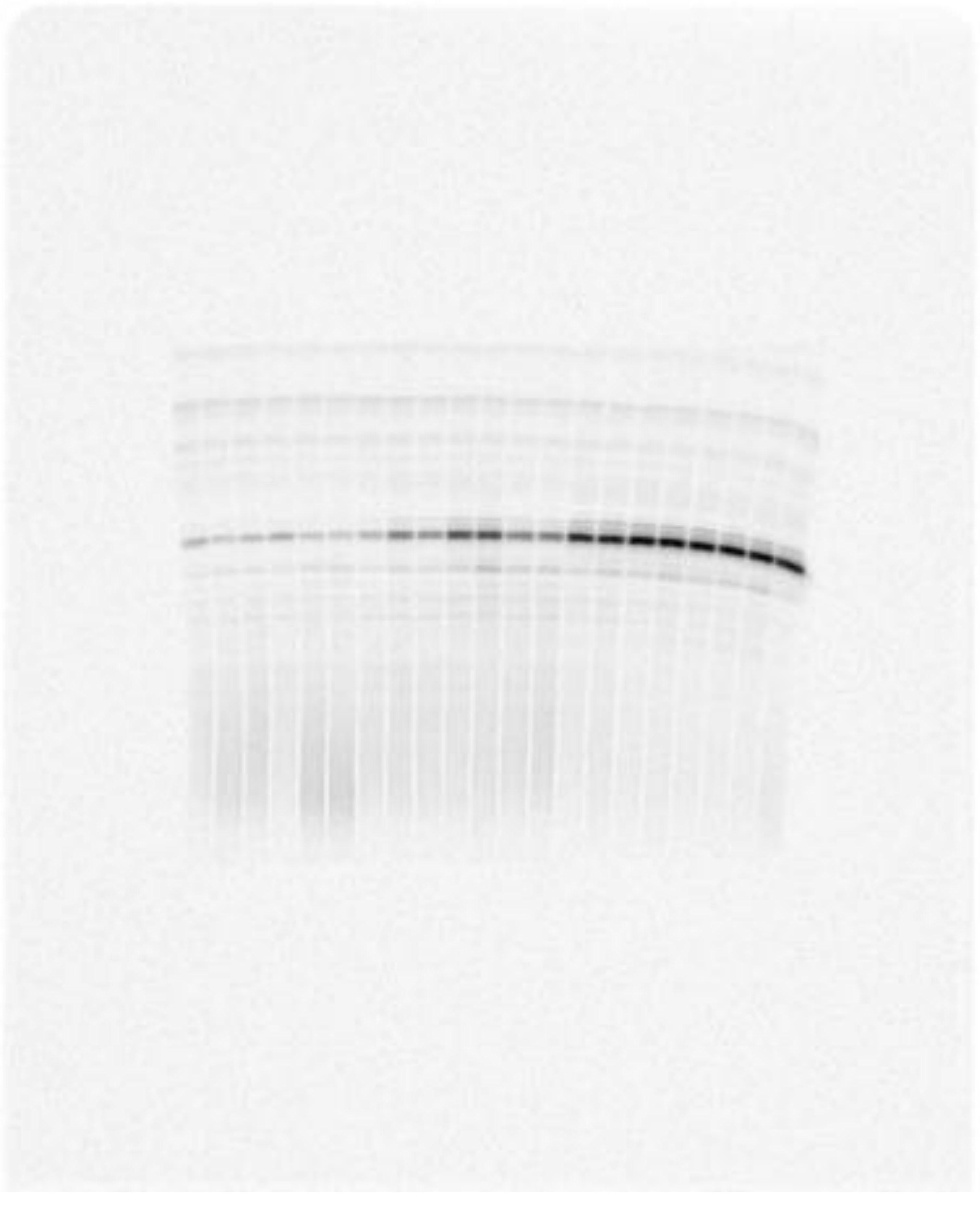

Fig S1C

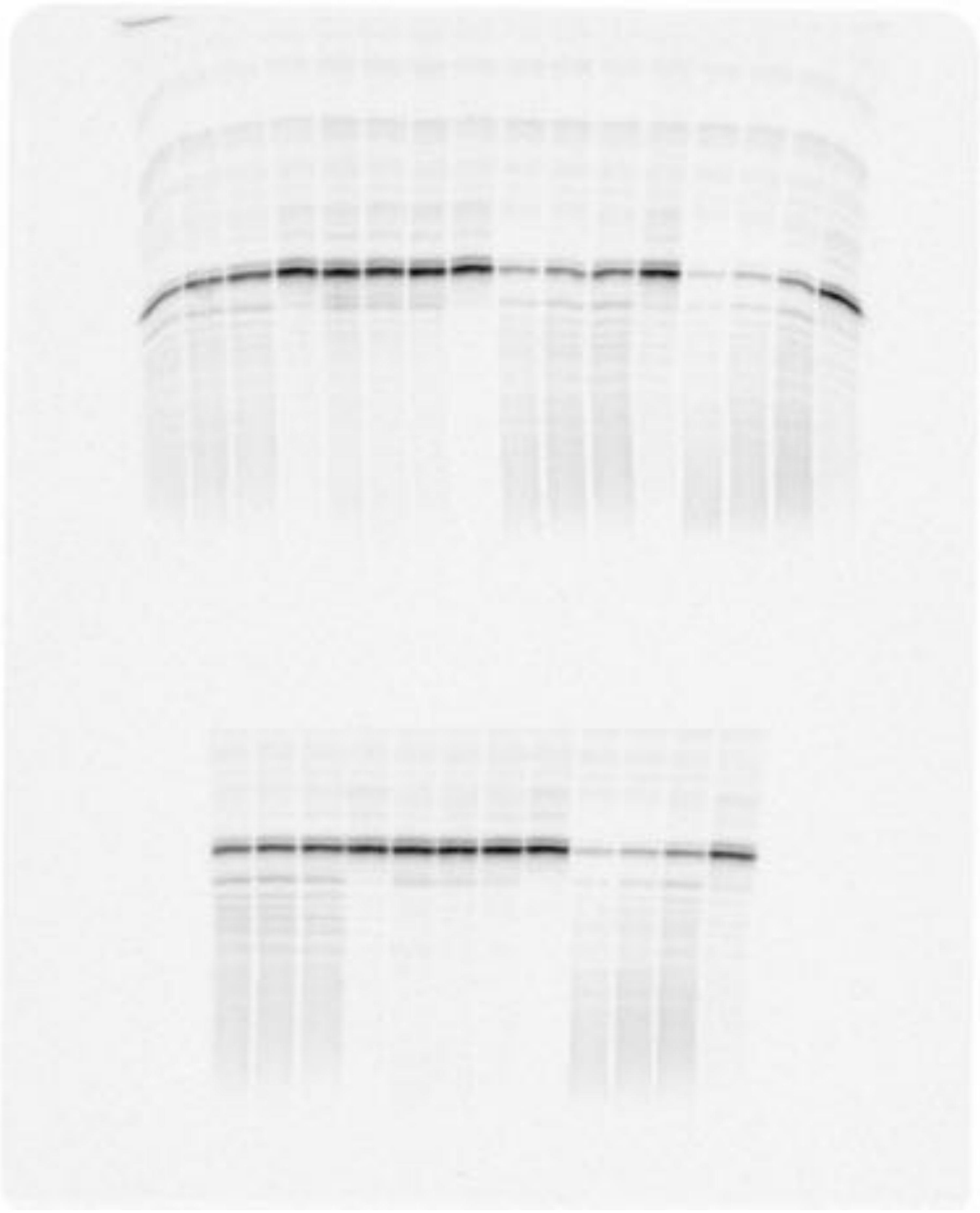

Fig S1D

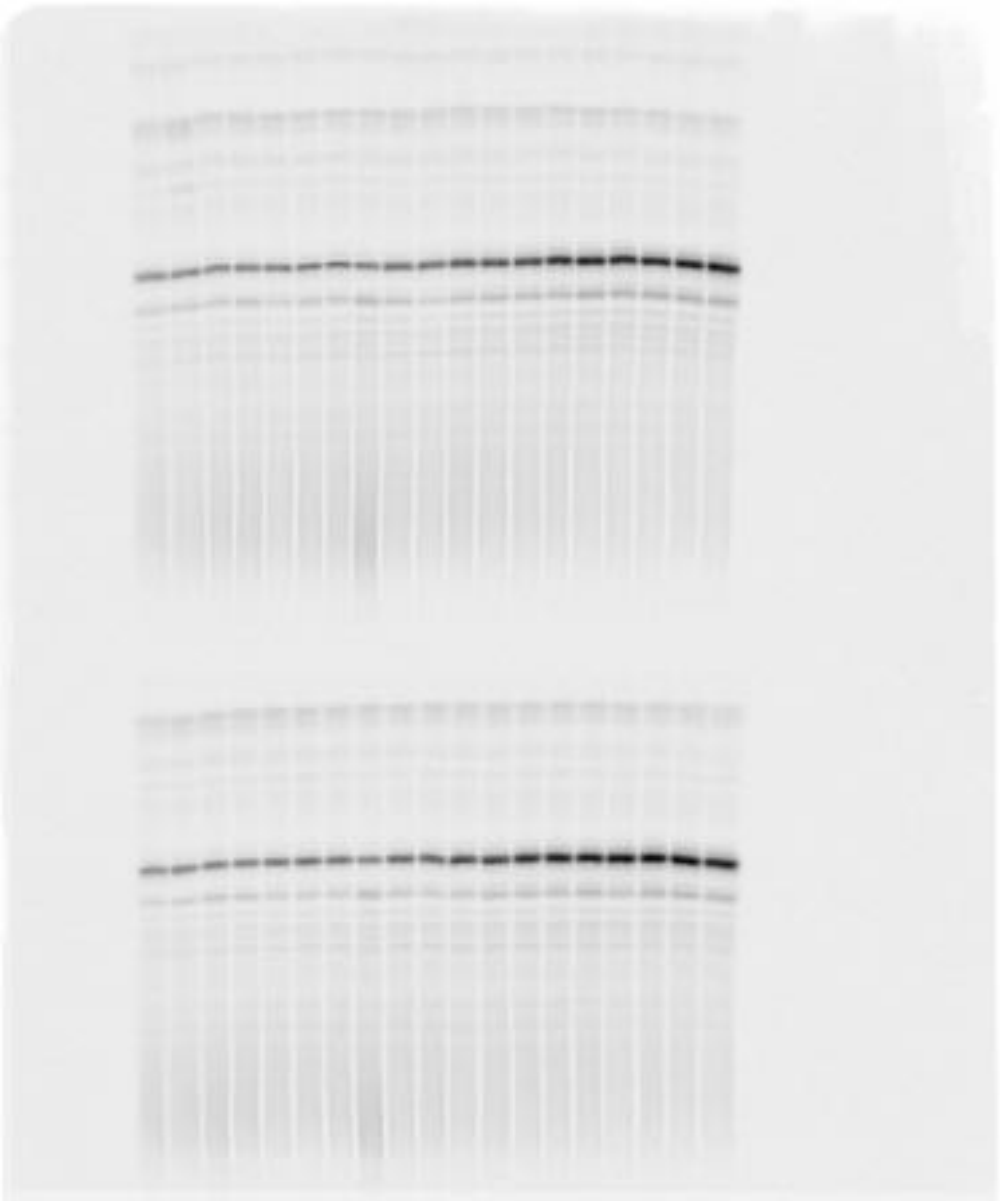

Fig S1D-1

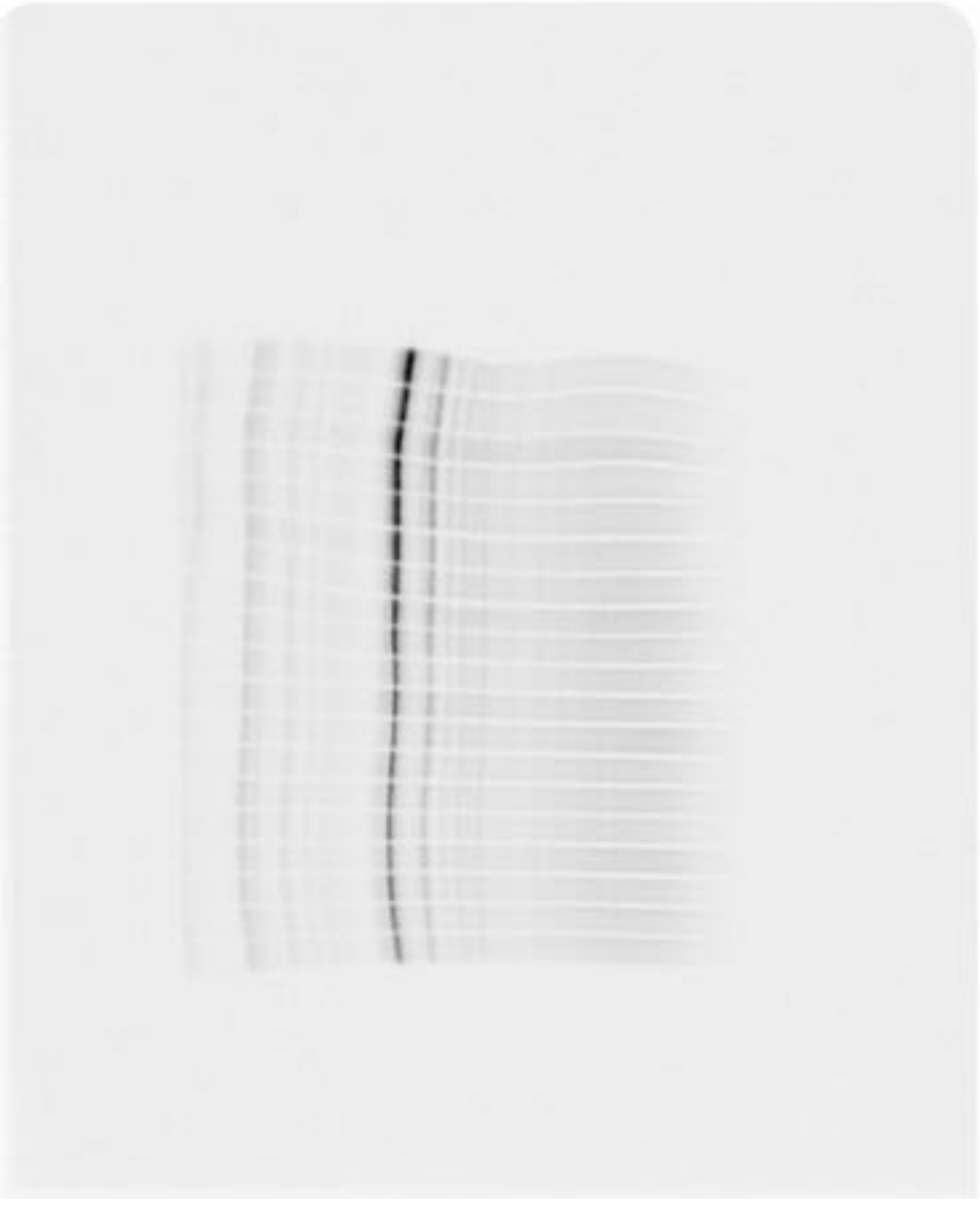

Fig S1E

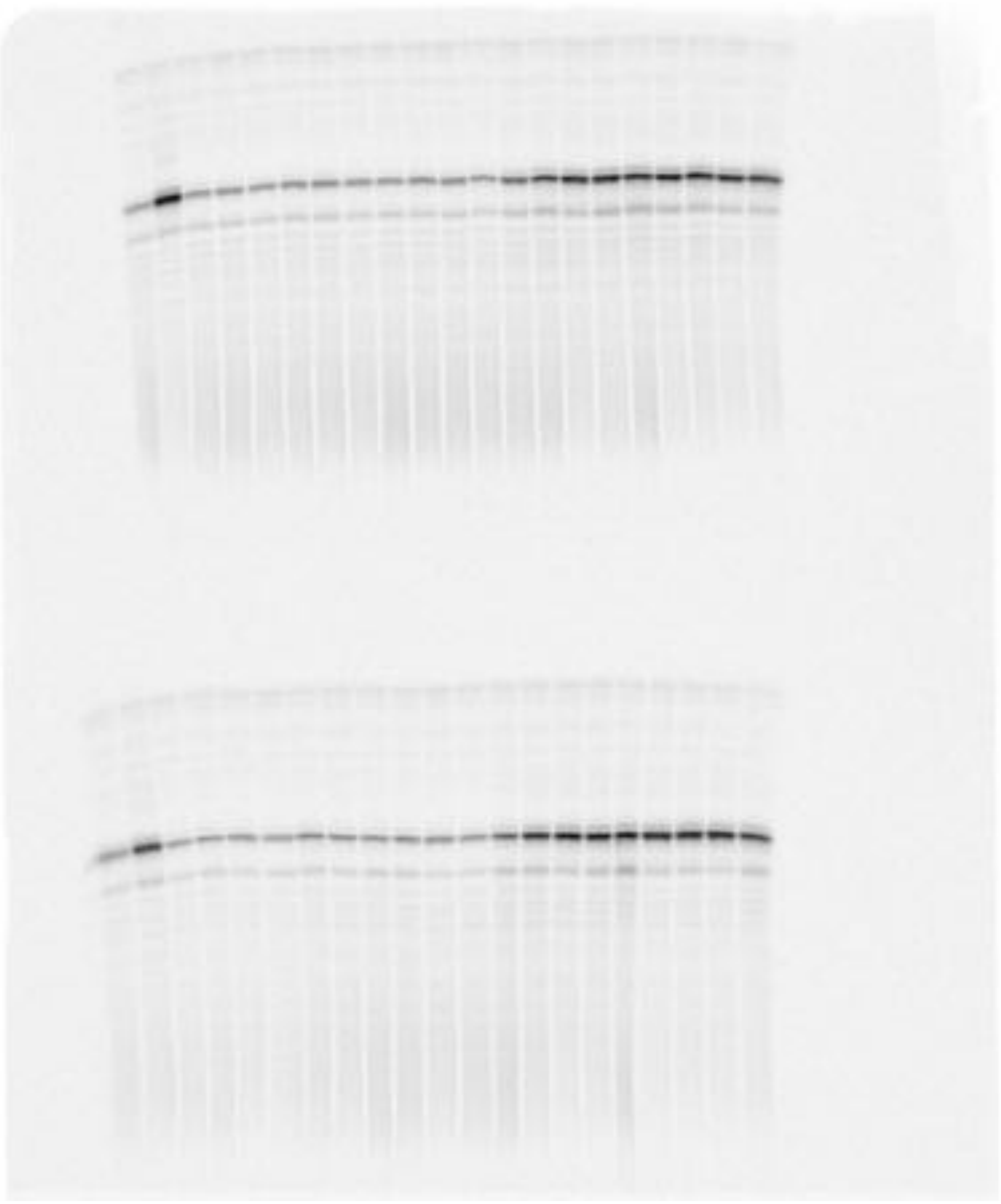

Fig S1E-1

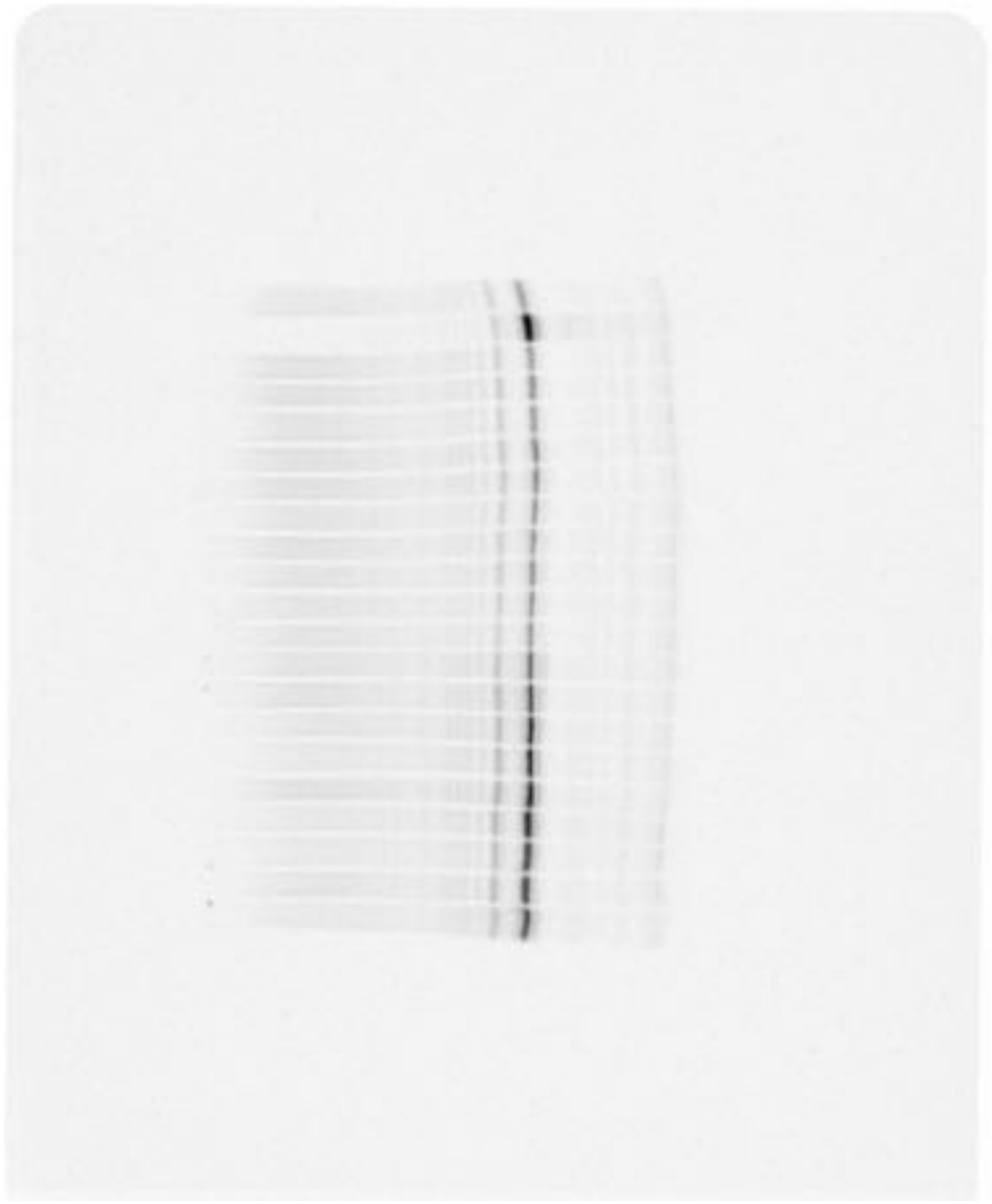

Fig S1F

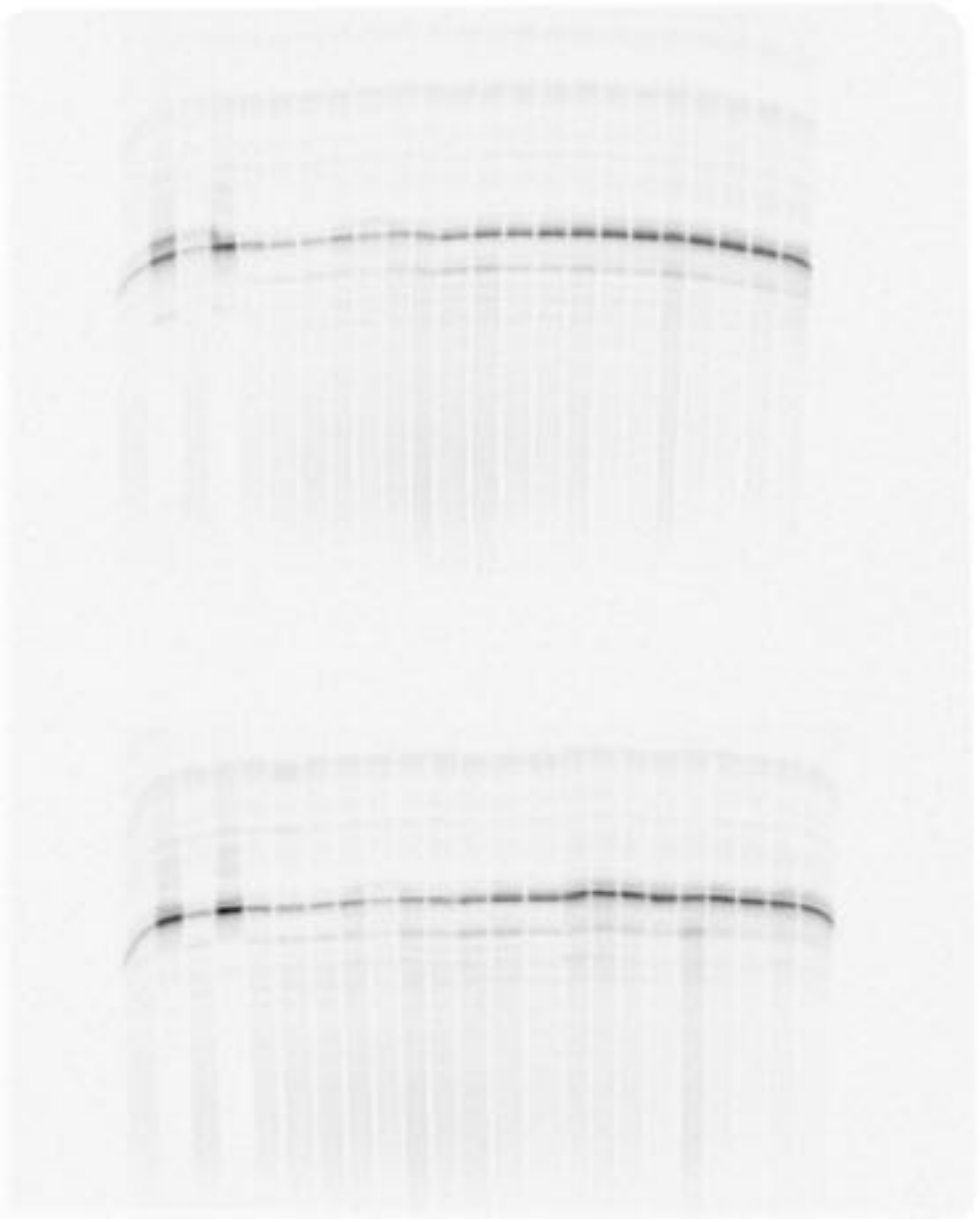

Fig S1F-1

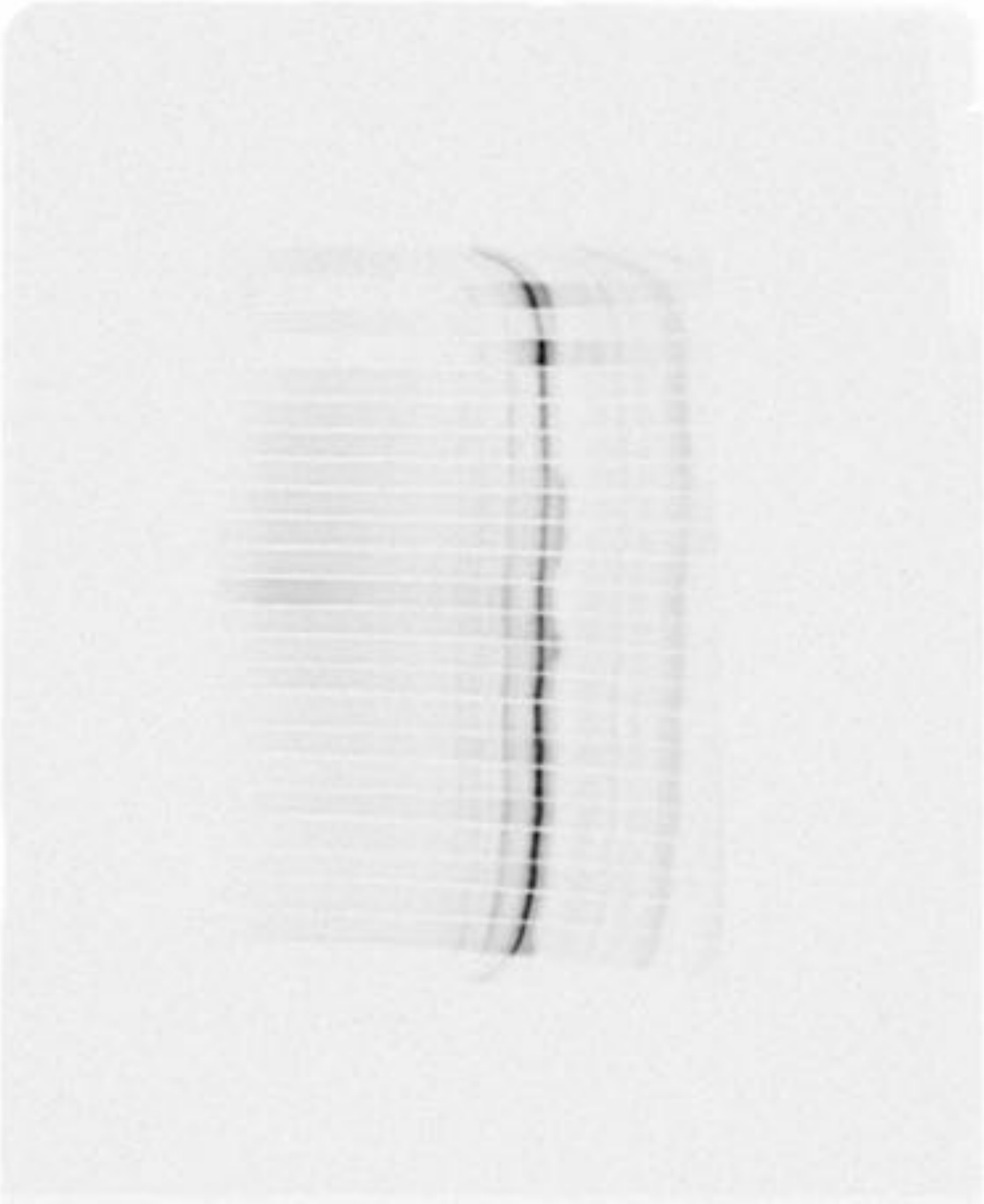

Fig S2A

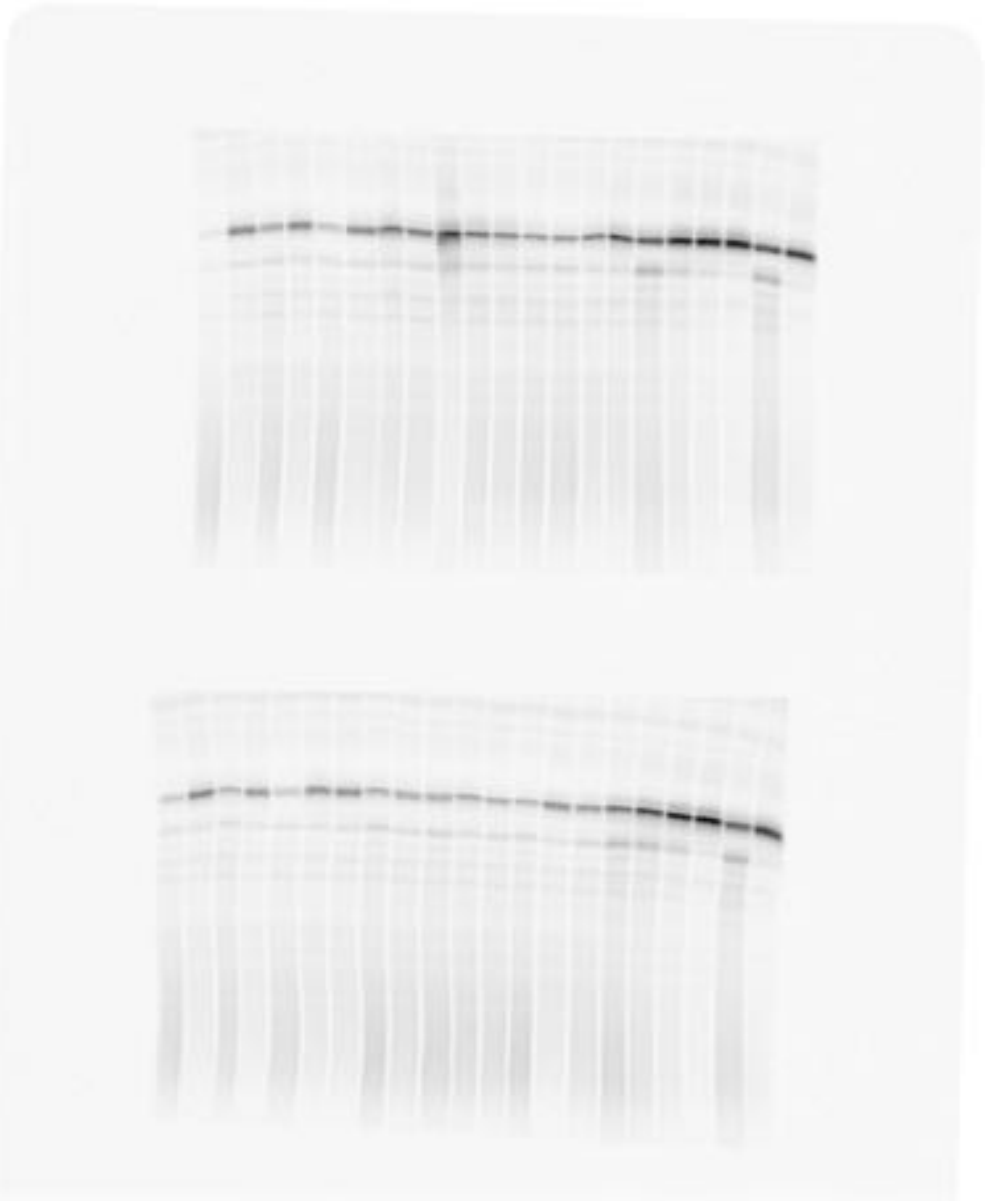

Fig S2A-1

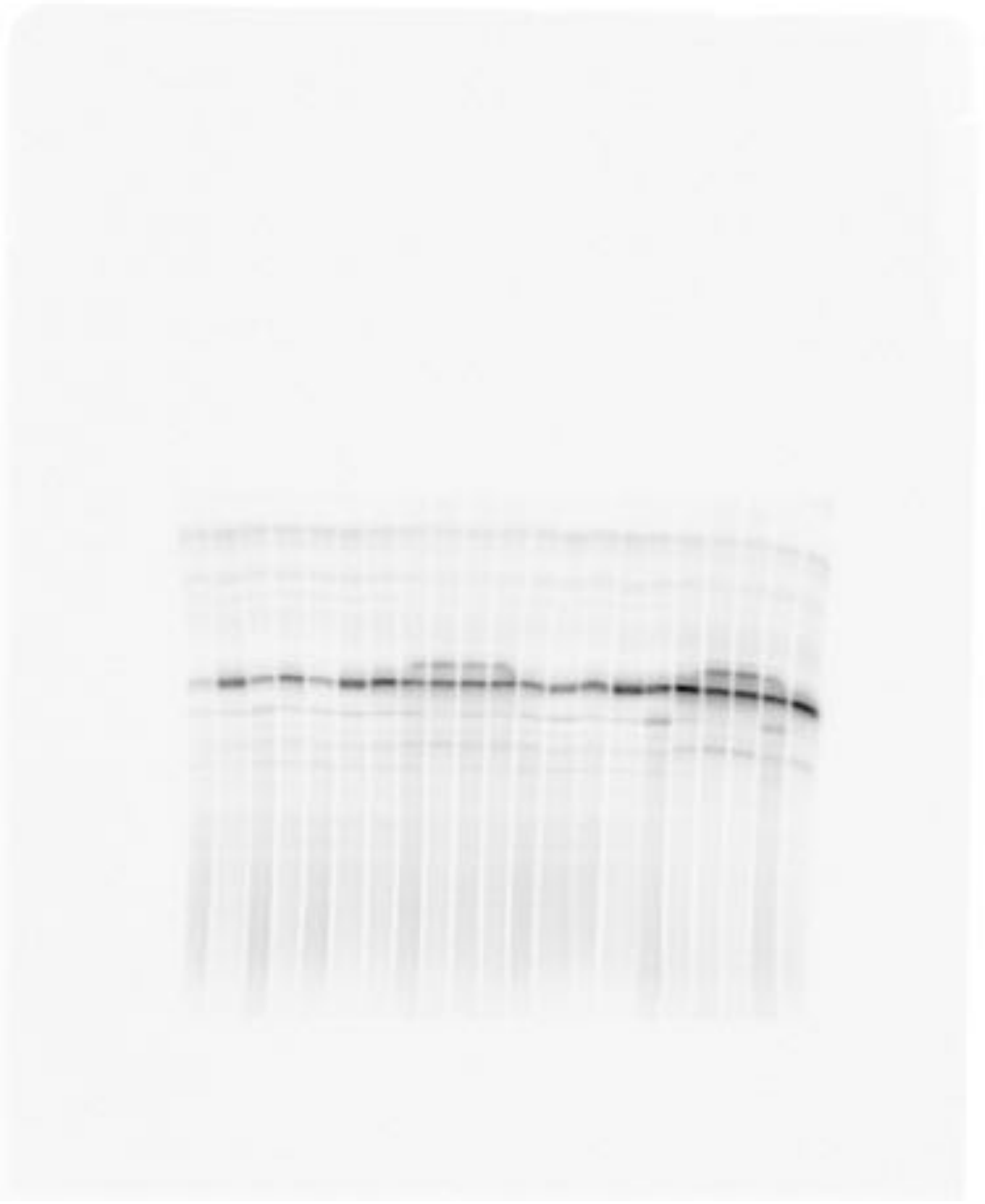

Fig S2B-1

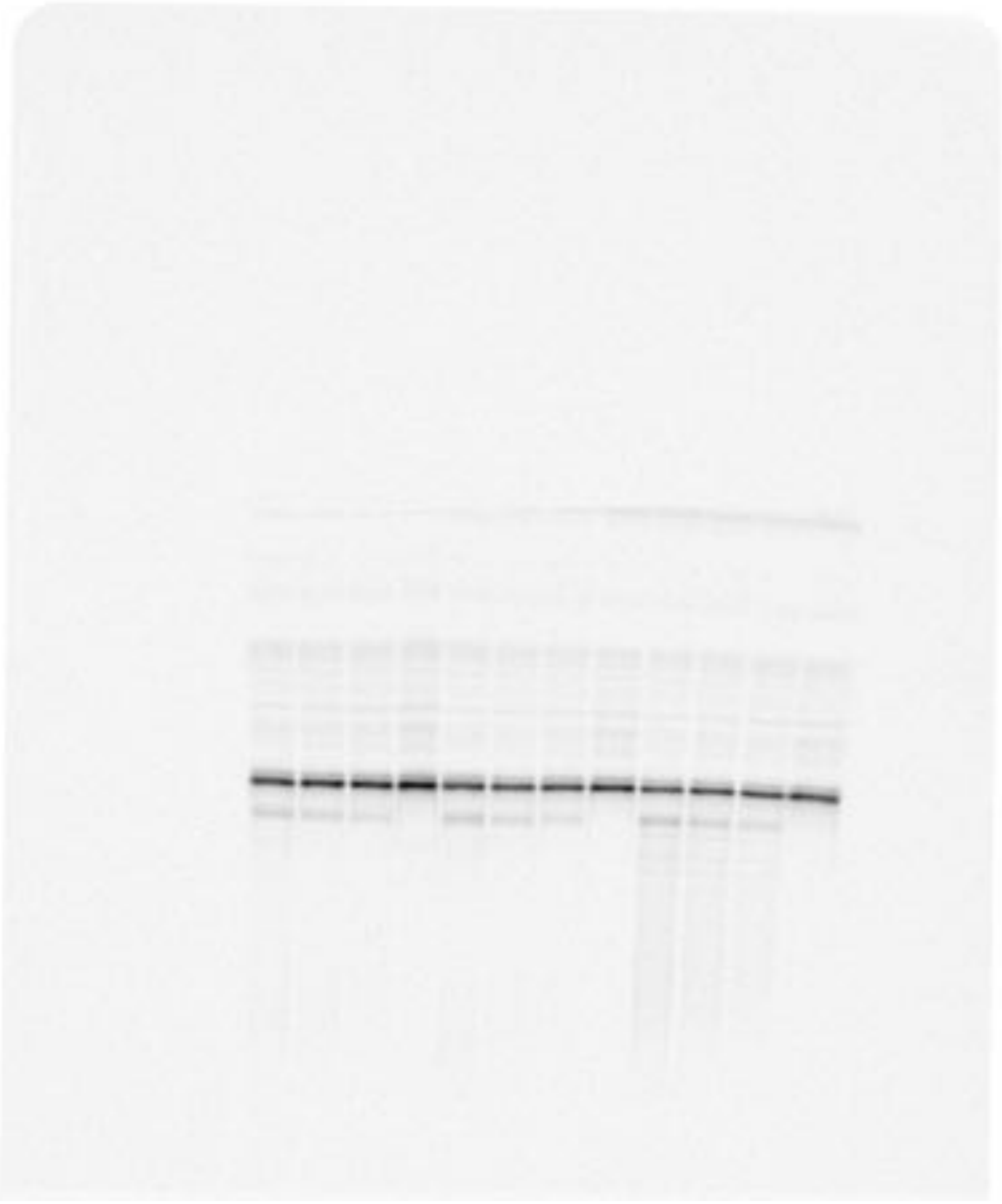

Fig S2B

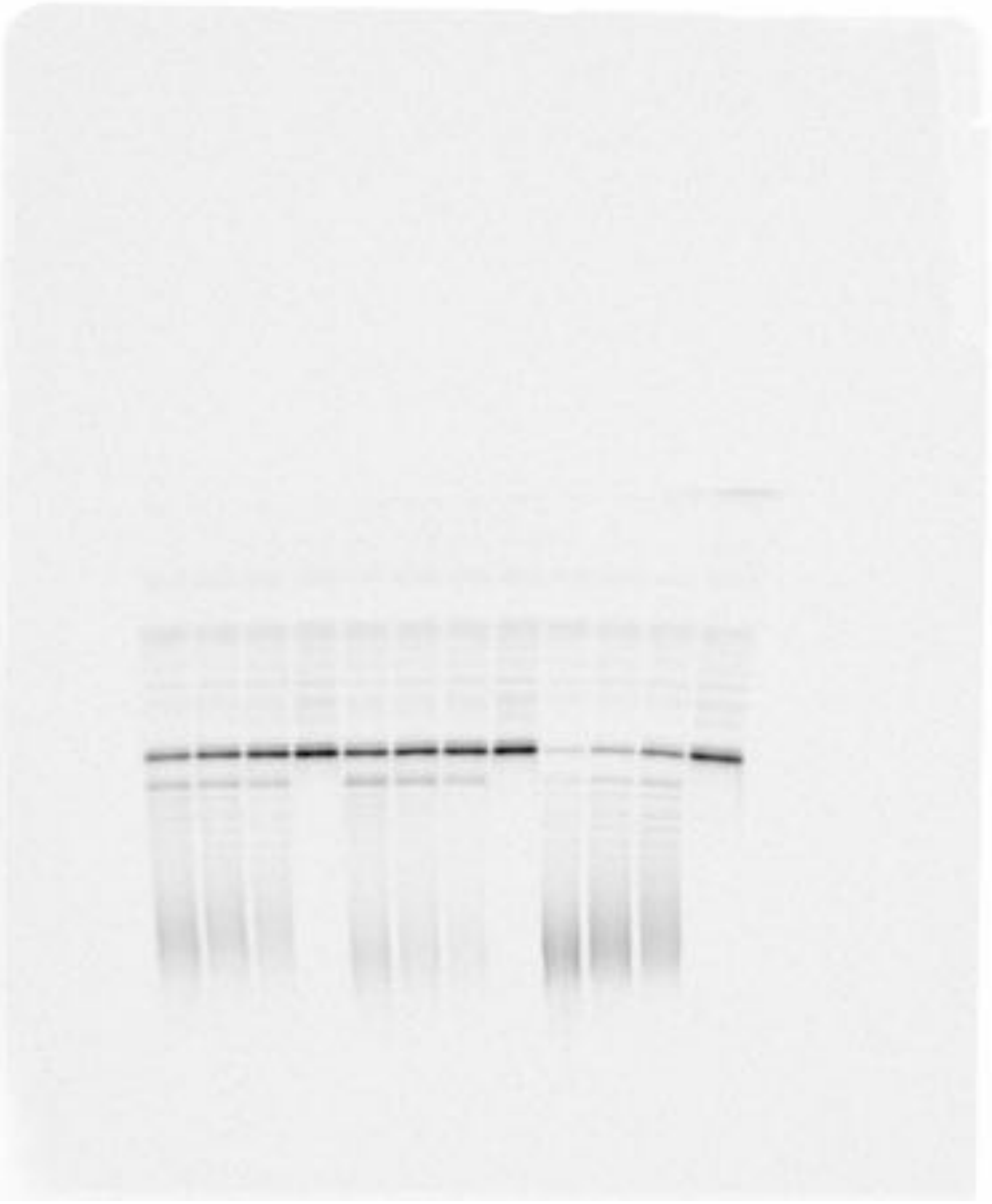

Fig S2D

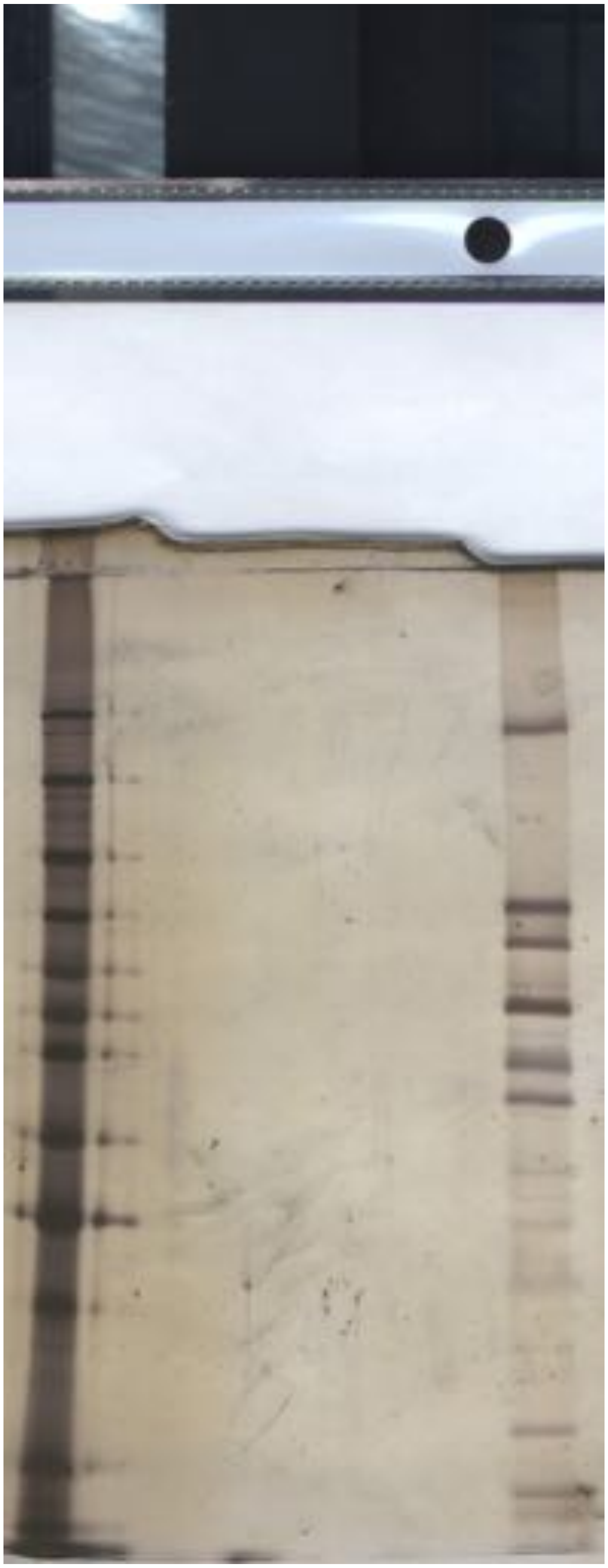

Fig S2D-1

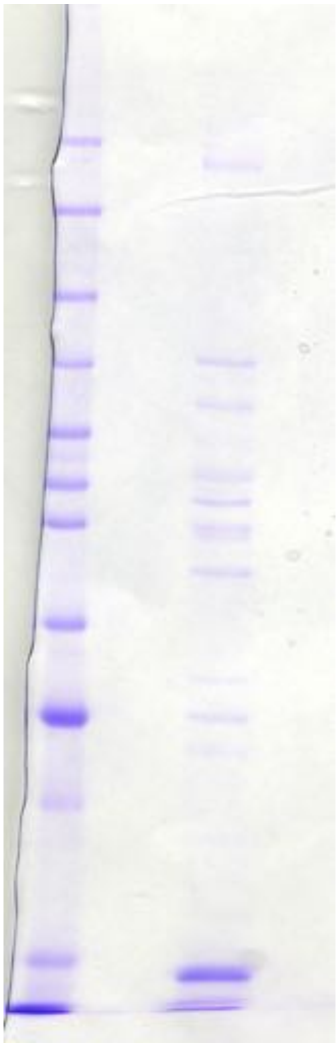

Fig S3AC

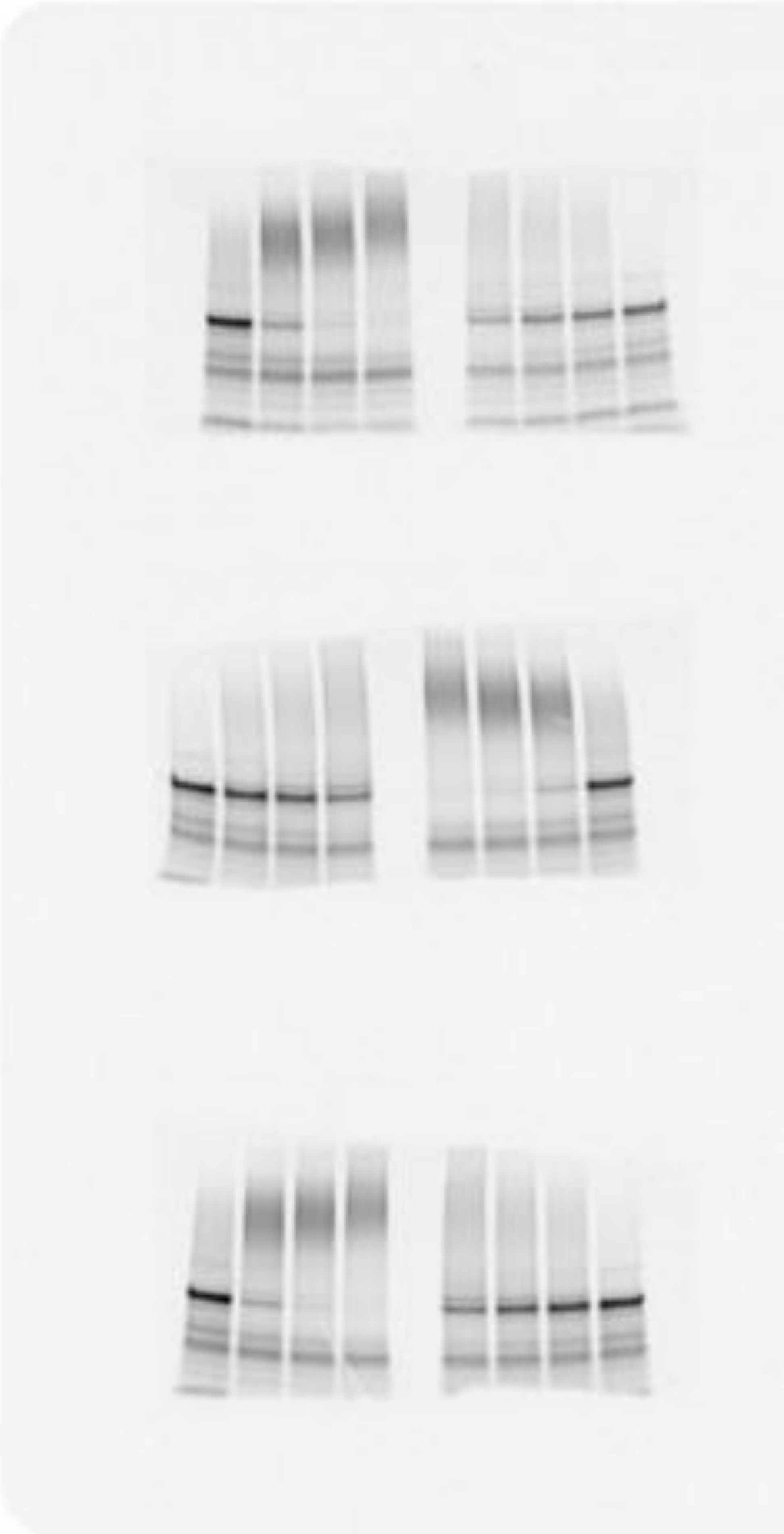

Fig S3E

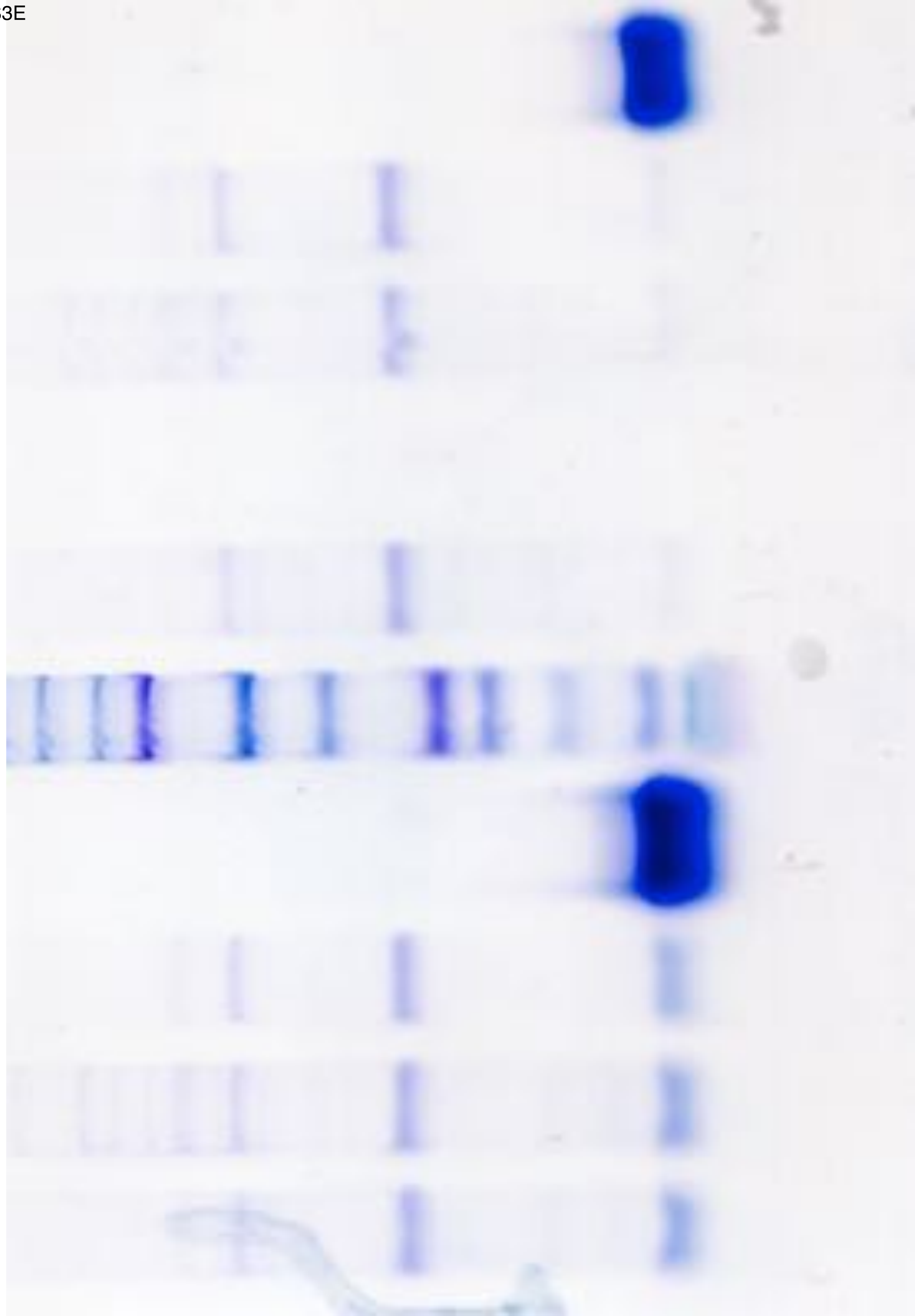

Fig S4A

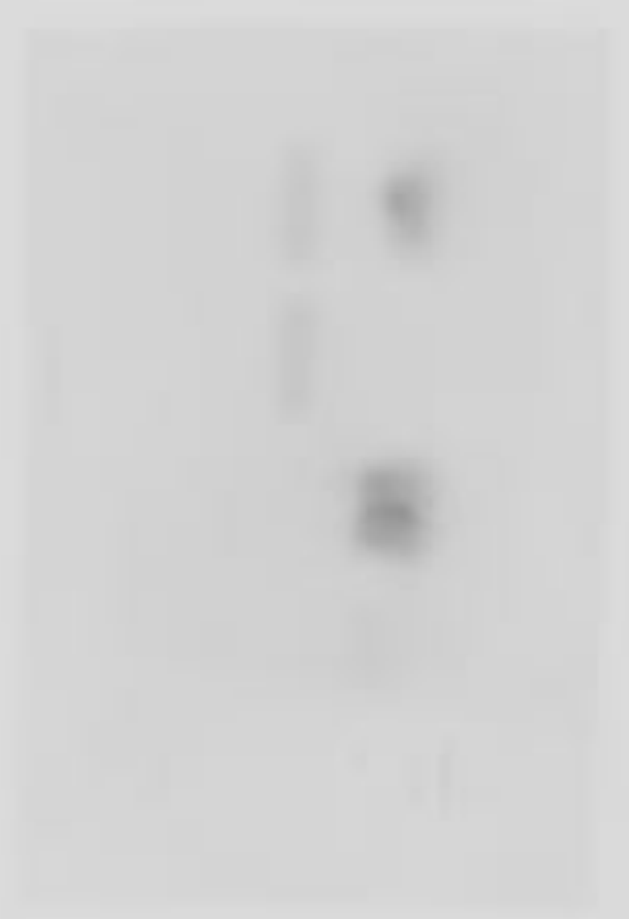

Fig S4A-1

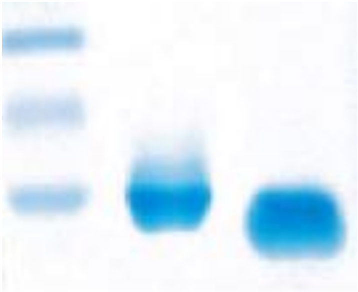

Fig S4B

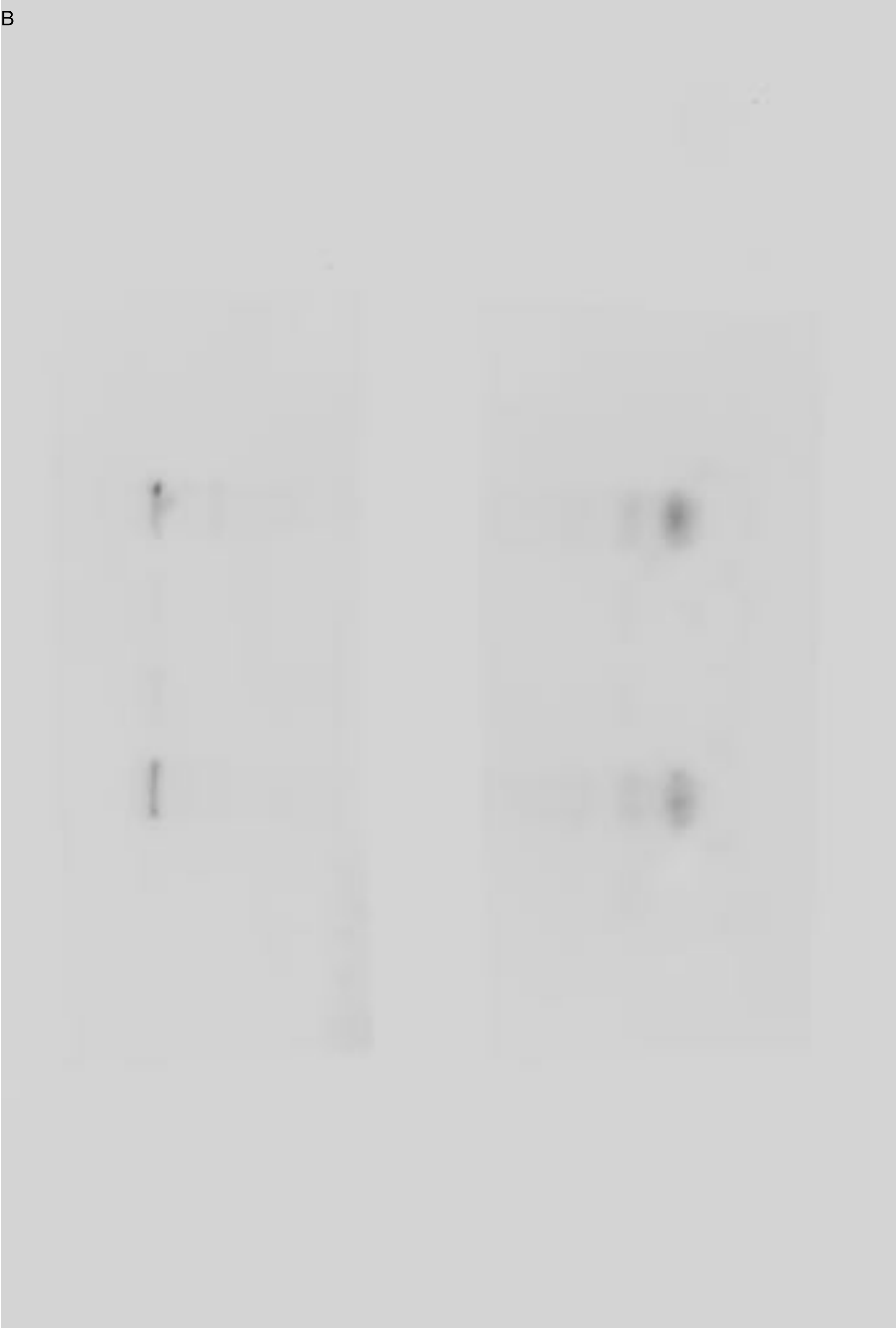

Fig S4B-1

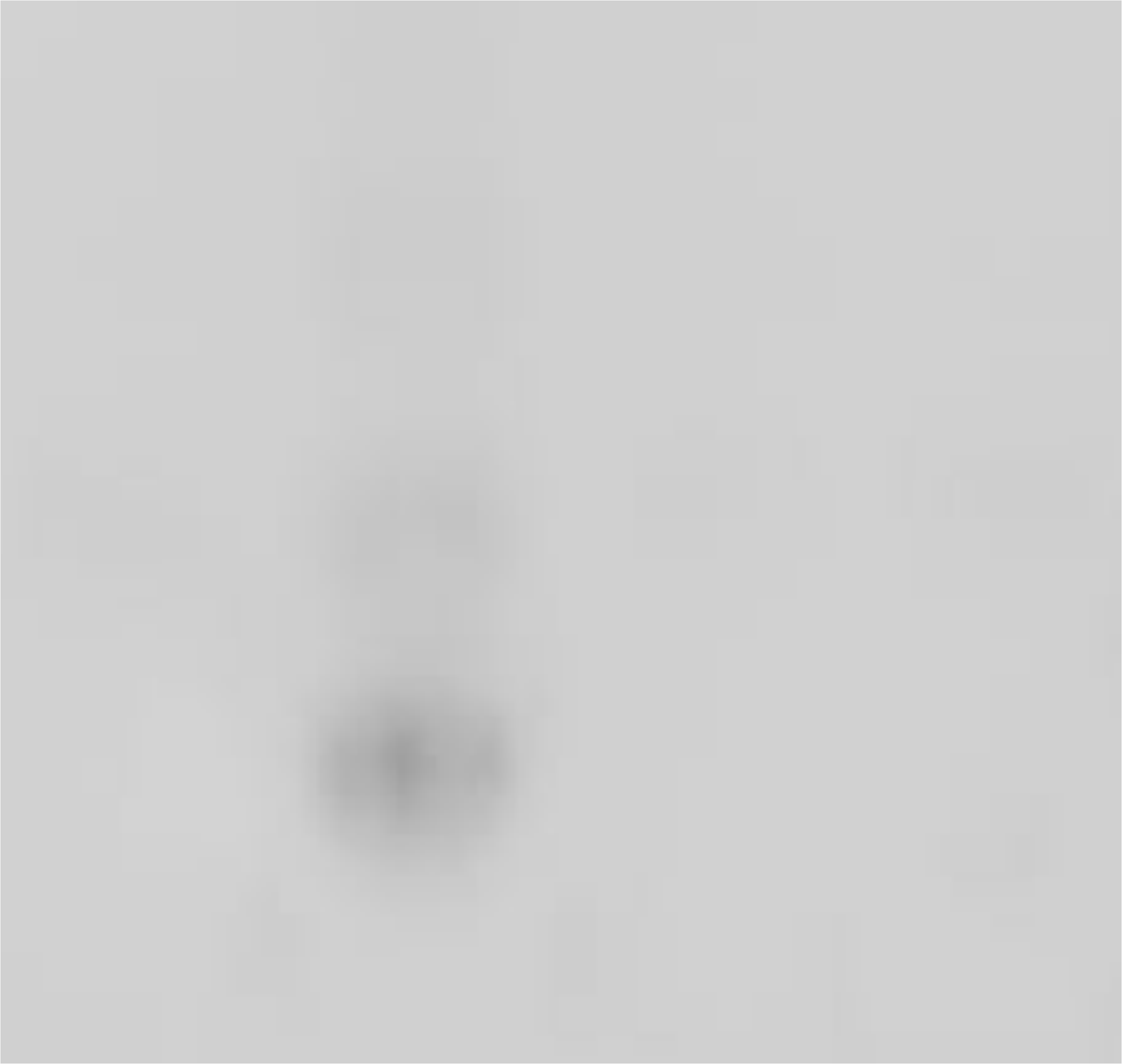

Fig S6A

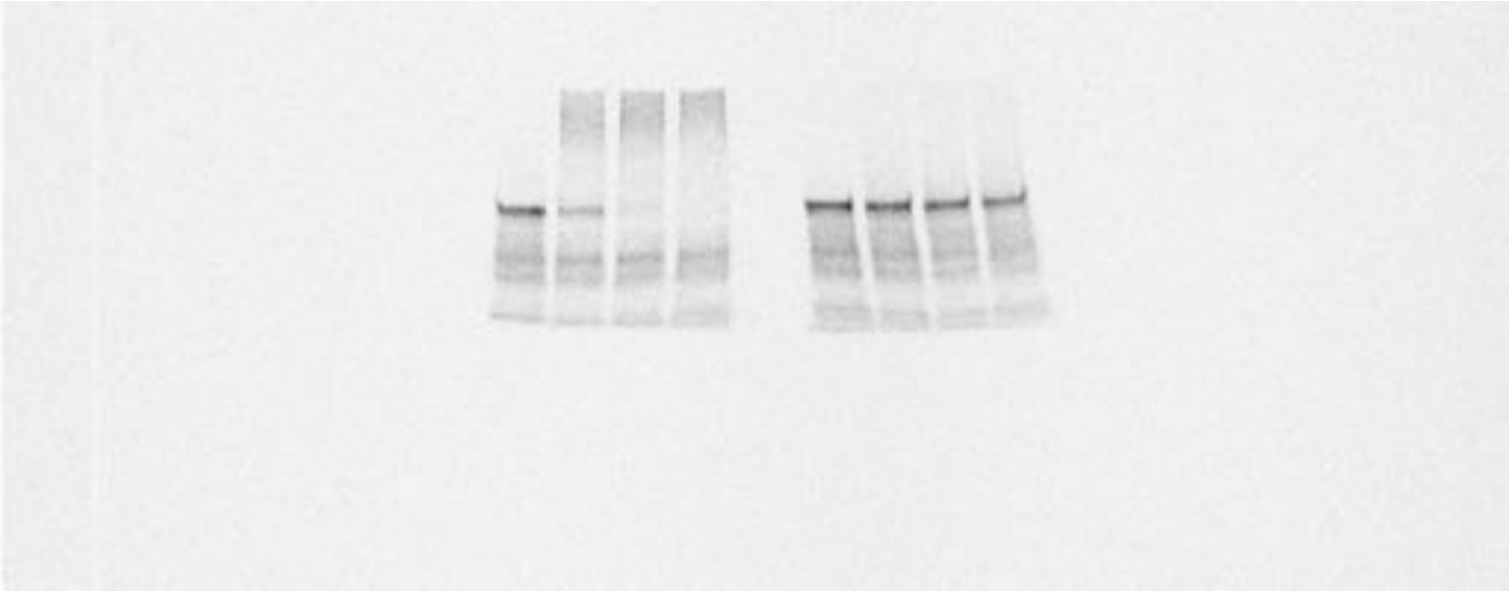

Fig S6A-1

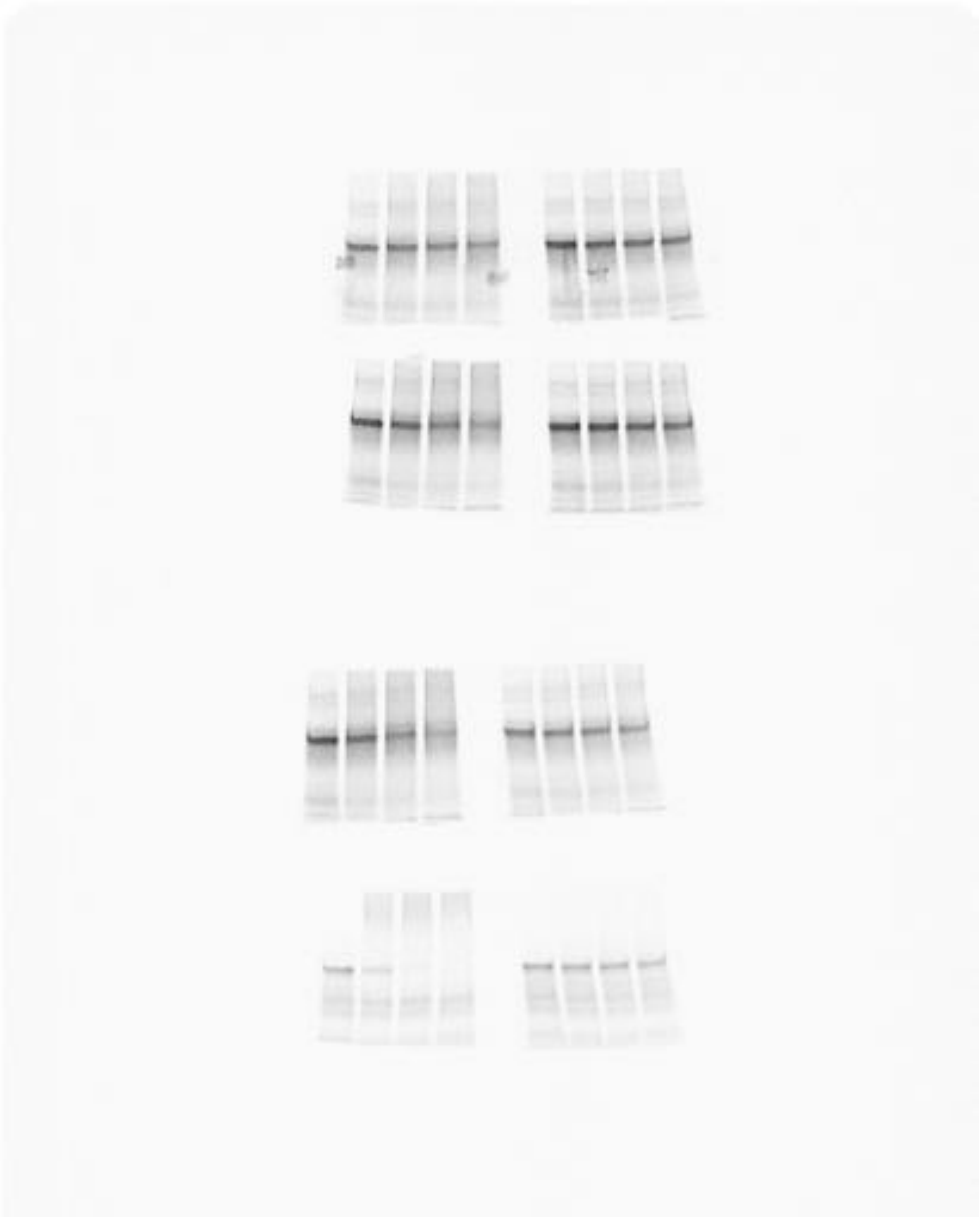

Fig S8A

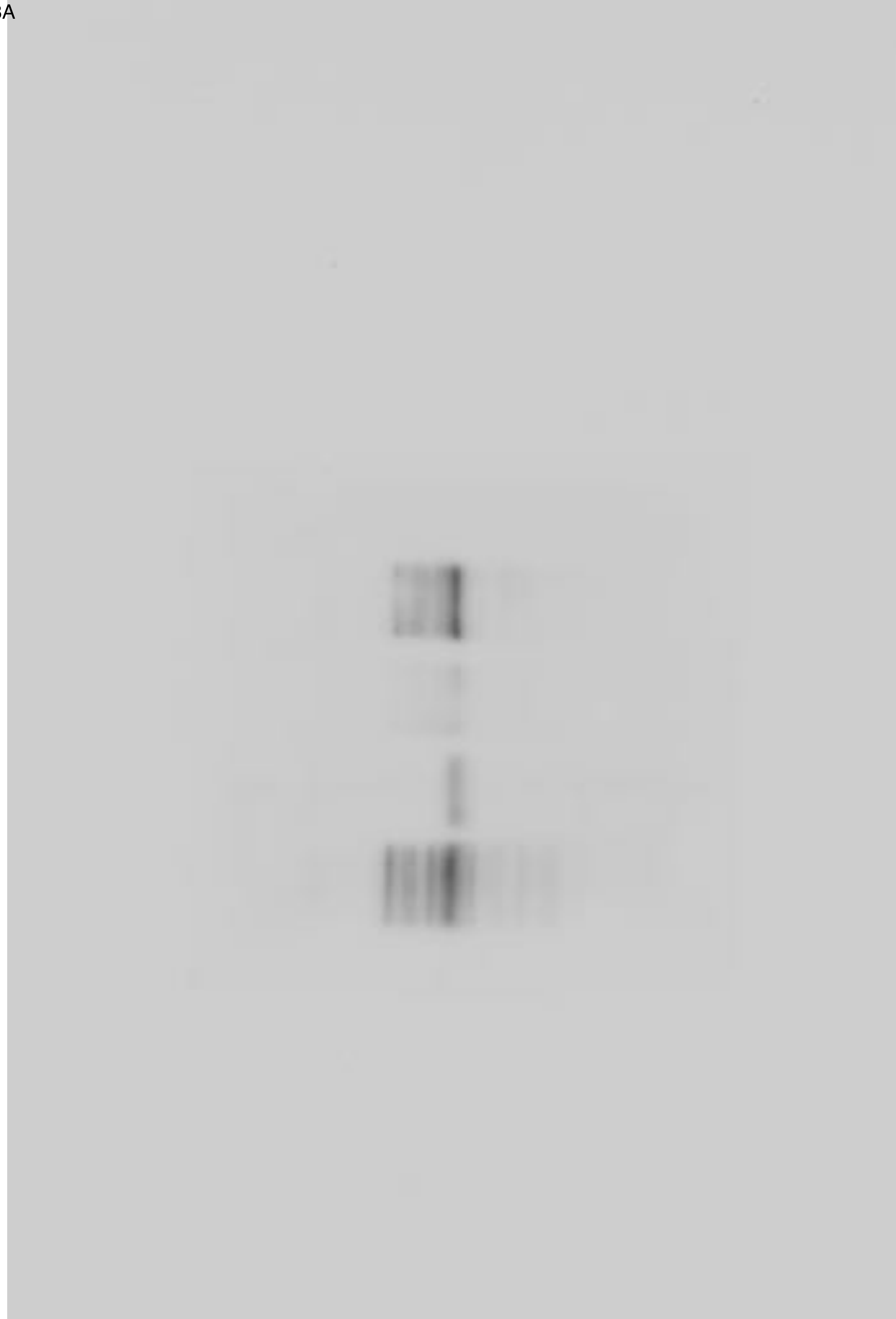

Fig S8B

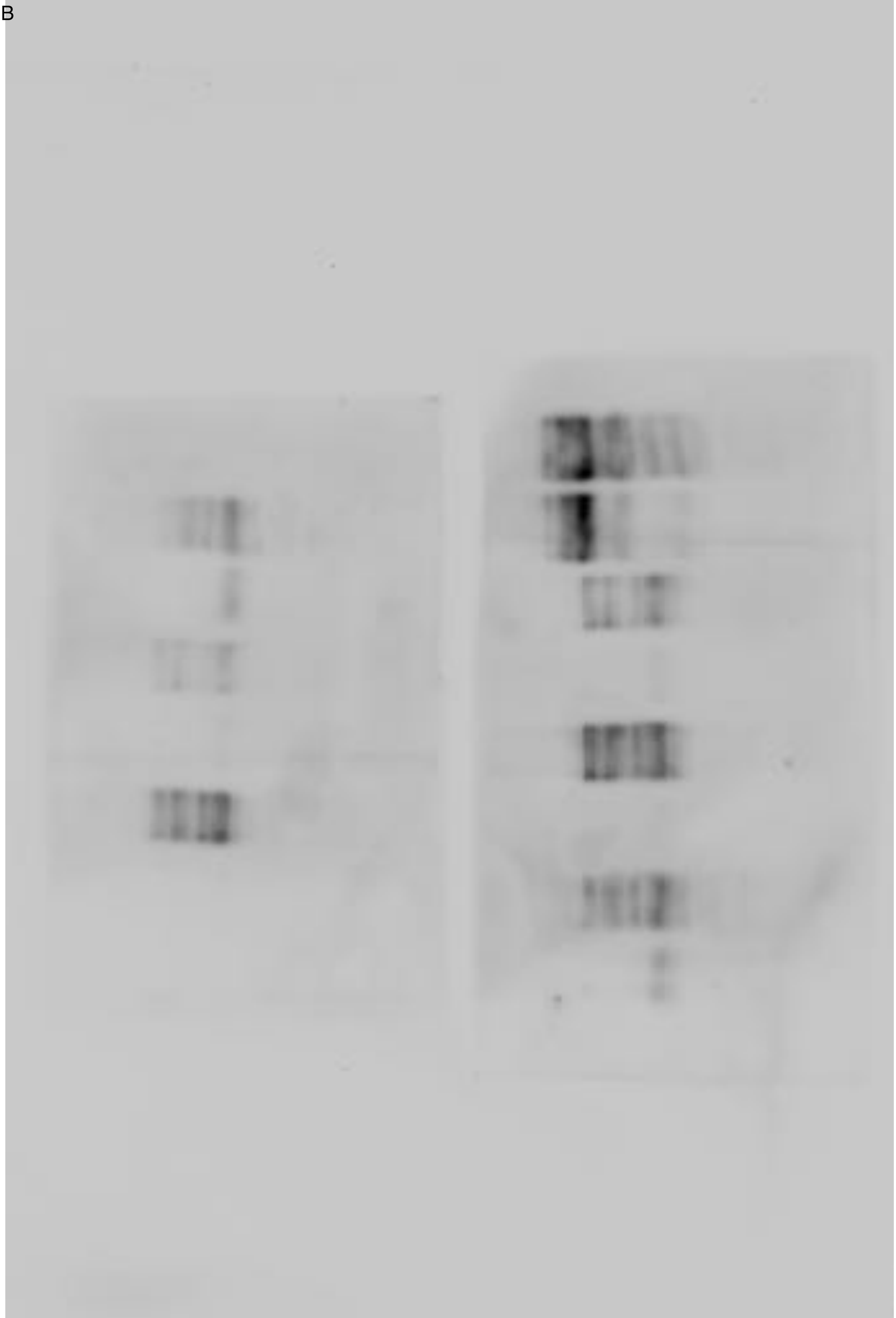

Supplement: S9 Fig — Image files and notebook scans are provided in sequential order as they appear in the paper. (PDF) [file pone.0198930.s009.pdf]
