# Supplementary material for: Peptide inhibitors of the anaphase promoting-complex that cause sensitivity to microtubule poison
Source: PLoS One. 2018 Jun 8;13(6):e0198930. doi: 10.1371/journal.pone.0198930 (PMC5993284; doi:10.1371/journal.pone.0198930)
Supplement: S1 Table — Standard deviations (sd) were derived from curve fitting a 4-parameter Hill equation to the titration data for each subunit or complex. (DOC) [file pone.0198930.s010.doc]

**S1 Table.**

| **Mitotic Checkpoint Complex subunit(s)** | **IC_50_ ± sd** | **Hill coefficient ± sd** |
| --- | --- | --- |
| **Mad2** | 2.3 ± 0.5 μM | 2.4 ± 0.3 |
| **Bub3** | 8.7 ± 2.4 μM | 2.2 ± 0.4 |
| **Mad3** | 240 ± 10 nM | 5.0 ± 0.9 |
| **Mad3-Bub3 complex** | 89 ± 4 nM | 7.3 ± 2.6 |
| **Mad3-Bub3 complex + 0.5 μM Mad2** | 70 ± 3 nM | 8.2 ±1.9 |
