# Supplementary material for: Peptide inhibitors of the anaphase promoting-complex that cause sensitivity to microtubule poison
Source: PLoS One. 2018 Jun 8;13(6):e0198930. doi: 10.1371/journal.pone.0198930 (PMC5993284; doi:10.1371/journal.pone.0198930)
Supplement: S2 Table — (DOC) [file pone.0198930.s011.doc]

**S2 Table.**

| **IC_50_ ± sd** | **Bub3**  **8.7 ± 2.4 μM** | **Mad3**  **0.24 ± 0.01 μM** | **Mad3-Bub3**  **0.089 ± 0.004 μM** | **Mad3-Bub3 + 0.5 μM Mad2**  **0.07 ± 0.003 μM** |
| --- | --- | --- | --- | --- |
| **Mad2**  **2.3 ± 0.5 μM** | 0.0106 | 0.0020 | 0.0016 | 0.0015 |
| **Bub3**  **8.7 ± 2.4 μM** |  | 0.0036 | 0.0034 | 0.0034 |
| **Mad3**  **0.24 ± 0.01 μM** |  |  | 0.0001 | 0.0001 |
| **Mad3-Bub3**  **0.089 ± 0.004 μM** |  |  |  | 0.0028 |
