# Supplementary material for: Peptide inhibitors of the anaphase promoting-complex that cause sensitivity to microtubule poison
Source: PLoS One. 2018 Jun 8;13(6):e0198930. doi: 10.1371/journal.pone.0198930 (PMC5993284; doi:10.1371/journal.pone.0198930)
Supplement: S3 Table — (DOC) [file pone.0198930.s012.doc]

**S3 Table.**

| **Hill coefficient ± sd** | **Bub3**  **2.2 ± 0.4** | **Mad3**  **5.0 ± 0.9** | **Mad3-Bub3**  **7.3 ± 2.6** | **Mad3-Bub3 + 0.5 μM Mad2**  **8.2 ± 1.9** |
| --- | --- | --- | --- | --- |
| **Mad2**  **2.4 ± 0.3** | 0.5265 | 0.0090 | 0.0316 | 0.0064 |
| **Bub3**  **2.2 ± 0.4** |  | 0.0079 | 0.0284 | 0.0059 |
| **Mad3**  **5.0 ± 0.9** |  |  | 0.2212 | 0.0578 |
| **Mad3-Bub3**  **7.3 ± 2.6** |  |  |  | 0.6536 |
