# Supplementary material for: Peptide inhibitors of the anaphase promoting-complex that cause sensitivity to microtubule poison
Source: PLoS One. 2018 Jun 8;13(6):e0198930. doi: 10.1371/journal.pone.0198930 (PMC5993284; doi:10.1371/journal.pone.0198930)
Supplement: S4 Table — 35S-labeled protein amounts were measured after 2 hours of protein synthesis via IVT/T. The cdc20-120 mutant allele contained a single amino acid change at P210, where as the isolated genetic by-pass allele had additional mutations leading the changes at residues T226S and T247I. We chose to focus on the single P210S residue change to simplify the functional analysis of this individual residue, and because it lies within the defined Mad2-binding motif between amino acids 197–223, whereas the T226S change is relatively conservative and lies outside of the defined motif. To mark this difference, we denote this single change amino acid residue allele as cdc20-120*. (DOC) [file pone.0198930.s013.doc]

**S4 Table.**

| **mutant *cdc20***  **alleles** | **mutated sequences** | **changed amino acid residue(s)** | **^35^S-labeled protein levels by produced by IVT/T**  **(*t*-test *p*-values versus wild type) (n = 3)** |
| --- | --- | --- | --- |
| ***cdc20-cb*** | ATTCCA→GCTGCA | I147A, P148A | 0.1894 |
| ***cdc20-D197A*** | ATA→CTA | D197A | 0.2298 |
| ***cdc20-L203A*** | CTA→GCA | L203A | 0.9771 |
| ***cdc20-127*** | TAC→AAC | Y205N | 0.0831 |
| ***cdc20-106*** | CCA→CAA | P209Q | 0.9164 |
| ***cdc20-107*** | CCA→CTA | P210L | 0.0874 |
| ***cdc20-120**** | CCA→TCA | P210S | 0.0633 |
