# Supplementary material for: Peptide inhibitors of the anaphase promoting-complex that cause sensitivity to microtubule poison
Source: PLoS One. 2018 Jun 8;13(6):e0198930. doi: 10.1371/journal.pone.0198930 (PMC5993284; doi:10.1371/journal.pone.0198930)
Supplement: S5 Table — Time points of 15, 30, and 60 minutes are across the top, and each mutant allele tested is on the left. Boxes shaded in grey indicate a p-value < 0.05 relative to wild type Cdc20. The mutant alleles Cdc20-127 (Y205N), Cdc20-L203A, and the control mutant Cdc20-CB (I147A, P148A) all consistently displayed a decrease in APC activity (n = 3). (DOC) [file pone.0198930.s014.doc]

**S5 Table.**

|  | **15 minutes** | **30 minutes** | **60 minutes** |
| --- | --- | --- | --- |
| **Cdc20-127**  **(Y205N)** | < 0.0001 | < 0.0001 | < 0.0001 |
| **Cdc20-106**  **(P209Q)** | 0.252 | 0.803 | 0.447 |
| **Cdc20-107**  **(P210L)** | 0.878 | 0.855 | 0.024 |
| **Cdc20-120***  **(P210S)** | 0.551 | 0.688 | 0.175 |
| **Cdc20-D197A** | 0.829 | 0.159 | 0.066 |
| **Cdc20-L203A** | 0.007 | 0.020 | 0.058 |
| **Cdc20-CB**  **(I147A, P148A)** | 0.0001 | < 0.0001 | 0.0002 |
