# Supplementary material for: Peptide inhibitors of the anaphase promoting-complex that cause sensitivity to microtubule poison
Source: PLoS One. 2018 Jun 8;13(6):e0198930. doi: 10.1371/journal.pone.0198930 (PMC5993284; doi:10.1371/journal.pone.0198930)
Supplement: S6 Table — Time points are on the left, and mutant alleles tested are labeled across the top. Grey boxes indicate a p-value > 0.05 relative to wild type Cdc20. The mutant alleles Cdc20-127 (Y205N) and Cdc20-106 (P209Q) consistently displayed APC activity in the presence of Mad2 (n = 3). Cdc20-107 (P210L) was not tested, as the mutation in this residue is at the same amino acid residue as the Cdc20-120* (P210S) mutant allele. (DOC) [file pone.0198930.s015.doc]

**S6 Table.**

| **Time**  **(minutes)** | **Cdc20-127**  **(Y205N)** | **Cdc20-106**  **(P209Q)** | **Cdc20-120* (P210S)** | **Cdc20-D197A** | **Cdc20-L203A** |
| --- | --- | --- | --- | --- | --- |
| **15** | 0.733 | 0.496 | 0.008 | 0.003 | 0.0003 |
| **30** | 0.371 | 0.260 | 0.001 | 0.0005 | 0.0006 |
| **60** | 0.417 | 0.705 | 0.0001 | 0.0039 | 0.0026 |
